# Supplementary material for: In Silico Genome-Wide Characterisation of the Lipid Transfer Protein Multigenic Family in Sunflower (H. annuus L.)
Source: Plants (Basel). 2022 Feb 28;11(5):664. doi: 10.3390/plants11050664 (PMC8912825; doi:10.3390/plants11050664)
Supplement: Supplementary file 1 [file plants-11-00664-s001.zip › plants-1597355-supplementary.pdf]

Supplementary Figure S1:Possible pseudogene belonging to Lipid Transfer Proteins (LTP) family in *Helianthus annuus*

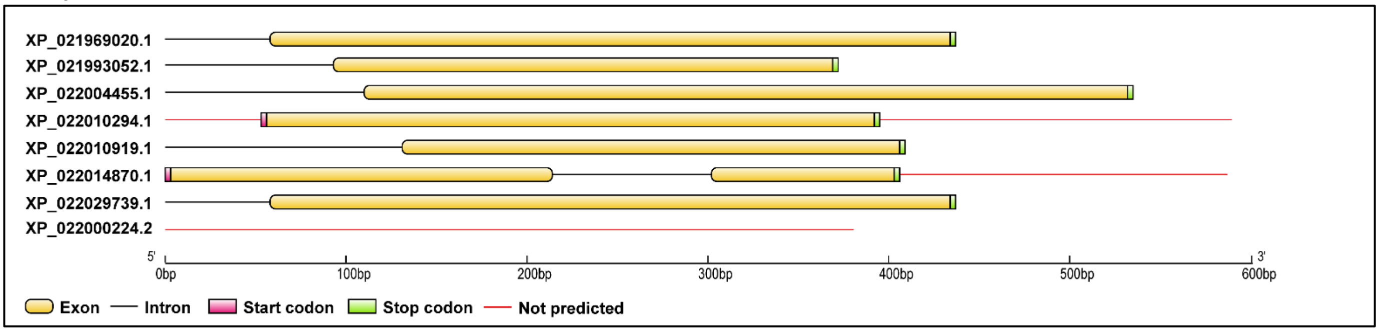

Supplementary Table S1: List of lipid transfer proteins in *H. annuus* genome

NCBI protein id	NCBI locus id	Chromosome number	Locus Start	Locus Stop	Strand	Length	Number of exons
XP_022039085.1	LOC110941729	chromosome 1	581436	582257	+	118	2
XP_022033162.1	LOC110934348	chromosome 1	585234	586422	-	119	2
XP_022039074.2	LOC110941719	chromosome 1	624730	625499	-	119	2
XP_022014870.1	LOC110914383	chromosome 1	7597209	7598579	-	195	4
XP_022013182.1	LOC110912707	chromosome 1	7610565	7612215	-	182	3
XP_022013183.1	LOC110912707	chromosome 1	7610654	7612215	-	160	3
XP_021992525.1	LOC110889321	chromosome 1	7885930	7887022	-	183	3
XP_021993283.1	LOC110890000	chromosome 1	61013979	61014542	-	187	1
XP_021978807.1	LOC110874493	chromosome 1	108232434	108233698	+	120	2
XP_021981388.1	LOC110877546	chromosome 1	128458943	128459702	+	162	2
XP_021981395.1	LOC110877546	chromosome 1	128458943	128459702	+	137	3
XP_021981407.1	LOC110877557	chromosome 1	128462583	128463572	+	166	2
XP_022022835.1	LOC110922945	chromosome 2	138203574	138207310	+	174	3
XP_022026963.1	LOC110928012	chromosome 3	133526330	133526785	+	151	1
XP_022026964.1	LOC110928013	chromosome 3	133536088	133536504	-	138	1
XP_022029740.1	LOC110930697	chromosome 3	169779341	169780063	-	114	2
XP_022029738.1	LOC110930696	chromosome 3	169826692	169846789	-	118	2
XP_022029739.1	LOC110930696	chromosome 3	169826692	169827534	-	117	2
XP_022035551.1	LOC110937439	chromosome 4	179020078	179021301	-	195	3
XP_022035552.1	LOC110937439	chromosome 4	179020078	179021301	-	193	3
XP_022035384.1	LOC110937295	chromosome 4	183245545	183246245	-	198	2
XP_022021911.1	LOC110921897	chromosome 5	106564244	106565720	+	189	3
XP_022021912.1	LOC110921897	chromosome 5	106564244	106565720	+	165	2
XP_022021913.1	LOC110921897	chromosome 5	106564244	106565625	+	164	3
XP_022021914.1	LOC110921897	chromosome 5	106564244	106565638	+	163	3
XP_021969020.1	LOC110864286	chromosome 6	896081	896470	+	129	1
XP_021968895.1	LOC110864082	chromosome 6	81669868	81670637	+	123	2
XP_022037775.1	LOC110940540	chromosome 7	57654493	57654786	-	97	1
XP_021972810.1	LOC110868035	chromosome 7	94502385	94502877	+	116	2
XP_021973011.1	LOC110868209	chromosome 7	97575975	97576268	-	97	1
XP_021973316.1	LOC110868466	chromosome 7	125394885	125396628	+	182	3
XP_021973854.1	LOC110868899	chromosome 7	141936595	141937647	-	118	2
XP_021973855.1	LOC110868900	chromosome 7	141940297	141940718	-	112	2
XP_021976022.1	LOC110871621	chromosome 8	2652007	2655412	+	189	2
XP_021981626.1	LOC110877736	chromosome 9	56193955	56195123	+	231	3
XP_021978842.1	LOC110874594	chromosome 9	120415585	120416034	-	149	1
XP_035833070.1	LOC110876985	chromosome 9	187701647	187702501	-	184	3
XP_021984981.2	LOC110880848	chromosome 10	17259866	17260560	+	165	3
XP_021987457.1	LOC110884099	chromosome 10	18149573	18150784	-	238	4
XP_021987458.1	LOC110884099	chromosome 10	18149573	18150784	-	234	4
XP_021985239.1	LOC110881217	chromosome 10	36339358	36339804	-	119	2
XP_021988953.1	LOC110885559	chromosome 10	55844623	55846066	-	218	3
XP_021986500.1	LOC110882920	chromosome 10	154153690	154154007	-	105	1
XP_021993222.1	LOC110889963	chromosome 11	31344829	31346383	-	324	2
XP_021993221.1	LOC110889962	chromosome 11	31365864	31366954	-	299	2
XP_021993220.1	LOC110889960	chromosome 11	31457171	31458250	-	290	2
XP_021995117.1	LOC110892047	chromosome 11	50026964	50027691	+	141	2
XP_021993622.1	LOC110890334	chromosome 11	50482291	50483656	-	285	2
XP_021995116.1	LOC110892046	chromosome 11	50538364	50538849	-	161	1
XP_021993620.1	LOC110890331	chromosome 11	50546644	50547165	-	173	1
XP_021993621.2	LOC110890332	chromosome 11	50572849	50573340	-	163	1
XP_021993551.1	LOC110890261	chromosome 11	52380677	52384009	+	181	3
XP_035835830.1	LOC110863966	chromosome 11	108158307	108158677	-	94	2
XP_021988229.1	LOC110884826	chromosome 11	113298018	113298768	-	118	2
XP_021993052.1	LOC110889796	chromosome 11	125753126	125753678	-	115	2
XP_021989733.1	LOC110886266	chromosome 11	144490130	144491111	+	173	2
XP_021989734.1	LOC110886267	chromosome 11	144569037	144570202	+	173	2
XP_021989735.1	LOC110886269	chromosome 11	144702358	144703486	+	173	2
XP_021989736.1	LOC110886270	chromosome 11	144721120	144722287	+	173	3
XP_021989737.1	LOC110886271	chromosome 11	144767805	144769027	+	173	2
XP_021999258.1	LOC110896126	chromosome 12	160209484	160211308	+	179	2
XP_022000224.2	LOC110897803	chromosome 13	50218842	50219201	+	119	1
XP_022000196.1	LOC110897772	chromosome 13	56847763	56852051	-	147	3
XP_022001254.1	LOC110898730	chromosome 13	93772080	93773587	-	209	3
XP_022004979.1	LOC110903125	chromosome 13	108695467	108695979	+	170	1
XP_022004980.1	LOC110903127	chromosome 13	108820267	108820719	+	150	1
XP_022002421.1	LOC110899840	chromosome 13	152431601	152432072	-	116	2
XP_022002420.1	LOC110899839	chromosome 13	152438050	152445045	-	116	2
XP_022002419.1	LOC110899837	chromosome 13	152481868	152482341	+	116	2
XP_022002418.1	LOC110899836	chromosome 13	152491525	152492008	-	116	2
XP_022004455.1	LOC110902020	chromosome 13	152522835	152530074	+	152	3
XP_022002417.1	LOC110899835	chromosome 13	152577385	152577868	-	116	2
XP_022002416.1	LOC110899834	chromosome 13	152600693	152601176	-	116	2

XP_022002415.1	LOC110899830	chromosome 13	152696656	152697139	-	116	2
XP_021984766.1	LOC110880578	chromosome 14	79297053	79298729	-	147	3
XP_022009213.1	LOC110908581	chromosome 14	81531614	81532253	-	173	2
XP_035838717.1	LOC110908581	chromosome 14	81531614	81532253	-	169	2
XP_022008275.1	LOC110907631	chromosome 14	155413680	155414231	-	143	2
XP_022008276.2	LOC110907632	chromosome 14	155425541	155426767	-	408	1
XP_022006327.1	LOC110904777	chromosome 14	165399587	165400713	-	135	2
XP_022006668.1	LOC110905097	chromosome 14	171383331	171383698	+	88	2
XP_022010291.1	LOC110909857	chromosome 15	1698198	1699659	+	117	2
XP_022010292.1	LOC110909858	chromosome 15	1705435	1706092	+	133	2
XP_022013558.1	LOC110913007	chromosome 15	1712070	1712680	+	116	2
XP_022010296.1	LOC110909863	chromosome 15	1717089	1717690	+	116	2
XP_022010294.1	LOC110909860	chromosome 15	1719527	1720412	+	113	2
XP_022010295.2	LOC110909861	chromosome 15	1744980	1745644	+	117	2
XP_022013560.1	LOC110913009	chromosome 15	1747245	1748158	+	115	2
XP_022010919.1	LOC110910600	chromosome 15	11013390	11035335	-	121	3
XP_022010101.1	LOC110909475	chromosome 15	27816228	27817692	+	115	2
XP_022006926.1	LOC110905651	chromosome 15	27857052	27857940	+	116	2
XP_022006927.1	LOC110905652	chromosome 15	27859102	27859845	+	116	2
XP_022010616.1	LOC110910229	chromosome 15	120041266	120041773	+	128	2
XP_022015084.1	LOC110914604	chromosome 15	158011912	158012277	-	121	1
XP_022013239.1	LOC110912748	chromosome 15	158153608	158154982	+	276	2
XP_022018622.1	LOC110918654	chromosome 16	153809761	153810196	-	110	2
XP_022029622.1	LOC110930599	chromosome 16	155393196	155393558	-	120	1
XP_022034230.1	LOC110936193	chromosome 17	42192992	42193416	+	117	2
XP_022005608.1	LOC110904098	chromosome 17	119157650	119157937	-	95	1
XP_035842399.1	LOC110922502	chromosome 17	157630148	157663971	-	135	7
XP_022022487.2	LOC110922502	chromosome 17	157657306	157661254	-	117	2

Supplementary Table S2: amino acid motifs discovered by MEME amongst HaLTPs

MEME motifs	Consensus sequences
Red motif	CCNGVKGLNAAAKTTADRQAACGCLKSAYSSJSGI
Grey motif	YAEASTCGQVLSSLSPCLNYLTGGGSVPP
Green motif	AGNAASLPGKCGVNIPYKISPSTDCSKVQ
Purple motif	NGGGSSLGLNINQTLALELPKACNVQTPP
Yellow motif	QKEZQLLQQCCQZLQNVEEQCQCEAVKQVFRZAQQQVQQQQ

Supplementary Table S3: Blastp Similarity of Group 2, Group 3 and Group 4 HaLTPs on swissprot database.

NCBI protein id	UniprotKB id	Similarity Percentage	UniprotKB description	Pfam id	Pfam Description	HaLTPs Group
XP_021993220.1	sp|P15461|2SS5_HELAN	84.25	2SS5_HELAN 2S seed storage protein OS=Helianthus annuus OX=4232 GN=HAG5 PE=1 SV=1	PF00234	Protease inhibitor/seed storage/LTP family	
XP_021993221.1	sp|P15461|2SS5_HELAN	96.63	2SS5_HELAN 2S seed storage protein OS=Helianthus annuus OX=4232 GN=HAG5 PE=1 SV=1	PF00234	Protease inhibitor/seed storage/LTP family	
XP_021993222.1	sp|P15461|2SS5_HELAN	58.15	2SS5_HELAN 2S seed storage protein OS=Helianthus annuus OX=4232 GN=HAG5 PE=1 SV=1	PF00234	Protease inhibitor/seed storage/LTP family	
XP_021993283.1	sp|P01045|KNG2_BOVIN	47.5	KNG2_BOVIN Kininogen-2 OS=Bos taurus OX=9913 GN=KNG2 PE=1 SV=1	NA	NA	
XP_021993620.1	sp|P15461|2SS5_HELAN	60.67	2SS5_HELAN 2S seed storage protein OS=Helianthus annuus OX=4232 GN=HAG5 PE=1 SV=1	PF00234	Protease inhibitor/seed storage/LTP family	
XP_021993621.2	sp|P15461|2SS5_HELAN	61.9	2SS5_HELAN 2S seed storage protein OS=Helianthus annuus OX=4232 GN=HAG5 PE=1 SV=1	PF00234	Protease inhibitor/seed storage/LTP family	
XP_021993622.1	sp|P15461|2SS5_HELAN	76.73	2SS5_HELAN 2S seed storage protein OS=Helianthus annuus OX=4232 GN=HAG5 PE=1 SV=1	PF00234	Protease inhibitor/seed storage/LTP family	
XP_021995116.1	sp|P15461|2SS5_HELAN	64.19	2SS5_HELAN 2S seed storage protein OS=Helianthus annuus OX=4232 GN=HAG5 PE=1 SV=1	PF00234	Protease inhibitor/seed storage/LTP family	
XP_022004979.1	sp|Q39649|2SS_CUCMA	26.51	2SS_CUCMA 2S albumin OS=Cucurbita maxima OX=3661 PE=1 SV=1	PF00234	Protease inhibitor/seed storage/LTP family	Group 2
XP_022004980.1	sp|Q39649|2SS_CUCMA	28.95	2SS_CUCMA 2S albumin OS=Cucurbita maxima OX=3661 PE=1 SV=1	PF00234	Protease inhibitor/seed storage/LTP family	
XP_022008275.1	sp|Q39649|2SS_CUCMA	29.85	2SS_CUCMA 2S albumin OS=Cucurbita maxima OX=3661 PE=1 SV=1	PF00234	Protease inhibitor/seed storage/LTP family	
XP_022008276.2	sp|Q39649|2SS_CUCMA	28.06	2SS_CUCMA 2S albumin OS=Cucurbita maxima OX=3661 PE=1 SV=1	PF00234	Protease inhibitor/seed storage/LTP family	
XP_022013239.1	sp|P15461|2SS5_HELAN	39.85	2SS5_HELAN 2S seed storage protein OS=Helianthus annuus OX=4232 GN=HAG5 PE=1 SV=1	PF00234	Protease inhibitor/seed storage/LTP family	
XP_022015084.1	sp|P0C8Y8|2SS2_BEREX	29.91	2SS2_BEREX 2S sulfur-rich seed storage protein 2 OS=Bertholletia excelsa OX=3645 GN=BE2S2 PE=3 SV=1	PF00234	Protease inhibitor/seed storage/LTP family	
XP_022026963.1	sp|Q4GWU5|SFTI1_HELAN	100	SFTI1_HELAN Trypsin inhibitor 1 OS=Helianthus annuus OX=4232 GN=sfti1 PE=1 SV=1	PF00234	Protease inhibitor/seed storage/LTP family	
XP_022026964.1	sp|Q9FH31|2SS5_ARATH	29.75	2SS5_ARATH 2S seed storage protein 5 OS=Arabidopsis thaliana OX=3702 GN=SESA5 PE=1 SV=1	PF00234	Protease inhibitor/seed storage/LTP family	
XP_035833070.1	sp|P15461|2SS5_HELAN	85.83	2SS5_HELAN 2S seed storage protein OS=Helianthus annuus OX=4232 GN=HAG5 PE=1 SV=1	PF00234	Protease inhibitor/seed storage/LTP family	
XP_021973011.1	sp|P82353|NLTP2_PRUAR	58.82	NLTP2_PRUAR Non-specific lipid-transfer protein 2 OS=Prunus armeniaca OX=36596 PE=1 SV=1	PF14368	Probable lipid transfer	
XP_021973854.1	sp|Q8W453|DIRL1_ARATH	36.11	DIRL1_ARATH Putative lipid-transfer protein DIR1 OS=Arabidopsis thaliana OX=3702 GN=DIR1 PE=1 SV=1	PF14368	Probable lipid transfer	
XP_021973855.1	sp|Q8W453|DIRL1_ARATH	31.94	DIRL1_ARATH Putative lipid-transfer protein DIR1 OS=Arabidopsis thaliana OX=3702 GN=DIR1 PE=1 SV=1	PF14368	Probable lipid transfer	
XP_021978842.1	sp|Q9FFY3|LTG30_ARATH	53.47	LTG30_ARATH Non-specific lipid transfer protein GPI-anchored 30 OS=Arabidopsis thaliana OX=3702 GN=LTPG30 PE=2 SV=1	PF14368	Probable lipid transfer	
XP_021986500.1	sp|Q8W453|DIRL1_ARATH	44.29	DIRL1_ARATH Putative lipid-transfer protein DIR1 OS=Arabidopsis thaliana OX=3702 GN=DIR1 PE=1 SV=1	PF14368	Probable lipid transfer	
XP_021993052.1	sp|Q8W453|DIRL1_ARATH	35.38	DIRL1_ARATH Putative lipid-transfer protein DIR1 OS=Arabidopsis thaliana OX=3702 GN=DIR1 PE=1 SV=1	PF14368	Probable lipid transfer	
XP_021995117.1	sp|P23110|2SS8_HELAN	98.58	2SS8_HELAN Albumin-8 OS=Helianthus annuus OX=4232 PE=1 SV=1	PF00234	Protease inhibitor/seed storage/LTP family	Group 3
XP_022000196.1	sp|Q6NLF7|LTPG7_ARATH	42.76	LTPG7_ARATH Non-specific lipid transfer protein GPI-anchored 7 OS=Arabidopsis thaliana OX=3702 GN=LTPG7 PE=2 SV=1	PF14368	Probable lipid transfer	
XP_022005608.1	sp|P82353|NLTP2_PRUAR	60.29	NLTP2_PRUAR Non-specific lipid-transfer protein 2 OS=Prunus armeniaca OX=36596 PE=1 SV=1	PF14368	Probable lipid transfer	
XP_022006327.1	sp|Q8W453|DIRL1_ARATH	36.11	DIRL1_ARATH Putative lipid-transfer protein DIR1 OS=Arabidopsis thaliana OX=3702 GN=DIR1 PE=1 SV=1	PF14368	Probable lipid transfer	
XP_022010919.1	sp|Q7EB72|LTG15_ARATH	32.89	LTG15_ARATH Non-specific lipid transfer protein GPI-anchored 15 OS=Arabidopsis thaliana OX=3702 GN=LTPG15 PE=2 SV=1	PF14368	Probable lipid transfer	
XP_022018622.1	sp|Q9M5X7|NLTP_MALDO	34.69	NLTP_MALDO Non-specific lipid-transfer protein OS=Malus domestica OX=3750 GN=MALD3 PE=1 SV=1	PF14368	Probable lipid transfer	
XP_022022835.1	sp|Q6NLF7|LTPG7_ARATH	39.16	LTPG7_ARATH Non-specific lipid transfer protein GPI-anchored 7 OS=Arabidopsis thaliana OX=3702 GN=LTPG7 PE=2 SV=1	PF14368	Probable lipid transfer	
XP_022037775.1	sp|Q8W453|DIRL1_ARATH	49.33	DIRL1_ARATH Putative lipid-transfer protein DIR1 OS=Arabidopsis thaliana OX=3702 GN=DIR1 PE=1 SV=1	PF14368	Probable lipid transfer	
XP_021969020.1	sp|P14009|14KD_DAUCA	54.41	14KD_DAUCA 14 kDa proline-rich protein DC2.15 OS=Daucus carota OX=4039 PE=2 SV=1	PF14547	Hydrophobic seed protein	
XP_021973316.1	sp|O64865|LTG14_ARATH	35.75	LTG14_ARATH Non-specific lipid transfer protein GPI-anchored 14 OS=Arabidopsis thaliana OX=3702 GN=LTPG14 PE=2 SV=1	PF14368	Probable lipid transfer	
XP_021976022.1	sp|Q9C7F7|LTPG1_ARATH	44.59	LTPG1_ARATH Non-specific lipid transfer protein GPI-anchored 1 OS=Arabidopsis thaliana OX=3702 GN=LTPG1 PE=2 SV=1	PF14368	Probable lipid transfer	
XP_021981388.1	sp|Q9LJ86|LTPG5_ARATH	49.69	LTPG5_ARATH Non-specific lipid transfer protein GPI-anchored 5 OS=Arabidopsis thaliana OX=3702 GN=LTPG5 PE=2 SV=1	PF14368	Probable lipid transfer	
XP_021981395.1	sp|Q9LJ86|LTPG5_ARATH	52.1	LTPG5_ARATH Non-specific lipid transfer protein GPI-anchored 5 OS=Arabidopsis thaliana OX=3702 GN=LTPG5 PE=2 SV=1	PF14368	Probable lipid transfer	
XP_021981407.1	sp|Q9LJ86|LTPG5_ARATH	51.79	LTPG5_ARATH Non-specific lipid transfer protein GPI-anchored 5 OS=Arabidopsis thaliana OX=3702 GN=LTPG5 PE=2 SV=1	PF14368	Probable lipid transfer	
XP_021981626.1	sp|Q1G2Y5|LTG21_ARATH	55.63	LTG21_ARATH Non-specific lipid transfer protein GPI-anchored 21 OS=Arabidopsis thaliana OX=3702 GN=LTPG21 PE=2 SV=1	PF14368	Probable lipid transfer	
XP_021984766.1	sp|Q9LJ86|LTPG5_ARATH	48.57	LTPG5_ARATH Non-specific lipid transfer protein GPI-anchored 5 OS=Arabidopsis thaliana OX=3702 GN=LTPG5 PE=2 SV=1	PF14368	Probable lipid transfer	
XP_021984981.2	sp|Q9LJ86|LTPG5_ARATH	49.64	LTPG5_ARATH Non-specific lipid transfer protein GPI-anchored 5 OS=Arabidopsis thaliana OX=3702 GN=LTPG5 PE=2 SV=1	PF14368	Probable lipid transfer	
XP_021987457.1	sp|Q94AX3|LTG16_ARATH	38.16	LTG16_ARATH Non-specific lipid transfer protein GPI-anchored 16 OS=Arabidopsis thaliana OX=3702 GN=LTPG16 PE=2 SV=1	PF14368	Probable lipid transfer	
XP_021987458.1	sp|Q94AX3|LTG16_ARATH	39.29	LTG16_ARATH Non-specific lipid transfer protein GPI-anchored 16 OS=Arabidopsis thaliana OX=3702 GN=LTPG16 PE=2 SV=1	PF14368	Probable lipid transfer	
XP_021988953.1	sp|Q8VYI9|LTG31_ARATH	33.14	LTG31_ARATH Non-specific lipid transfer protein GPI-anchored 31 OS=Arabidopsis thaliana OX=3702 GN=LTPG31 PE=2 SV=1	PF14368	Probable lipid transfer	
XP_021989733.1	sp|Q9LJ86|LTPG5_ARATH	49.12	LTPG5_ARATH Non-specific lipid transfer protein GPI-anchored 5 OS=Arabidopsis thaliana OX=3702 GN=LTPG5 PE=2 SV=1	PF14368	Probable lipid transfer	
XP_021989734.1	sp|Q9LJ86|LTPG5_ARATH	47.37	LTPG5_ARATH Non-specific lipid transfer protein GPI-anchored 5 OS=Arabidopsis thaliana OX=3702 GN=LTPG5 PE=2 SV=1	PF14368	Probable lipid transfer	
XP_021989735.1	sp|Q9LJ86|LTPG5_ARATH	47.37	LTPG5_ARATH Non-specific lipid transfer protein GPI-anchored 5 OS=Arabidopsis thaliana OX=3702 GN=LTPG5 PE=2 SV=1	PF14368	Probable lipid transfer	
XP_021989736.1	sp|Q9LJ86|LTPG5_ARATH	47.95	LTPG5_ARATH Non-specific lipid transfer protein GPI-anchored 5 OS=Arabidopsis thaliana OX=3702 GN=LTPG5 PE=2 SV=1	PF14368	Probable lipid transfer	
XP_021989737.1	sp|Q9LJ86|LTPG5_ARATH	48.54	LTPG5_ARATH Non-specific lipid transfer protein GPI-anchored 5 OS=Arabidopsis thaliana OX=3702 GN=LTPG5 PE=2 SV=1	PF14368	Probable lipid transfer	
XP_021992525.1	sp|Q7EB72|LTG15_ARATH	42.13	LTG15_ARATH Non-specific lipid transfer protein GPI-anchored 15 OS=Arabidopsis thaliana OX=3702 GN=LTPG15 PE=2 SV=1	PF14368	Probable lipid transfer	Group 4
XP_021993551.1	sp|Q8VYI9|LTG31_ARATH	50.55	LTG31_ARATH Non-specific lipid transfer protein GPI-anchored 31 OS=Arabidopsis thaliana OX=3702 GN=LTPG31 PE=2 SV=1	PF14368	Probable lipid transfer	
XP_021999258.1	sp|Q9C7F7|LTPG1_ARATH	40.27	LTPG1_ARATH Non-specific lipid transfer protein GPI-anchored 1 OS=Arabidopsis thaliana OX=3702 GN=LTPG1 PE=2 SV=1	PF14368	Probable lipid transfer	
XP_022000224.2	sp|Q9FIT2|NLTPE_ARATH	56.36	NLTPE_ARATH Putative non-specific lipid-transfer protein 14 OS=Arabidopsis thaliana OX=3702 GN=LTP14 PE=3 SV=2	NA	NA	
XP_022001254.1	sp|O64864|LTG13_ARATH	38.69	LTG13_ARATH Non-specific lipid transfer protein GPI-anchored 13 OS=Arabidopsis thaliana OX=3702 GN=LTPG13 PE=2 SV=1	PF14368	Probable lipid transfer	
XP_022006668.1	sp|Q38737|FIL1_ANTMA	50	FIL1_ANTMA Stamen-specific protein FIL1 OS=Antirrhinum majus OX=4151 GN=FIL1 PE=2 SV=1	PF00234	Protease inhibitor/seed storage/LTP family	
XP_022009213.1	sp|Q9LJ86|LTPG5_ARATH	59.26	LTPG5_ARATH Non-specific lipid transfer protein GPI-anchored 5 OS=Arabidopsis thaliana OX=3702 GN=LTPG5 PE=2 SV=1	PF14368	Probable lipid transfer	
XP_022013182.1	sp|Q7EB72|LTG15_ARATH	41.11	LTG15_ARATH Non-specific lipid transfer protein GPI-anchored 15 OS=Arabidopsis thaliana OX=3702 GN=LTPG15 PE=2 SV=1	PF14368	Probable lipid transfer	
XP_022013183.1	sp|Q9LJ86|LTPG5_ARATH	51.64	LTPG5_ARATH Non-specific lipid transfer protein GPI-anchored 5 OS=Arabidopsis thaliana OX=3702 GN=LTPG5 PE=2 SV=1	PF14368	Probable lipid transfer	
XP_022014870.1	sp|Q9LJ86|LTPG5_ARATH	41.82	LTPG5_ARATH Non-specific lipid transfer protein GPI-anchored 5 OS=Arabidopsis thaliana OX=3702 GN=LTPG5 PE=2 SV=1	PF14368	Probable lipid transfer	
XP_022021911.1	sp|Q9C9B1|LTG10_ARATH	40.82	LTG10_ARATH Non-specific lipid transfer protein GPI-anchored 10 OS=Arabidopsis thaliana OX=3702 GN=LTPG10 PE=3 SV=1	PF14368	Probable lipid transfer	
XP_022021912.1	sp|Q9C9B1|LTG10_ARATH	51.85	LTG10_ARATH Non-specific lipid transfer protein GPI-anchored 10 OS=Arabidopsis thaliana OX=3702 GN=LTPG10 PE=3 SV=1	PF14368	Probable lipid transfer	
XP_022021913.1	sp|Q9C9B1|LTG10_ARATH	51.9	LTG10_ARATH Non-specific lipid transfer protein GPI-anchored 10 OS=Arabidopsis thaliana OX=3702 GN=LTPG10 PE=3 SV=1	PF14368	Probable lipid transfer	
XP_022021914.1	sp|Q9C9B1|LTG10_ARATH	51.9	LTG10_ARATH Non-specific lipid transfer protein GPI-anchored 10 OS=Arabidopsis thaliana OX=3702 GN=LTPG10 PE=3 SV=1	PF14368	Probable lipid transfer	
XP_022029622.1	sp|Q8RW93|LBP65_ARATH	70	LBP65_ARATH Putative lipid-binding protein At4g00165 OS=Arabidopsis thaliana OX=3702 GN=At4g00165 PE=2 SV=1	PF14547	Hydrophobic seed protein	
XP_022035384.1	sp|O64864|LTG13_ARATH	51.79	LTG13_ARATH Non-specific lipid transfer protein GPI-anchored 13 OS=Arabidopsis thaliana OX=3702 GN=LTPG13 PE=2 SV=1	PF14368	Probable lipid transfer	
XP_022035551.1	sp|Q7EB72|LTG15_ARATH	40.44	LTG15_ARATH Non-specific lipid transfer protein GPI-anchored 15 OS=Arabidopsis thaliana OX=3702 GN=LTPG15 PE=2 SV=1	PF14368	Probable lipid transfer	
XP_022035552.1	sp|Q7EB72|LTG15_ARATH	39.34	LTG15_ARATH Non-specific lipid transfer protein GPI-anchored 15 OS=Arabidopsis thaliana OX=3702 GN=LTPG15 PE=2 SV=1	PF14368	Probable lipid transfer	
XP_035838717.1	sp|Q9LJ86|LTPG5_ARATH	57.04	LTPG5_ARATH Non-specific lipid transfer protein GPI-anchored 5 OS=Arabidopsis thaliana OX=3702 GN=LTPG5 PE=2 SV=1	PF14368	Probable lipid transfer	

Supplementary Table S4: Ratio between neutral mutation and purifying selection for each HaLTPs combination.In case of impossibility to estimate Kn/Ks, HaLTPs combination was not showed

HaLTPs comparison	Kn	Ks	Kn/Ks	Duplication events
XP_022002416.1 XP_022002417.1	0.0026	0.0105	0.24762	TANDEM
XP_022010295.2 XP_022013560.1	0.3241	0.6307	0.51387	TANDEM
XP_022010294.1 XP_022010296.1	0.3114	0.5356	0.5814	TANDEM
XP_022008275.1 XP_022008276.2	0.5533	0.9201	0.60135	TANDEM
XP_022002417.1 XP_022004455.1	0.2292	0.3775	0.60715	TANDEM
XP_022010296.1 XP_022013558.1	0.1424	0.2061	0.69093	TANDEM
XP_022002420.1 XP_022002421.1	0.2139	0.3074	0.69584	TANDEM
XP_021981395.1 XP_021981407.1	0.3141	0.4156	0.75577	TANDEM
XP_021993620.1 XP_021995116.1	0.8869	1.0795	0.82158	TANDEM
XP_022010292.1 XP_022013558.1	0.0748	0.0843	0.88731	TANDEM
XP_022033162.1 XP_022039074.2	0.3668	0.3914	0.93715	TANDEM
XP_022006926.1 XP_022006927.1	0.5174	0.5227	0.98986	TANDEM
XP_022009213.1 XP_035838717.1	0.0211	0.0202	1.04455	TANDEM
XP_021973854.1 XP_021973855.1	1.0329	0.9546	1.08202	TANDEM
XP_021989736.1 XP_021989737.1	0.3229	0.2972	1.08647	TANDEM
XP_021993220.1 XP_021993221.1	0.5966	0.4756	1.25442	TANDEM
XP_021989735.1 XP_021989736.1	0.1227	0.094	1.30532	TANDEM
XP_021987457.1 XP_021987458.1	0.017	0.0124	1.37097	TANDEM
XP_022013182.1 XP_022013183.1	0.0157	0.0114	1.37719	TANDEM
XP_022002415.1 XP_022002416.1	0.0286	0.0054	5.2963	TANDEM
XP_022010101.1 XP_022034230.1	0.7672	1.4877	0.5157	WGD
XP_022000196.1 XP_022022835.1	1.1307	1.6389	0.68991	WGD
XP_021984981.2 XP_021992525.1	0.8619	1.248	0.69063	WGD
XP_022002419.1 XP_022029738.1	0.4368	0.511	0.85479	WGD
XP_021981388.1 XP_022009213.1	0.6646	0.7521	0.88366	WGD
XP_022002421.1 XP_022029740.1	0.3881	0.401	0.96783	WGD
XP_021993622.1 XP_022013239.1	1.4256	1.462	0.9751	WGD
XP_021992525.1 XP_022035551.1	0.8803	0.8803	1	WGD
XP_021973854.1 XP_022006327.1	0.6847	0.6605	1.03664	WGD
XP_021989735.1 XP_022021914.1	1.2851	27.5526	0.04664	
XP_021993052.1 XP_021993620.1	1.4095	27.5526	0.05116	
XP_022029622.1 XP_035833070.1	1.6523	27.5526	0.05997	
XP_021993222.1 XP_022004980.1	1.6539	27.5526	0.06003	
XP_021981395.1 XP_022006327.1	1.6855	27.5526	0.06117	
XP_021968895.1 XP_022010616.1	0.8625	4.7228	0.18262	
XP_021989734.1 XP_022004979.1	1.0399	5.2195	0.19923	
XP_022010616.1 XP_022013182.1	1.0742	5.2692	0.20386	
XP_022006668.1 XP_022010101.1	1.0795	5.2195	0.20682	
XP_022022835.1 XP_022026964.1	1.1104	5.3452	0.20774	
XP_022010292.1 XP_035842399.1	0.9588	4.4973	0.21319	
XP_022010296.1 XP_022018622.1	1.0349	4.8235	0.21455	
XP_021984981.2 XP_022010294.1	1.1562	5.3524	0.21602	
XP_021981395.1 XP_021987457.1	1.2002	5.5412	0.2166	
XP_022010295.2 XP_022037775.1	1.0446	4.771	0.21895	
XP_022010292.1 XP_022035551.1	1.0569	4.6319	0.22818	
XP_022015084.1 XP_022029740.1	0.9477	4.072	0.23274	
XP_021981388.1 XP_022002416.1	0.9409	4.0088	0.23471	
XP_022002419.1 XP_022034230.1	0.8964	3.7903	0.2365	
XP_022002417.1 XP_035838717.1	0.9211	3.8821	0.23727	
XP_021984981.2 XP_022002420.1	1.0846	4.5461	0.23858	
XP_021986500.1 XP_022026963.1	1.1893	4.956	0.23997	
XP_021981388.1 XP_022002415.1	0.9477	3.8947	0.24333	
XP_022002420.1 XP_022009213.1	0.8848	3.6091	0.24516	
XP_021973011.1 XP_022034230.1	1.0431	4.2519	0.24533	
XP_022033162.1 XP_035835830.1	0.6355	2.5851	0.24583	
XP_021989736.1 XP_022010616.1	1.162	4.6889	0.24782	
XP_021986500.1 XP_022013558.1	0.9019	3.5843	0.25163	
XP_022002416.1 XP_035838717.1	0.8935	3.5353	0.25274	
XP_021989733.1 XP_021995117.1	0.9905	3.9106	0.25329	
XP_021981395.1 XP_022018622.1	1.1964	4.5869	0.26083	
XP_022008276.2 XP_022029738.1	1.0526	4.021	0.26178	
XP_022000196.1 XP_022010292.1	1.2054	4.5803	0.26317	
XP_021988229.1 XP_021989734.1	1.133	4.2951	0.26379	
XP_021986500.1 XP_021995117.1	1.0081	3.7845	0.26638	
XP_021993620.1 XP_022037775.1	1.3846	5.1846	0.26706	
XP_021981407.1 XP_022002416.1	0.9008	3.3704	0.26727	
XP_021993621.2 XP_022010294.1	1.0663	3.9547	0.26963	
XP_021993620.1 XP_022013560.1	0.9764	3.6212	0.26963	
XP_022004455.1 XP_022039074.2	0.6076	2.2342	0.27195	
XP_021987458.1 XP_022013560.1	1.028	3.7428	0.27466	
XP_022002415.1 XP_022037775.1	0.9996	3.6308	0.27531	
XP_021981407.1 XP_022002417.1	0.932	3.3665	0.27685	
XP_022008275.1 XP_022021911.1	1.096	3.9392	0.27823	
XP_021976022.1 XP_022006327.1	1.5694	5.6402	0.27825	
XP_021986500.1 XP_022013560.1	0.973	3.4905	0.27876	
XP_022002419.1 XP_022013183.1	1.1655	4.1618	0.28005	
XP_021981388.1 XP_022010292.1	1.0306	3.6636	0.28131	
XP_021973854.1 XP_022010101.1	1.1759	4.1381	0.28416	
XP_022002420.1 XP_022008275.1	1.0073	3.5321	0.28518	
XP_021969020.1 XP_022013183.1	1.2765	4.4727	0.2854	
XP_021981388.1 XP_022033162.1	1.1695	4.0947	0.28561	
XP_021992525.1 XP_022010616.1	1.1554	4.0377	0.28615	
XP_022004979.1 XP_022010295.2	0.8176	2.8444	0.28744	

XP_022004455.1	XP_022033162.1	0.6581	2.2834	0.28821
XP_021992525.1	XP_022002416.1	1.2388	4.2883	0.28888
XP_021981407.1	XP_022002415.1	0.9164	3.1576	0.29022
XP_021993222.1	XP_021995117.1	1.1244	3.8496	0.29208
XP_022000224.2	XP_022010294.1	0.7496	2.5637	0.29239
XP_022008275.1	XP_022021914.1	1.0652	3.6371	0.29287
XP_021989735.1	XP_035833070.1	1.3893	4.7365	0.29332
XP_021988953.1	XP_021993283.1	1.468	4.9973	0.29376
XP_021981388.1	XP_021986500.1	0.8435	2.8667	0.29424
XP_022029622.1	XP_022033162.1	1.312	4.4412	0.29542
XP_021981407.1	XP_022002418.1	0.9106	3.0812	0.29553
XP_021973316.1	XP_021981388.1	1.1336	3.8295	0.29602
XP_021981407.1	XP_022004455.1	0.9822	3.3105	0.29669
XP_022010294.1	XP_022037775.1	1.1753	3.9547	0.29719
XP_022002421.1	XP_022013183.1	1.2969	4.3615	0.29735
XP_021984981.2	XP_022002419.1	1.0133	3.4063	0.29748
XP_021989736.1	XP_022004455.1	0.8849	2.9678	0.29817
XP_021995117.1	XP_022033162.1	1.0367	3.4539	0.30015
XP_021981407.1	XP_022010919.1	1.1952	3.9719	0.30091
XP_021981395.1	XP_021985239.1	1.0094	3.3529	0.30105
XP_021995117.1	XP_022018622.1	1.167	3.8748	0.30118
XP_022010292.1	XP_035835830.1	0.6228	2.0637	0.30179
XP_022026964.1	XP_022035551.1	1.3869	4.5935	0.30193
XP_022026964.1	XP_022035552.1	1.3869	4.5935	0.30193
XP_021993222.1	XP_022001254.1	1.3304	4.3956	0.30267
XP_022004980.1	XP_022021912.1	1.302	4.2942	0.3032
XP_022013558.1	XP_035842399.1	0.9428	3.1014	0.30399
XP_021981395.1	XP_022002420.1	0.9782	3.2178	0.304
XP_021989736.1	XP_022000224.2	1.1209	3.6837	0.30429
XP_022005608.1	XP_022015084.1	1.1839	3.8821	0.30496
XP_021986500.1	XP_022010101.1	1.0835	3.5493	0.30527
XP_021993621.2	XP_022006927.1	0.9456	3.0971	0.30532
XP_021989734.1	XP_022002418.1	0.9704	3.1693	0.30619
XP_021969020.1	XP_022013182.1	1.2542	4.0857	0.30697
XP_022008275.1	XP_022029622.1	1.0918	3.5521	0.30737
XP_021984766.1	XP_022004455.1	1.035	3.3634	0.30772
XP_022002418.1	XP_022029622.1	0.8627	2.8033	0.30774
XP_022010616.1	XP_022013183.1	1.1746	3.8137	0.30799
XP_022002417.1	XP_022009213.1	0.9743	3.1436	0.30993
XP_022010616.1	XP_022013558.1	0.8364	2.6965	0.31018
XP_021973316.1	XP_021981626.1	1.4026	4.5161	0.31058
XP_022000224.2	XP_022013560.1	0.8184	2.6321	0.31093
XP_022002416.1	XP_022029622.1	0.8555	2.7444	0.31173
XP_022008275.1	XP_022021913.1	1.0733	3.4387	0.31212
XP_021981395.1	XP_022029622.1	1.1705	3.7462	0.31245
XP_021989735.1	XP_022021911.1	1.2581	4.0163	0.31325
XP_021981407.1	XP_021995117.1	1.0313	3.2893	0.31353
XP_021986500.1	XP_022002420.1	0.782	2.4902	0.31403
XP_021989733.1	XP_022010616.1	1.254	3.9864	0.31457
XP_021981407.1	XP_022015084.1	1.0642	3.3816	0.3147
XP_021993620.1	XP_022010294.1	1.066	3.3831	0.3151
XP_021989734.1	XP_021993620.1	1.1196	3.5431	0.31599
XP_022008276.2	XP_022010295.2	0.9891	3.1288	0.31613
XP_022010292.1	XP_022026963.1	1.0193	3.2178	0.31677
XP_022010292.1	XP_022034230.1	0.7774	2.4524	0.317
XP_022039074.2	XP_035835830.1	0.5955	1.868	0.31879
XP_022006927.1	XP_022033162.1	1.0171	3.1885	0.31899
XP_021972810.1	XP_022015084.1	1.0093	3.149	0.32051
XP_021981626.1	XP_021993621.2	1.5034	4.6802	0.32123
XP_021978807.1	XP_022013558.1	0.7649	2.378	0.32166
XP_021978807.1	XP_022010292.1	0.8266	2.5672	0.32199
XP_022014870.1	XP_022034230.1	1.1899	3.688	0.32264
XP_022000224.2	XP_035835830.1	0.8622	2.6704	0.32287
XP_021989737.1	XP_022015084.1	1.2016	3.7206	0.32296
XP_021993052.1	XP_022015084.1	1.0691	3.3069	0.32329
XP_022013558.1	XP_035838717.1	0.9983	3.0856	0.32354
XP_021989735.1	XP_022002416.1	0.9266	2.861	0.32387
XP_021981388.1	XP_022002418.1	0.9728	3.0025	0.324
XP_022001254.1	XP_022035384.1	1.1941	3.679	0.32457
XP_021976022.1	XP_021993551.1	1.6058	4.9438	0.32481
XP_022010616.1	XP_022033162.1	1.0831	3.332	0.32506
XP_022004979.1	XP_022010292.1	1.0267	3.1567	0.32524
XP_022013560.1	XP_022015084.1	0.9473	2.9059	0.32599
XP_021992525.1	XP_022002420.1	1.141	3.4966	0.32632
XP_022004979.1	XP_022006927.1	1.0649	3.2579	0.32687
XP_021984981.2	XP_021988229.1	0.9411	2.8719	0.32769
XP_021984981.2	XP_022002415.1	1.0407	3.1683	0.32847
XP_022018622.1	XP_022026963.1	1.2146	3.6972	0.32852
XP_022004455.1	XP_022006668.1	1.0009	3.0421	0.32902
XP_022002416.1	XP_022008275.1	1.0083	3.0531	0.33025
XP_022002417.1	XP_022008275.1	1.0083	3.0531	0.33025
XP_021981407.1	XP_035833070.1	1.3359	4.0447	0.33028
XP_022010292.1	XP_022033162.1	0.7216	2.1839	0.33042
XP_021968895.1	XP_022034230.1	1.0182	3.0708	0.33157
XP_021986500.1	XP_022008275.1	0.8572	2.5851	0.33159
XP_022013558.1	XP_035835830.1	0.6259	1.8873	0.33164
XP_021981407.1	XP_022013560.1	0.9615	2.8975	0.33184
XP_022001254.1	XP_022021913.1	1.2268	3.6856	0.33286

XP_022001254.1	XP_022021914.1	1.2268	3.6856	0.33286
XP_022010294.1	XP_022029622.1	1.1147	3.342	0.33354
XP_022010292.1	XP_022010616.1	0.8426	2.5255	0.33364
XP_022005608.1	XP_022033162.1	1.7246	5.1479	0.33501
XP_021986500.1	XP_022004455.1	0.8958	2.6726	0.33518
XP_022029739.1	XP_022039074.2	0.7767	2.3143	0.33561
XP_022002416.1	XP_022010101.1	0.7805	2.3177	0.33676
XP_022002418.1	XP_022010101.1	0.7691	2.2703	0.33877
XP_021981407.1	XP_021985239.1	0.9568	2.8236	0.33886
XP_022002420.1	XP_022039074.2	0.6159	1.8171	0.33895
XP_022001254.1	XP_022021912.1	1.2295	3.6164	0.33998
XP_021993621.2	XP_022039074.2	1.05	3.0832	0.34056
XP_021989733.1	XP_021993621.2	1.1161	3.2771	0.34058
XP_021989735.1	XP_022002418.1	0.9528	2.7913	0.34135
XP_022004455.1	XP_022026963.1	0.9207	2.6965	0.34144
XP_022010291.1	XP_022033162.1	0.7699	2.2457	0.34283
XP_021989734.1	XP_022000224.2	1.1629	3.3798	0.34407
XP_021984981.2	XP_022006668.1	1.4743	4.2778	0.34464
XP_021981388.1	XP_022004455.1	0.9642	2.7827	0.3465
XP_022004455.1	XP_022029622.1	0.8721	2.5076	0.34778
XP_022008276.2	XP_022013560.1	1.0824	3.1102	0.34802
XP_021988229.1	XP_022026964.1	1.1017	3.1609	0.34854
XP_021988229.1	XP_022035384.1	1.577	4.5205	0.34886
XP_021973011.1	XP_022004980.1	1.0457	2.9955	0.34909
XP_021973011.1	XP_021988953.1	1.3374	3.824	0.34974
XP_021978807.1	XP_022010296.1	0.8353	2.387	0.34994
XP_021988229.1	XP_022010296.1	0.8284	2.3575	0.35139
XP_022029622.1	XP_035835830.1	0.9195	2.6114	0.35211
XP_021995117.1	XP_022010292.1	0.773	2.1865	0.35353
XP_021981407.1	XP_022002420.1	0.9178	2.5875	0.35471
XP_022013239.1	XP_022015084.1	1.1658	3.2785	0.35559
XP_022013558.1	XP_022033162.1	0.7605	2.1322	0.35667
XP_021989736.1	XP_022021914.1	1.2542	3.5139	0.35693
XP_022010296.1	XP_022029739.1	0.5905	1.6541	0.35699
XP_021981395.1	XP_022022835.1	1.371	3.8393	0.3571
XP_022000196.1	XP_022006927.1	1.3827	3.865	0.35775
XP_021986500.1	XP_022015084.1	0.9886	2.762	0.35793
XP_021968895.1	XP_021993621.2	1.2435	3.4712	0.35823
XP_021973011.1	XP_021978842.1	1.0839	3.0254	0.35827
XP_022008276.2	XP_022033162.1	1.1367	3.1636	0.35931
XP_021978807.1	XP_022010294.1	0.8692	2.4096	0.36072
XP_021992525.1	XP_022008275.1	1.0576	2.9244	0.36165
XP_021981388.1	XP_022015084.1	1.0107	2.7943	0.3617
XP_021981395.1	XP_021993620.1	0.9817	2.711	0.36212
XP_021981395.1	XP_022008275.1	1.0128	2.7915	0.36282
XP_021988229.1	XP_022035551.1	1.1035	3.0413	0.36284
XP_021989735.1	XP_022002415.1	0.9549	2.6287	0.36326
XP_021981395.1	XP_021993220.1	1.242	3.4154	0.36365
XP_022029622.1	XP_022029738.1	1.0038	2.7527	0.36466
XP_022010296.1	XP_022029738.1	0.5383	1.4751	0.36492
XP_021989734.1	XP_021993551.1	1.2581	3.4463	0.36506
XP_021993621.2	XP_022010291.1	0.997	2.7222	0.36625
XP_021968895.1	XP_022010291.1	0.9139	2.4953	0.36625
XP_022008276.2	XP_022029739.1	1.1783	3.2143	0.36658
XP_022002415.1	XP_035838717.1	0.9103	2.4816	0.36682
XP_022008276.2	XP_022010292.1	1.0008	2.7267	0.36704
XP_021989735.1	XP_022004455.1	0.9235	2.5153	0.36715
XP_021973855.1	XP_035838717.1	1.6206	4.4133	0.36721
XP_021973854.1	XP_022026964.1	1.2857	3.4982	0.36753
XP_022006668.1	XP_035835830.1	0.8616	2.3322	0.36944
XP_022010295.2	XP_022026963.1	1.04	2.8074	0.37045
XP_022010292.1	XP_022029738.1	0.5436	1.4656	0.37091
XP_021995117.1	XP_022010296.1	0.8116	2.186	0.37127
XP_021993052.1	XP_035838717.1	1.1119	2.9917	0.37166
XP_022008275.1	XP_022010919.1	1.0101	2.7171	0.37176
XP_021973854.1	XP_022001254.1	1.4504	3.9004	0.37186
XP_022010292.1	XP_022029739.1	0.5699	1.5305	0.37236
XP_021981407.1	XP_021993222.1	1.2179	3.2701	0.37244
XP_021978807.1	XP_021995116.1	1.4153	3.7991	0.37254
XP_021978807.1	XP_021984981.2	0.9607	2.5777	0.3727
XP_021973316.1	XP_021986500.1	1.1645	3.1237	0.3728
XP_021981395.1	XP_022029740.1	1.1209	3.0066	0.37281
XP_021968895.1	XP_022006927.1	1.075	2.8782	0.3735
XP_021988229.1	XP_022010616.1	0.9389	2.5124	0.37371
XP_022006926.1	XP_022029622.1	1.2483	3.339	0.37385
XP_021989735.1	XP_021993621.2	1.1179	2.9871	0.37424
XP_022013182.1	XP_022022487.2	1.1656	3.1138	0.37433
XP_021981388.1	XP_022004980.1	1.0495	2.8017	0.37459
XP_021989737.1	XP_022006926.1	1.3133	3.4966	0.37559
XP_021981395.1	XP_022021914.1	1.1448	3.0431	0.3762
XP_021981626.1	XP_022026963.1	1.3222	3.5146	0.3762
XP_021989735.1	XP_022021912.1	1.2241	3.253	0.3763
XP_021988229.1	XP_021995116.1	1.2853	3.4138	0.3765
XP_021984981.2	XP_022000224.2	0.9487	2.5188	0.37665
XP_022008276.2	XP_022010294.1	0.9901	2.6284	0.37669
XP_022034230.1	XP_022035551.1	1.2509	3.3207	0.3767
XP_022034230.1	XP_022035552.1	1.2509	3.3207	0.3767
XP_021973854.1	XP_021985239.1	1.6373	4.3452	0.37681

XP_021989734.1	XP_022002417.1	0.9685	2.5658	0.37747
XP_022008275.1	XP_022034230.1	1.092	2.8905	0.37779
XP_022013558.1	XP_022029738.1	0.545	1.4418	0.378
XP_021988229.1	XP_022035552.1	1.1023	2.9159	0.37803
XP_021986500.1	XP_022018622.1	1.0749	2.8431	0.37807
XP_021981626.1	XP_022002419.1	0.9029	2.3876	0.37816
XP_021993052.1	XP_021995116.1	1.534	4.0554	0.37826
XP_022004455.1	XP_022006927.1	0.8304	2.1937	0.37854
XP_022034230.1	XP_035835830.1	0.8797	2.3214	0.37895
XP_022004979.1	XP_035833070.1	1.3726	3.6212	0.37905
XP_021989737.1	XP_022004455.1	0.972	2.5642	0.37907
XP_022004455.1	XP_022006327.1	1.2333	3.2486	0.37964
XP_021978842.1	XP_022026964.1	1.2124	3.1917	0.37986
XP_022015084.1	XP_022022835.1	1.129	2.9717	0.37992
XP_022008276.2	XP_022039085.1	1.306	3.4362	0.38007
XP_021992525.1	XP_021993621.2	1.2819	3.3634	0.38113
XP_021995116.1	XP_022002421.1	1.1709	3.072	0.38115
XP_021989733.1	XP_022010296.1	1.0658	2.7873	0.38238
XP_022010101.1	XP_022029738.1	0.8637	2.2587	0.38239
XP_021989734.1	XP_022002415.1	0.97	2.5364	0.38243
XP_022006927.1	XP_035835830.1	0.9885	2.5846	0.38246
XP_021973011.1	XP_021981388.1	0.895	2.3394	0.38258
XP_021999258.1	XP_022002415.1	1.0728	2.8001	0.38313
XP_021989736.1	XP_022021911.1	1.2276	3.1947	0.38426
XP_022002416.1	XP_022009213.1	0.9477	2.4659	0.38432
XP_021989735.1	XP_022029739.1	1.0828	2.8162	0.38449
XP_021981407.1	XP_022004980.1	1.0429	2.7122	0.38452
XP_021968895.1	XP_022037775.1	1.1432	2.9647	0.3856
XP_021989737.1	XP_022004980.1	1.0333	2.6774	0.38593
XP_022002415.1	XP_022010101.1	0.8047	2.0846	0.38602
XP_021978842.1	XP_022021913.1	1.3104	3.3941	0.38608
XP_021978842.1	XP_022021914.1	1.3104	3.3941	0.38608
XP_021985239.1	XP_035835830.1	0.7755	2.0083	0.38615
XP_022021911.1	XP_022029622.1	1.3916	3.603	0.38623
XP_021993620.1	XP_021995117.1	1.0691	2.7629	0.38695
XP_022008275.1	XP_022029738.1	1.0196	2.6341	0.38708
XP_022002421.1	XP_035833070.1	1.1299	2.9101	0.38827
XP_021993220.1	XP_022029740.1	0.9737	2.5077	0.38828
XP_021978807.1	XP_021993220.1	1.2031	3.0978	0.38837
XP_022013558.1	XP_022029739.1	0.5694	1.4627	0.38928
XP_021988953.1	XP_035842399.1	1.5051	3.865	0.38942
XP_022010101.1	XP_022010295.2	1.0246	2.6292	0.3897
XP_022002419.1	XP_022029622.1	0.9286	2.382	0.38984
XP_021989736.1	XP_021993620.1	1.1162	2.8619	0.39002
XP_021989734.1	XP_022010296.1	1.0359	2.6557	0.39007
XP_022000224.2	XP_022039085.1	0.772	1.9778	0.39033
XP_021969020.1	XP_022014870.1	1.3027	3.332	0.39097
XP_022010101.1	XP_022010296.1	0.9122	2.3318	0.3912
XP_022002420.1	XP_022008276.2	1.0712	2.731	0.39224
XP_022013560.1	XP_035838717.1	1.0779	2.7412	0.39322
XP_021985239.1	XP_022029739.1	0.8695	2.2105	0.39335
XP_022002420.1	XP_035835830.1	0.4514	1.1475	0.39338
XP_021981395.1	XP_021993621.2	0.8845	2.2478	0.3935
XP_022002419.1	XP_022039074.2	0.6185	1.5716	0.39355
XP_021989734.1	XP_021993621.2	1.1252	2.855	0.39412
XP_021993621.2	XP_022013560.1	1.0857	2.7531	0.39436
XP_021984981.2	XP_022006927.1	1.3301	3.3686	0.39485
XP_021986500.1	XP_022021911.1	0.809	2.0485	0.39492
XP_021986500.1	XP_022021913.1	0.809	2.0485	0.39492
XP_021986500.1	XP_022021914.1	0.809	2.0485	0.39492
XP_021988953.1	XP_022000196.1	1.178	2.9818	0.39506
XP_021981395.1	XP_022021913.1	1.1476	2.9047	0.39508
XP_021988229.1	XP_022037775.1	1.3791	3.4905	0.3951
XP_022002421.1	XP_022039074.2	0.7149	1.8061	0.39583
XP_021969020.1	XP_022002420.1	0.8828	2.2301	0.39586
XP_021987457.1	XP_022004455.1	0.9986	2.518	0.39658
XP_021988229.1	XP_021989735.1	1.1218	2.8284	0.39662
XP_021981626.1	XP_022021912.1	1.3849	3.4905	0.39676
XP_021987458.1	XP_022004455.1	1.016	2.5584	0.39712
XP_021989733.1	XP_021993620.1	1.0633	2.675	0.3975
XP_022014870.1	XP_022029740.1	1.0735	2.6965	0.39811
XP_021973011.1	XP_022037775.1	1.3992	3.5135	0.39824
XP_021973855.1	XP_022008275.1	1.3354	3.3494	0.3987
XP_022010101.1	XP_022035552.1	1.2566	3.1515	0.39873
XP_022010296.1	XP_022033162.1	0.8619	2.1604	0.39895
XP_022013558.1	XP_022018622.1	0.9628	2.4115	0.39925
XP_021989735.1	XP_022010292.1	0.9895	2.475	0.3998
XP_021981407.1	XP_021986500.1	0.9185	2.2952	0.40018
XP_021993621.2	XP_021995117.1	1.1952	2.9843	0.4005
XP_022010101.1	XP_022039074.2	0.925	2.3093	0.40055
XP_021973011.1	XP_022026963.1	1.2888	3.213	0.40112
XP_022010101.1	XP_035835830.1	0.8089	2.0146	0.40152
XP_021981388.1	XP_021988229.1	1.2339	3.0708	0.40182
XP_021989734.1	XP_022021912.1	1.3709	3.4096	0.40207
XP_022002415.1	XP_022008275.1	1.0203	2.5357	0.40237
XP_021969020.1	XP_021993622.1	1.5322	3.8057	0.40261
XP_022013182.1	XP_022029740.1	1.3223	3.279	0.40326
XP_021981626.1	XP_021985239.1	1.1181	2.7714	0.40344

XP_021973854.1	XP_022004455.1	1.1945	2.9594	0.40363
XP_021981395.1	XP_022004979.1	0.9386	2.3251	0.40368
XP_021972810.1	XP_021993220.1	1.112	2.7505	0.40429
XP_021969020.1	XP_022002415.1	0.9161	2.2657	0.40433
XP_021993620.1	XP_022013558.1	0.9895	2.4451	0.40469
XP_022002417.1	XP_022039074.2	0.6401	1.5816	0.40472
XP_021969020.1	XP_022002416.1	0.9509	2.3473	0.4051
XP_022013183.1	XP_022022487.2	1.2389	3.0518	0.40596
XP_021989737.1	XP_022010294.1	0.9222	2.271	0.40608
XP_022015084.1	XP_022021912.1	1.2996	3.194	0.40689
XP_022000196.1	XP_022004980.1	1.234	3.0323	0.40695
XP_021993620.1	XP_022010296.1	0.9882	2.4267	0.40722
XP_021993221.1	XP_022002419.1	0.9664	2.3712	0.40756
XP_021989733.1	XP_022006926.1	1.378	3.3811	0.40756
XP_021987458.1	XP_021988953.1	1.0741	2.6349	0.40764
XP_022015084.1	XP_035838717.1	1.0738	2.6328	0.40785
XP_021989735.1	XP_022002417.1	0.9608	2.3554	0.40791
XP_022004979.1	XP_022029740.1	0.8622	2.1128	0.40808
XP_021978807.1	XP_021981395.1	1.1355	2.7785	0.40867
XP_022033162.1	XP_022037775.1	1.2305	3.0104	0.40875
XP_022008276.2	XP_022018622.1	1.4422	3.5263	0.40898
XP_021989734.1	XP_022010101.1	1.0278	2.5117	0.4092
XP_021995117.1	XP_022013560.1	0.878	2.1445	0.40942
XP_022010295.2	XP_022014870.1	0.8367	2.0421	0.40973
XP_022013182.1	XP_022026963.1	1.2235	2.984	0.41002
XP_021972810.1	XP_021973854.1	1.074	2.6163	0.4105
XP_021989733.1	XP_022010101.1	1.1357	2.7657	0.41064
XP_022013560.1	XP_022021911.1	1.3616	3.3152	0.41071
XP_022013560.1	XP_022021913.1	1.3616	3.3152	0.41071
XP_022013560.1	XP_022021914.1	1.3616	3.3152	0.41071
XP_022002415.1	XP_022029622.1	0.9012	2.1937	0.41081
XP_021993222.1	XP_022010296.1	1.3449	3.2721	0.41102
XP_022035551.1	XP_035833070.1	1.5942	3.8764	0.41126
XP_022009213.1	XP_022013560.1	1.1238	2.7292	0.41177
XP_021985239.1	XP_022002417.1	0.7633	1.8537	0.41177
XP_022006327.1	XP_022006926.1	1.2261	2.9714	0.41263
XP_021992525.1	XP_022002418.1	1.2827	3.1074	0.41279
XP_021984981.2	XP_022002418.1	1.0891	2.6366	0.41307
XP_022002421.1	XP_022014870.1	1.043	2.5233	0.41335
XP_021993620.1	XP_035833070.1	0.8715	2.1061	0.4138
XP_022010294.1	XP_022034230.1	0.9485	2.2899	0.41421
XP_021978807.1	XP_022010291.1	0.9366	2.2611	0.41422
XP_022029739.1	XP_035838717.1	1.0187	2.4576	0.41451
XP_022002421.1	XP_035838717.1	1.0236	2.469	0.41458
XP_021989735.1	XP_022002420.1	0.8934	2.1541	0.41474
XP_021989735.1	XP_022002419.1	0.877	2.1107	0.4155
XP_021981388.1	XP_022018622.1	1.1556	2.7812	0.4155
XP_022010101.1	XP_022035551.1	1.2627	3.0383	0.41559
XP_021986500.1	XP_022021912.1	0.75	1.802	0.4162
XP_022000196.1	XP_022013558.1	1.1684	2.8068	0.41627
XP_022004980.1	XP_022006926.1	1.5274	3.6655	0.4167
XP_022000196.1	XP_022010295.2	1.0041	2.4096	0.41671
XP_021981407.1	XP_022013558.1	0.9154	2.1963	0.41679
XP_022004979.1	XP_022008276.2	1.2189	2.9232	0.41697
XP_022008276.2	XP_022010291.1	1.0618	2.544	0.41737
XP_022029740.1	XP_022037775.1	1.0835	2.5953	0.41749
XP_021981407.1	XP_022000224.2	1.1165	2.6713	0.41796
XP_021973855.1	XP_022026964.1	1.606	3.8372	0.41853
XP_022001254.1	XP_022021911.1	1.2133	2.8967	0.41886
XP_021984981.2	XP_021993621.2	1.0925	2.6026	0.41977
XP_022035552.1	XP_035833070.1	1.6241	3.865	0.42021
XP_021989735.1	XP_022004980.1	0.9454	2.2478	0.42059
XP_021989736.1	XP_022021912.1	1.1943	2.8357	0.42117
XP_021993620.1	XP_022010292.1	1.0191	2.4197	0.42117
XP_021989736.1	XP_022018622.1	1.2445	2.9517	0.42162
XP_021988953.1	XP_022002419.1	1.0973	2.6014	0.42181
XP_022021913.1	XP_022029622.1	1.399	3.3141	0.42214
XP_022021914.1	XP_022029622.1	1.399	3.3141	0.42214
XP_022002419.1	XP_035838717.1	0.9371	2.2195	0.42221
XP_021988229.1	XP_022002421.1	0.766	1.8116	0.42283
XP_022002417.1	XP_022035551.1	0.9446	2.2322	0.42317
XP_022002417.1	XP_022035552.1	0.9446	2.2322	0.42317
XP_021972810.1	XP_021984766.1	1.2482	2.9489	0.42328
XP_021989736.1	XP_022002416.1	0.8636	2.0401	0.42331
XP_022002419.1	XP_022004980.1	1.1478	2.7105	0.42346
XP_021992525.1	XP_022004455.1	1.0392	2.4531	0.42363
XP_021993620.1	XP_022034230.1	1.4138	3.332	0.42431
XP_022002420.1	XP_022014870.1	1.1201	2.637	0.42476
XP_021973316.1	XP_022029622.1	1.1889	2.7984	0.42485
XP_021986500.1	XP_035835830.1	0.8199	1.9237	0.42621
XP_021993620.1	XP_022033162.1	1.144	2.684	0.42623
XP_022002416.1	XP_022039074.2	0.6391	1.4988	0.42641
XP_021993620.1	XP_022029739.1	1.1393	2.6718	0.42642
XP_022010294.1	XP_035835830.1	0.7413	1.7384	0.42643
XP_022010295.2	XP_022033162.1	0.8451	1.9817	0.42645
XP_021987457.1	XP_022026963.1	1.3813	3.2381	0.42658
XP_021968895.1	XP_022006926.1	0.961	2.2526	0.42662
XP_021985239.1	XP_022035384.1	1.4762	3.4596	0.4267

XP_021978807.1	XP_022029622.1	1.2054	2.8241	0.42683
XP_021986500.1	XP_021993620.1	1.3661	3.1954	0.42752
XP_021989737.1	XP_022029739.1	1.1036	2.5783	0.42803
XP_021978842.1	XP_021987458.1	1.2468	2.9127	0.42806
XP_022002415.1	XP_022039074.2	0.6295	1.4706	0.42806
XP_022002417.1	XP_022034230.1	0.9308	2.1737	0.42821
XP_021989736.1	XP_022002418.1	0.8832	2.0617	0.42838
XP_022010101.1	XP_022029740.1	0.8926	2.0773	0.42969
XP_022002420.1	XP_022033162.1	0.6632	1.5432	0.42976
XP_022029739.1	XP_035833070.1	1.3004	3.0257	0.42978
XP_021973011.1	XP_021993620.1	1.2135	2.817	0.43078
XP_022002418.1	XP_022008275.1	1.0257	2.381	0.43079
XP_021981388.1	XP_021985239.1	0.954	2.2144	0.43082
XP_021989736.1	XP_022033162.1	1.1328	2.6288	0.43092
XP_021968895.1	XP_022035384.1	1.3394	3.1045	0.43144
XP_021993620.1	XP_022014870.1	1.2953	3	0.43177
XP_022013558.1	XP_022034230.1	0.8106	1.8774	0.43177
XP_021989736.1	XP_022002415.1	0.8812	2.0405	0.43185
XP_022002419.1	XP_022006668.1	1.0056	2.328	0.43196
XP_022006668.1	XP_022008276.2	1.1922	2.7597	0.432
XP_022035551.1	XP_035842399.1	1.4644	3.3894	0.43205
XP_022002420.1	XP_022006668.1	0.9442	2.1798	0.43316
XP_022002421.1	XP_022010101.1	0.926	2.1351	0.4337
XP_021968895.1	XP_022015084.1	1.0937	2.5194	0.43411
XP_021984981.2	XP_022002416.1	1.0799	2.4862	0.43436
XP_021984981.2	XP_022010296.1	1.2186	2.8015	0.43498
XP_022005608.1	XP_022010616.1	1.1625	2.6704	0.43533
XP_022004979.1	XP_035835830.1	1.0391	2.3835	0.43596
XP_022015084.1	XP_022033162.1	1.0695	2.4531	0.43598
XP_021986500.1	XP_022010292.1	0.9114	2.0888	0.43633
XP_021981407.1	XP_022029739.1	1.1699	2.6794	0.43663
XP_021989733.1	XP_021993551.1	1.1937	2.7334	0.43671
XP_022010919.1	XP_035842399.1	1.8284	4.1832	0.43708
XP_021989736.1	XP_022002419.1	0.9224	2.1101	0.43714
XP_021989737.1	XP_021995117.1	0.999	2.2834	0.43751
XP_021993052.1	XP_022035384.1	1.1796	2.6939	0.43788
XP_021981626.1	XP_022021913.1	1.4592	3.3306	0.43812
XP_021987457.1	XP_022014870.1	1.2124	2.7657	0.43837
XP_021987458.1	XP_022014870.1	1.2124	2.7657	0.43837
XP_022010294.1	XP_022010616.1	0.8852	2.0183	0.43859
XP_021968895.1	XP_022018622.1	1.0811	2.4646	0.43865
XP_022002417.1	XP_022035384.1	1.278	2.9133	0.43868
XP_021978807.1	XP_022010616.1	0.8179	1.8637	0.43886
XP_022002420.1	XP_022004455.1	0.1885	0.4294	0.43898
XP_022009213.1	XP_035835830.1	0.9456	2.1538	0.43904
XP_021969020.1	XP_021978807.1	1.1676	2.6591	0.4391
XP_021984981.2	XP_022029740.1	1.1288	2.5698	0.43926
XP_021972810.1	XP_022004979.1	1.0807	2.4589	0.43951
XP_021981626.1	XP_022002420.1	0.9404	2.1384	0.43977
XP_022035384.1	XP_035842399.1	1.6526	3.7563	0.43995
XP_022013558.1	XP_022014870.1	0.7876	1.7891	0.44022
XP_021968895.1	XP_021973854.1	1.2816	2.9082	0.44068
XP_021993222.1	XP_022029738.1	1.1218	2.5454	0.44072
XP_021972810.1	XP_022013183.1	1.4105	3.2	0.44078
XP_021986500.1	XP_022009213.1	0.8648	1.9612	0.44095
XP_021986500.1	XP_035838717.1	0.8648	1.9612	0.44095
XP_022009213.1	XP_022013558.1	1.0392	2.3563	0.44103
XP_022008276.2	XP_022021912.1	1.2916	2.9282	0.44109
XP_022002418.1	XP_022039074.2	0.6198	1.4048	0.4412
XP_022006927.1	XP_035833070.1	1.3315	3.0085	0.44258
XP_021978807.1	XP_021986500.1	0.9726	2.1947	0.44316
XP_021989734.1	XP_021995117.1	0.9685	2.1854	0.44317
XP_022010295.2	XP_022029738.1	0.5897	1.3306	0.44318
XP_021973011.1	XP_021995116.1	1.2702	2.866	0.4432
XP_022037775.1	XP_022039085.1	1.3049	2.9421	0.44353
XP_022018622.1	XP_022039074.2	1.1264	2.5383	0.44376
XP_021989736.1	XP_022029739.1	1.0622	2.3936	0.44377
XP_022000224.2	XP_022010291.1	0.8655	1.9486	0.44417
XP_022010296.1	XP_022034230.1	0.9408	2.1181	0.44417
XP_021989735.1	XP_022013558.1	0.962	2.1644	0.44446
XP_021995116.1	XP_022021912.1	1.7453	3.9265	0.44449
XP_021978807.1	XP_022005608.1	0.9828	2.2098	0.44475
XP_022002419.1	XP_022009213.1	0.9857	2.2162	0.44477
XP_021987457.1	XP_021988953.1	1.0903	2.4495	0.44511
XP_022002419.1	XP_022033162.1	0.6161	1.3822	0.44574
XP_022000196.1	XP_022021912.1	1.1796	2.6443	0.44609
XP_021973011.1	XP_035838717.1	1.03	2.3089	0.4461
XP_021969020.1	XP_035835830.1	1.0033	2.2488	0.44615
XP_021993621.2	XP_022004979.1	1.2634	2.8296	0.44649
XP_021989733.1	XP_022002419.1	0.9144	2.0463	0.44686
XP_021968895.1	XP_021986500.1	0.9285	2.0776	0.44691
XP_021981388.1	XP_021987458.1	1.1336	2.5357	0.44706
XP_021989733.1	XP_022010294.1	0.9904	2.2149	0.44715
XP_021978842.1	XP_022010616.1	1.0055	2.2483	0.44723
XP_021993620.1	XP_022010616.1	1.3251	2.9627	0.44726
XP_021989737.1	XP_022008275.1	1.0799	2.4142	0.44731
XP_021981388.1	XP_021987457.1	1.0868	2.4296	0.44732
XP_021981407.1	XP_022010295.2	1.0515	2.3466	0.4481

XP_022004455.1	XP_022022835.1	1.0364	2.3129	0.4481
XP_021999258.1	XP_022000196.1	1.2098	2.6986	0.44831
XP_022000224.2	XP_022013183.1	1.1232	2.5045	0.44847
XP_022022487.2	XP_035838717.1	1.2299	2.7412	0.44867
XP_022002420.1	XP_022035551.1	0.9228	2.0524	0.44962
XP_022002420.1	XP_022035552.1	0.9228	2.0524	0.44962
XP_021995117.1	XP_022013558.1	0.752	1.6712	0.44998
XP_022033162.1	XP_035838717.1	1.1046	2.4547	0.44999
XP_022004980.1	XP_022026963.1	1.332	2.9586	0.45021
XP_022002421.1	XP_022029622.1	0.9602	2.1326	0.45025
XP_021978842.1	XP_022021911.1	1.288	2.8605	0.45027
XP_022008275.1	XP_022013239.1	1.1396	2.5305	0.45035
XP_021993551.1	XP_022008275.1	1.6091	3.5716	0.45053
XP_022006926.1	XP_035835830.1	0.9817	2.1773	0.45088
XP_022026964.1	XP_035833070.1	1.397	3.0971	0.45107
XP_022004455.1	XP_022039085.1	0.5914	1.3095	0.45162
XP_021981388.1	XP_022002420.1	0.9557	2.1148	0.45191
XP_021973011.1	XP_022006926.1	1.1474	2.538	0.45209
XP_021972810.1	XP_022010101.1	0.8325	1.8397	0.45252
XP_021989735.1	XP_021995117.1	1.0272	2.2699	0.45253
XP_021972810.1	XP_022008276.2	1.1138	2.4607	0.45264
XP_022002418.1	XP_035838717.1	0.9518	2.1025	0.4527
XP_021988229.1	XP_022015084.1	1.1385	2.5136	0.45294
XP_021988953.1	XP_022022835.1	1.2815	2.829	0.45299
XP_021972810.1	XP_022006927.1	0.9679	2.1348	0.45339
XP_021993221.1	XP_022002417.1	0.9958	2.1955	0.45356
XP_021995116.1	XP_022010296.1	1.0246	2.2588	0.4536
XP_021981626.1	XP_021993551.1	1.6635	3.664	0.45401
XP_021969020.1	XP_022037775.1	1.2693	2.7956	0.45403
XP_022013183.1	XP_022026963.1	1.2492	2.7508	0.45412
XP_022002416.1	XP_022035384.1	1.2679	2.7908	0.45431
XP_022035551.1	XP_035835830.1	1.0779	2.37	0.45481
XP_022035552.1	XP_035835830.1	1.0779	2.37	0.45481
XP_021993052.1	XP_022004980.1	1.2993	2.855	0.4551
XP_021972810.1	XP_022001254.1	1.0996	2.4161	0.45511
XP_021968895.1	XP_022010101.1	0.9807	2.1516	0.4558
XP_022000224.2	XP_022013182.1	1.0167	2.2301	0.4559
XP_021989734.1	XP_022033162.1	1.1747	2.5766	0.45591
XP_021989734.1	XP_022010294.1	0.972	2.1314	0.45604
XP_021985239.1	XP_021999258.1	1.1172	2.4477	0.45643
XP_021985239.1	XP_022002416.1	0.7614	1.6681	0.45645
XP_021993620.1	XP_022039085.1	1.1941	2.6143	0.45676
XP_022002416.1	XP_022006668.1	1.0163	2.224	0.45697
XP_022010919.1	XP_022035551.1	1.0966	2.396	0.45768
XP_022010919.1	XP_022035552.1	1.0966	2.396	0.45768
XP_022004980.1	XP_022014870.1	1.2627	2.7512	0.45896
XP_022002416.1	XP_022035551.1	0.9463	2.0578	0.45986
XP_022002416.1	XP_022035552.1	0.9463	2.0578	0.45986
XP_021976022.1	XP_022010296.1	0.9586	2.084	0.45998
XP_021988229.1	XP_021989736.1	1.1441	2.4869	0.46005
XP_021993622.1	XP_022033162.1	1.2661	2.7512	0.4602
XP_022002419.1	XP_022014870.1	1.0202	2.2165	0.46028
XP_021973316.1	XP_022035552.1	1.351	2.9329	0.46064
XP_022010296.1	XP_022014870.1	0.9361	2.031	0.46091
XP_021989736.1	XP_021993551.1	1.2717	2.7553	0.46155
XP_021987458.1	XP_022039074.2	1.1784	2.5523	0.4617
XP_022018622.1	XP_022037775.1	1.1222	2.429	0.462
XP_022015084.1	XP_022039074.2	1.055	2.2834	0.46203
XP_021978842.1	XP_022001254.1	1.1224	2.429	0.46208
XP_022029622.1	XP_022039085.1	1.0178	2.2	0.46264
XP_021972810.1	XP_022010294.1	0.7966	1.7207	0.46295
XP_021985239.1	XP_022002419.1	0.7687	1.6595	0.46321
XP_021993620.1	XP_022006927.1	1.2891	2.7827	0.46326
XP_021973854.1	XP_022039074.2	1.3836	2.985	0.46352
XP_021993622.1	XP_021995116.1	1.3334	2.8758	0.46366
XP_022002418.1	XP_022035551.1	1.0011	2.159	0.46369
XP_022002418.1	XP_022035552.1	1.0011	2.159	0.46369
XP_021978842.1	XP_022015084.1	1.0678	2.3026	0.46374
XP_021981407.1	XP_022039085.1	1.291	2.7834	0.46382
XP_022006927.1	XP_022010292.1	0.9188	1.9808	0.46385
XP_022010292.1	XP_022018622.1	0.974	2.0996	0.4639
XP_021989733.1	XP_022000224.2	1.212	2.6126	0.46391
XP_022002415.1	XP_035833070.1	1.2071	2.6011	0.46407
XP_021988229.1	XP_022004455.1	0.6951	1.4971	0.4643
XP_021989736.1	XP_022002417.1	0.8734	1.8811	0.4643
XP_022002417.1	XP_022006668.1	1.0146	2.1815	0.46509
XP_021969020.1	XP_022010291.1	1.2198	2.6224	0.46515
XP_022002417.1	XP_022006927.1	0.9173	1.9719	0.46519
XP_021988229.1	XP_022010295.2	0.834	1.7927	0.46522
XP_021989733.1	XP_035842399.1	1.3899	2.9858	0.4655
XP_022010616.1	XP_022013560.1	0.8844	1.8995	0.4656
XP_021981626.1	XP_022010101.1	1.2589	2.7024	0.46585
XP_022004980.1	XP_022005608.1	1.2424	2.6651	0.46617
XP_022002419.1	XP_022021912.1	1.057	2.2667	0.46632
XP_022010101.1	XP_022035384.1	1.2925	2.7716	0.46634
XP_022035552.1	XP_035842399.1	1.4577	3.1229	0.46678
XP_021989736.1	XP_035835830.1	0.9978	2.137	0.46692
XP_021981388.1	XP_022008275.1	0.9857	2.1103	0.46709

XP_022006927.1	XP_022013558.1		0.8752	1.8683	0.46845
XP_021995117.1	XP_022010616.1		0.9697	2.07	0.46845
XP_021989737.1	XP_022006668.1		1.2977	2.7604	0.47011
XP_022013560.1	XP_022021912.1		1.3926	2.9614	0.47025
XP_021969020.1	XP_022029622.1		1.5716	3.342	0.47026
XP_021987457.1	XP_022009213.1		1.0004	2.1265	0.47044
XP_021993621.2	XP_022009213.1		1.1705	2.4869	0.47067
XP_021985239.1	XP_022002420.1		0.7827	1.6615	0.47108
XP_022000196.1	XP_022009213.1		1.3136	2.7852	0.47164
XP_022010101.1	XP_022013560.1		0.839	1.7777	0.47196
XP_021973316.1	XP_022035551.1		1.3546	2.8688	0.47218
XP_021988229.1	XP_022010292.1		0.8247	1.7458	0.47239
XP_021999258.1	XP_022006327.1		1.2576	2.6551	0.47365
XP_022008275.1	XP_022035384.1		1.3381	2.8248	0.4737
XP_022001254.1	XP_035838717.1		1.3177	2.7806	0.47389
XP_022010294.1	XP_022014870.1		1.0792	2.2758	0.47421
XP_021968895.1	XP_022004980.1		1.3373	2.8177	0.47461
XP_022010295.2	XP_022029739.1		0.6526	1.3744	0.47483
XP_021989737.1	XP_021993052.1		1.0784	2.2699	0.47509
XP_021981395.1	XP_022029739.1		1.1318	2.382	0.47515
XP_021993620.1	XP_022013183.1		1.1059	2.3265	0.47535
XP_021989736.1	XP_021993621.2		1.1179	2.3516	0.47538
XP_021972810.1	XP_022026963.1		1.3587	2.855	0.4759
XP_021995117.1	XP_022029739.1		1.0342	2.1713	0.4763
XP_021976022.1	XP_022004455.1		1.1027	2.3134	0.47666
XP_022001254.1	XP_022004980.1		1.2504	2.6224	0.47682
XP_021981395.1	XP_022021911.1		1.1715	2.4556	0.47707
XP_021995117.1	XP_022010295.2		0.7456	1.5627	0.47712
XP_022002420.1	XP_022010101.1		0.8267	1.7319	0.47734
XP_021968895.1	XP_021989733.1		1.1367	2.3803	0.47754
XP_021995116.1	XP_021999258.1		1.3175	2.7586	0.4776
XP_021989734.1	XP_022006926.1		1.2836	2.6876	0.4776
XP_021989737.1	XP_022026963.1		1.5181	3.1756	0.47805
XP_021978807.1	XP_021995117.1		0.8945	1.8709	0.47811
XP_021986500.1	XP_022002421.1		0.8176	1.7094	0.4783
XP_021985239.1	XP_022022487.2		0.9548	1.9961	0.47833
XP_022000224.2	XP_022013558.1		0.8085	1.6897	0.47849
XP_021972810.1	XP_022004980.1		1.3135	2.7442	0.47865
XP_021989735.1	XP_021993620.1		1.104	2.3045	0.47906
XP_022013182.1	XP_022039085.1		1.5829	3.3007	0.47956
XP_022000224.2	XP_022006327.1		1.3988	2.9156	0.47976
XP_021986500.1	XP_022002416.1		0.7796	1.6243	0.47996
XP_021987458.1	XP_022006327.1		1.4052	2.9271	0.48007
XP_021989734.1	XP_035842399.1		1.4873	3.0976	0.48015
XP_021989737.1	XP_035833070.1		1.313	2.7341	0.48023
XP_021993222.1	XP_022037775.1		1.4212	2.9594	0.48023
XP_022004455.1	XP_022021912.1		0.9441	1.9643	0.48063
XP_022000196.1	XP_022033162.1		1.2142	2.5255	0.48078
XP_022002416.1	XP_022034230.1		0.9342	1.9417	0.48112
XP_021993222.1	XP_022010291.1		1.1651	2.4215	0.48115
XP_022013183.1	XP_022029740.1		1.3898	2.8838	0.48193
XP_021968895.1	XP_022002420.1		0.7277	1.508	0.48256
XP_021968895.1	XP_035838717.1		1.2782	2.6485	0.48261
XP_021995117.1	XP_022008276.2		1.0436	2.1623	0.48263
XP_022001254.1	XP_022010101.1		1.3638	2.8209	0.48346
XP_022000196.1	XP_022004979.1		1.1466	2.3715	0.48349
XP_022010101.1	XP_022039085.1		0.9794	2.0256	0.48351
XP_022013560.1	XP_035842399.1		1.0755	2.2218	0.48407
XP_021984766.1	XP_022014870.1		1.0804	2.2306	0.48435
XP_021988953.1	XP_022004979.1		1.1862	2.4485	0.48446
XP_022018622.1	XP_022026964.1		1.396	2.8806	0.48462
XP_021993620.1	XP_022008276.2		1.2111	2.4987	0.48469
XP_022013182.1	XP_022034230.1		1.1072	2.2834	0.48489
XP_021978842.1	XP_021987457.1		1.2401	2.5571	0.48496
XP_021981395.1	XP_021995116.1		1.3016	2.6833	0.48507
XP_022008275.1	XP_022039085.1		1.2015	2.4764	0.48518
XP_021984981.2	XP_022029739.1		1.0526	2.1695	0.48518
XP_022002416.1	XP_022010294.1		0.5475	1.1281	0.48533
XP_021989733.1	XP_022033162.1		1.1216	2.3105	0.48544
XP_021984981.2	XP_022002417.1		1.112	2.2904	0.4855
XP_022010101.1	XP_022010291.1		0.9106	1.8738	0.48596
XP_021993621.2	XP_022008276.2		1.1863	2.4404	0.48611
XP_022029738.1	XP_035833070.1		1.275	2.6224	0.4862
XP_022029739.1	XP_035835830.1		0.5979	1.2297	0.48622
XP_022001254.1	XP_022005608.1		1.1147	2.2905	0.48666
XP_021981388.1	XP_022029622.1		1.1144	2.2894	0.48677
XP_022013558.1	XP_022039085.1		0.6722	1.3809	0.48678
XP_021995116.1	XP_022002418.1		1.1588	2.3793	0.48703
XP_022010294.1	XP_022010295.2		0.3203	0.6575	0.48715
XP_021973855.1	XP_021981388.1		1.6511	3.3886	0.48725
XP_021973011.1	XP_022010291.1		0.8562	1.7572	0.48725
XP_021987457.1	XP_022013560.1		1.052	2.1586	0.48735
XP_021978807.1	XP_022008275.1		1.0721	2.1978	0.48781
XP_021993551.1	XP_022029622.1		1.378	2.8246	0.48786
XP_022022487.2	XP_022035551.1		1.2342	2.5296	0.4879
XP_021986500.1	XP_022010294.1		0.9475	1.9409	0.48818
XP_021984766.1	XP_022000196.1		1.2233	2.5045	0.48844
XP_022014870.1	XP_022022487.2		1.3878	2.84	0.48866

XP_022006927.1	XP_022026964.1		1.1118	2.2751	0.48868
XP_022002419.1	XP_022006927.1		0.9282	1.8964	0.48945
XP_021981626.1	XP_022029740.1		1.1977	2.4454	0.48978
XP_022033162.1	XP_022035384.1		1.4015	2.861	0.48986
XP_021999258.1	XP_022010616.1		1.4703	2.9983	0.49038
XP_022010294.1	XP_022035384.1		1.1704	2.3866	0.4904
XP_021978842.1	XP_021989736.1		0.937	1.9098	0.49063
XP_021986500.1	XP_022034230.1		1.1765	2.3952	0.49119
XP_021981407.1	XP_021981626.1		1.0142	2.0642	0.49133
XP_021989736.1	XP_022004980.1		0.9908	2.0163	0.4914
XP_022006926.1	XP_022010101.1		0.3421	0.6961	0.49145
XP_021981395.1	XP_021984766.1		0.9881	2.0104	0.49149
XP_022010919.1	XP_035838717.1		0.9705	1.9744	0.49154
XP_022010292.1	XP_022029740.1		0.5998	1.2193	0.49192
XP_021993220.1	XP_022004455.1		1	2.031	0.49237
XP_022008275.1	XP_022014870.1		1.2931	2.6224	0.4931
XP_021973316.1	XP_021981395.1		1.0303	2.0888	0.49325
XP_022010919.1	XP_022013182.1		1.2882	2.6114	0.4933
XP_022002418.1	XP_022006668.1		0.9415	1.9082	0.4934
XP_021985239.1	XP_022000196.1		1.2862	2.6062	0.49352
XP_022013560.1	XP_035835830.1		0.6696	1.3564	0.49366
XP_021989734.1	XP_022034230.1		1.2297	2.4908	0.4937
XP_021989733.1	XP_022006327.1		1.322	2.6771	0.49382
XP_021988953.1	XP_022004455.1		1.0309	2.0837	0.49474
XP_021973011.1	XP_021993222.1		1.4926	3.0163	0.49484
XP_021978807.1	XP_021989736.1		1.1514	2.3251	0.4952
XP_021981388.1	XP_022039085.1		1.0929	2.2056	0.49551
XP_022039085.1	XP_035838717.1		1.2302	2.4826	0.49553
XP_021989736.1	XP_022013558.1		0.9614	1.9395	0.49569
XP_021992525.1	XP_022006327.1		1.1073	2.2331	0.49586
XP_021989737.1	XP_021993620.1		1.1224	2.2633	0.49591
XP_021993221.1	XP_022015084.1		1.3064	2.6311	0.49652
XP_021989733.1	XP_021993052.1		1.1198	2.2552	0.49654
XP_021978842.1	XP_022014870.1		1.101	2.2171	0.49659
XP_021992525.1	XP_022022487.2		1.319	2.6557	0.49667
XP_021989733.1	XP_022004455.1		0.9521	1.916	0.49692
XP_022000224.2	XP_022010295.2		0.8738	1.7584	0.49693
XP_021987457.1	XP_035838717.1		0.9635	1.9375	0.49729
XP_021969020.1	XP_022002417.1		0.971	1.9509	0.49772
XP_022010616.1	XP_022021912.1		1.1637	2.338	0.49773
XP_021987457.1	XP_022021913.1		1.2253	2.4596	0.49817
XP_022002420.1	XP_022006927.1		0.9942	1.9951	0.49832
XP_022008275.1	XP_022026964.1		0.7684	1.5417	0.49841
XP_021989734.1	XP_022013560.1		0.9664	1.9387	0.49848
XP_021981407.1	XP_021988229.1		1.2604	2.5284	0.4985
XP_022013182.1	XP_035835830.1		1.2442	2.4941	0.49886
XP_021993621.2	XP_022004455.1		0.9039	1.8117	0.49892
XP_022010296.1	XP_022039074.2		0.733	1.4672	0.49959
XP_021999258.1	XP_022010291.1		1.04	2.0815	0.49964
XP_021984981.2	XP_021993622.1		1.5881	3.1756	0.50009
XP_021981626.1	XP_022021911.1		1.4086	2.8165	0.50012
XP_021978807.1	XP_022010295.2		0.8675	1.7337	0.50037
XP_022006927.1	XP_022029738.1		0.9414	1.8812	0.50043
XP_021988229.1	XP_022002420.1		0.7525	1.503	0.50067
XP_022010295.2	XP_022034230.1		0.9055	1.8083	0.50075
XP_021989733.1	XP_022013560.1		0.9735	1.9432	0.50098
XP_021993621.2	XP_035833070.1		0.5565	1.1103	0.50122
XP_021978807.1	XP_022026964.1		1.3975	2.7852	0.50176
XP_021988953.1	XP_022015084.1		1.1895	2.37	0.5019
XP_021981388.1	XP_022004979.1		0.966	1.9237	0.50216
XP_021989734.1	XP_022004455.1		0.9974	1.9858	0.50227
XP_021989735.1	XP_022010296.1		1.0218	2.0342	0.50231
XP_022002421.1	XP_022006927.1		0.8445	1.6811	0.50235
XP_022006327.1	XP_022037775.1		1.18	2.3482	0.50251
XP_022010294.1	XP_022033162.1		0.8477	1.6849	0.50312
XP_021993621.2	XP_022022487.2		1.6889	3.3552	0.50337
XP_022009213.1	XP_022029739.1		1.0735	2.1324	0.50342
XP_022008275.1	XP_022021912.1		1.0519	2.0871	0.504
XP_022029740.1	XP_035835830.1		0.5068	1.0054	0.50408
XP_022029738.1	XP_035838717.1		1.1309	2.2434	0.5041
XP_022002418.1	XP_022009213.1		1.0124	2.0076	0.50428
XP_021993620.1	XP_022013182.1		1.0693	2.1203	0.50432
XP_022002420.1	XP_022021912.1		1.1244	2.2285	0.50455
XP_021984981.2	XP_022010295.2		1.1707	2.3189	0.50485
XP_021981388.1	XP_022005608.1		1.045	2.0692	0.50503
XP_022002415.1	XP_022009213.1		0.9888	1.9574	0.50516
XP_021976022.1	XP_022010101.1		1.3025	2.5755	0.50573
XP_021989734.1	XP_021993052.1		1.159	2.2905	0.506
XP_021981626.1	XP_022021914.1		1.4108	2.7881	0.50601
XP_022029738.1	XP_022039074.2		0.6698	1.3234	0.50612
XP_021969020.1	XP_022004455.1		1.0383	2.0508	0.50629
XP_021988229.1	XP_022005608.1		1.0248	2.0233	0.5065
XP_021992525.1	XP_022010292.1		1.2779	2.5215	0.5068
XP_022002417.1	XP_022010294.1		0.5481	1.08	0.5075
XP_021972810.1	XP_022006926.1		1.0246	2.0189	0.5075
XP_021995116.1	XP_022013560.1		1.2234	2.4101	0.50761
XP_021988229.1	XP_022013558.1		0.8465	1.6667	0.50789
XP_021985239.1	XP_022010101.1		1.1509	2.2648	0.50817

XP_021978807.1	XP_035835830.1	0.8655	1.7025	0.50837
XP_021989736.1	XP_022010292.1	1.0343	2.0342	0.50846
XP_022009213.1	XP_022039085.1	1.2236	2.4041	0.50896
XP_021988229.1	XP_022009213.1	1.255	2.4648	0.50917
XP_022010292.1	XP_022039074.2	0.6989	1.3721	0.50937
XP_021968895.1	XP_022010296.1	0.816	1.6016	0.50949
XP_021987457.1	XP_022039074.2	1.1874	2.3302	0.50957
XP_021995117.1	XP_022000224.2	1.1688	2.2927	0.50979
XP_022013183.1	XP_022014870.1	1.0685	2.0957	0.50985
XP_021981388.1	XP_022029739.1	1.093	2.1424	0.51018
XP_021993221.1	XP_022004455.1	1.0229	2.0045	0.5103
XP_022002417.1	XP_022022487.2	0.9062	1.7757	0.51033
XP_022004455.1	XP_022008276.2	1.0502	2.0572	0.5105
XP_022034230.1	XP_022037775.1	1.2574	2.4612	0.51089
XP_022005608.1	XP_022029738.1	0.9174	1.7947	0.51117
XP_021981407.1	XP_022010292.1	1.019	1.9929	0.51132
XP_022006927.1	XP_022010295.2	0.9167	1.79	0.51212
XP_021968895.1	XP_021989734.1	1.2226	2.3868	0.51223
XP_022002417.1	XP_022010292.1	0.5092	0.9937	0.51243
XP_021987458.1	XP_022026964.1	1.2143	2.3694	0.51249
XP_022008276.2	XP_022021914.1	1.306	2.5465	0.51286
XP_022022487.2	XP_022035552.1	1.2703	2.475	0.51325
XP_021981626.1	XP_021988229.1	1.5229	2.9663	0.5134
XP_021981395.1	XP_022021912.1	1.1753	2.2892	0.51341
XP_021984981.2	XP_021995117.1	1.1492	2.2377	0.51356
XP_021989733.1	XP_035833070.1	1.2476	2.429	0.51363
XP_021989734.1	XP_022004980.1	0.9379	1.824	0.5142
XP_022010101.1	XP_022010292.1	0.881	1.7132	0.51424
XP_022010101.1	XP_022013183.1	1.3728	2.669	0.51435
XP_022002420.1	XP_022006926.1	0.8915	1.7332	0.51437
XP_021993620.1	XP_021993622.1	1.2625	2.4531	0.51465
XP_021973011.1	XP_022022487.2	1.4286	2.775	0.51481
XP_021978807.1	XP_021981388.1	1.0589	2.0561	0.515
XP_021978842.1	XP_021989734.1	0.8833	1.7146	0.51516
XP_021993221.1	XP_022002416.1	0.9415	1.8275	0.51518
XP_022002415.1	XP_022035551.1	0.9697	1.8822	0.51519
XP_022002415.1	XP_022035552.1	0.9697	1.8822	0.51519
XP_022008276.2	XP_022021911.1	1.3312	2.5792	0.51613
XP_021981407.1	XP_022013239.1	1.3724	2.6585	0.51623
XP_021993222.1	XP_022006327.1	1.5406	2.9812	0.51677
XP_021993222.1	XP_022002420.1	1.0544	2.0403	0.51679
XP_021987458.1	XP_022009213.1	1.0051	1.9448	0.51681
XP_021989736.1	XP_022013560.1	0.8777	1.6981	0.51687
XP_022002419.1	XP_022006926.1	0.8521	1.6479	0.51708
XP_022013560.1	XP_022029739.1	0.6647	1.2849	0.51732
XP_021989737.1	XP_022002417.1	0.9765	1.8876	0.51732
XP_021969020.1	XP_021985239.1	1.5735	3.0394	0.5177
XP_021973011.1	XP_021995117.1	1.1824	2.2834	0.51782
XP_021985239.1	XP_021989735.1	1.1406	2.2021	0.51796
XP_022010616.1	XP_022022487.2	1.0795	2.0817	0.51857
XP_021973854.1	XP_021995117.1	1.4101	2.7191	0.51859
XP_022026963.1	XP_035835830.1	1.2524	2.4142	0.51876
XP_022000196.1	XP_022001254.1	1.2725	2.4526	0.51884
XP_021984981.2	XP_022026963.1	1.254	2.4167	0.51889
XP_021984981.2	XP_022022487.2	1.3006	2.5045	0.51931
XP_021973854.1	XP_022009213.1	1.5381	2.9614	0.51938
XP_022000224.2	XP_022010296.1	0.8297	1.5972	0.51947
XP_021993621.2	XP_022035551.1	1.2868	2.4764	0.51963
XP_021993621.2	XP_022035552.1	1.2868	2.4764	0.51963
XP_022004979.1	XP_022037775.1	1.3269	2.5509	0.52017
XP_022029739.1	XP_022033162.1	0.8126	1.5613	0.52046
XP_021993551.1	XP_022006327.1	1.6479	3.1636	0.52089
XP_022004980.1	XP_022029738.1	1.2224	2.3454	0.52119
XP_021985239.1	XP_022022835.1	1.1321	2.1717	0.5213
XP_021992525.1	XP_021995117.1	1.1064	2.1204	0.52179
XP_021978842.1	XP_021981626.1	1.3361	2.5604	0.52183
XP_021969020.1	XP_021978842.1	1.6754	3.2096	0.522
XP_022010292.1	XP_022039085.1	0.6722	1.2874	0.52214
XP_022002416.1	XP_022006927.1	0.9266	1.7737	0.52241
XP_022014870.1	XP_022015084.1	1.1157	2.1352	0.52253
XP_021993220.1	XP_022002421.1	0.9911	1.8967	0.52254
XP_021988953.1	XP_035835830.1	1.2375	2.3678	0.52264
XP_021989735.1	XP_021993551.1	1.3175	2.5191	0.523
XP_021988953.1	XP_021989733.1	0.9921	1.8963	0.52318
XP_021989735.1	XP_022010294.1	0.9536	1.8227	0.52318
XP_021993221.1	XP_022035552.1	1.4253	2.7235	0.52333
XP_021986500.1	XP_022006927.1	1.003	1.9162	0.52343
XP_022000224.2	XP_022002421.1	0.8826	1.6861	0.52346
XP_021989736.1	XP_035833070.1	1.3375	2.555	0.52348
XP_022000196.1	XP_022010919.1	1.3634	2.6044	0.5235
XP_021972810.1	XP_022033162.1	0.7017	1.3403	0.52354
XP_022029622.1	XP_022039074.2	1.2813	2.4458	0.52388
XP_022008276.2	XP_022010296.1	1.0518	2.0061	0.5243
XP_022010294.1	XP_022021911.1	1.1553	2.2029	0.52445
XP_022010294.1	XP_022021913.1	1.1553	2.2029	0.52445
XP_022010294.1	XP_022021914.1	1.1553	2.2029	0.52445
XP_021989737.1	XP_022035552.1	0.8223	1.5668	0.52483
XP_035835830.1	XP_035838717.1	0.8931	1.7004	0.52523

XP_021993221.1	XP_022002415.1	0.936	1.782	0.52525
XP_022013182.1	XP_022014870.1	1.053	2.0042	0.5254
XP_021981407.1	XP_022029740.1	1.1011	2.0955	0.52546
XP_021995116.1	XP_022010295.2	1.0422	1.9834	0.52546
XP_022010294.1	XP_022029739.1	0.6885	1.31	0.52557
XP_022001254.1	XP_022002420.1	0.9291	1.7664	0.52599
XP_022004979.1	XP_022039085.1	1.0589	2.0126	0.52614
XP_021973855.1	XP_022022487.2	1.6152	3.069	0.5263
XP_022002419.1	XP_022002420.1	0.1377	0.2616	0.52638
XP_021978842.1	XP_022010296.1	0.983	1.8672	0.52646
XP_022006926.1	XP_035842399.1	1.3703	2.6011	0.52682
XP_022006668.1	XP_022013560.1	0.9319	1.7685	0.52694
XP_021981388.1	XP_021993620.1	1.0236	1.9425	0.52695
XP_022006926.1	XP_022008275.1	1.1181	2.1218	0.52696
XP_022010294.1	XP_022029740.1	0.6149	1.166	0.52736
XP_021993222.1	XP_022002415.1	1.0041	1.9028	0.5277
XP_021993620.1	XP_022004455.1	0.9469	1.794	0.52781
XP_021973854.1	XP_021988953.1	1.7248	3.2665	0.52803
XP_022002421.1	XP_022022835.1	1.2712	2.4057	0.52841
XP_022004980.1	XP_022034230.1	1.1451	2.1663	0.5286
XP_021978842.1	XP_021995117.1	1.1046	2.0888	0.52882
XP_022002416.1	XP_022010292.1	0.5067	0.9579	0.52897
XP_021972810.1	XP_022013558.1	0.6349	1.2002	0.529
XP_022004455.1	XP_022021911.1	0.9388	1.7745	0.52905
XP_022004455.1	XP_022021913.1	0.9388	1.7745	0.52905
XP_022004455.1	XP_022021914.1	0.9388	1.7745	0.52905
XP_022022835.1	XP_022034230.1	1.2031	2.2734	0.52921
XP_021969020.1	XP_022001254.1	1.2681	2.395	0.52948
XP_022010101.1	XP_022013558.1	0.8806	1.6626	0.52965
XP_021968895.1	XP_022029740.1	0.7809	1.4743	0.52968
XP_022002419.1	XP_022010294.1	0.6243	1.1782	0.52988
XP_021969020.1	XP_022002418.1	0.961	1.8131	0.53003
XP_021987457.1	XP_022029740.1	1.14	2.1505	0.53011
XP_022035384.1	XP_035835830.1	1.5986	3.0155	0.53013
XP_022002420.1	XP_022018622.1	0.9323	1.758	0.53032
XP_021993621.2	XP_035842399.1	1.4208	2.6777	0.5306
XP_022008275.1	XP_022022835.1	1.1283	2.1249	0.53099
XP_022034230.1	XP_022039085.1	1.154	2.1732	0.53101
XP_021993622.1	XP_022039074.2	1.37	2.5792	0.53117
XP_021993622.1	XP_022013183.1	1.4626	2.7533	0.53122
XP_022001254.1	XP_022039085.1	1.1499	2.1639	0.5314
XP_021973855.1	XP_021981407.1	1.645	3.0949	0.53152
XP_021993622.1	XP_022010101.1	1.8554	3.4905	0.53156
XP_021987457.1	XP_022002421.1	1.1763	2.2102	0.53221
XP_021986500.1	XP_022002419.1	0.8839	1.66	0.53247
XP_021973854.1	XP_021976022.1	1.5389	2.8901	0.53247
XP_021984766.1	XP_022010616.1	1.3683	2.5694	0.53254
XP_021987458.1	XP_022029740.1	1.156	2.1707	0.53255
XP_021995117.1	XP_022029740.1	0.9891	1.8567	0.53272
XP_022006327.1	XP_022008276.2	1.3298	2.496	0.53277
XP_022002419.1	XP_022021911.1	1.0314	1.9355	0.53289
XP_022002419.1	XP_022021913.1	1.0314	1.9355	0.53289
XP_022002419.1	XP_022021914.1	1.0314	1.9355	0.53289
XP_021999258.1	XP_022013239.1	1.8351	3.4422	0.53312
XP_022010919.1	XP_022026964.1	1.2805	2.4004	0.53345
XP_022026963.1	XP_022029739.1	1.2036	2.2557	0.53358
XP_022002420.1	XP_022006327.1	1.1747	2.2006	0.53381
XP_021984766.1	XP_021993620.1	1.0409	1.9499	0.53382
XP_022004455.1	XP_035835830.1	0.5148	0.9638	0.53414
XP_021978842.1	XP_021988229.1	1.0923	2.0449	0.53416
XP_021987458.1	XP_022002421.1	1.1922	2.2306	0.53448
XP_021989733.1	XP_022002416.1	0.9608	1.7974	0.53455
XP_021988229.1	XP_022013182.1	1.2989	2.4284	0.53488
XP_022006926.1	XP_022029739.1	0.981	1.8338	0.53495
XP_021973854.1	XP_022010292.1	1.07	1.9998	0.53505
XP_021989735.1	XP_035835830.1	1.0004	1.8682	0.53549
XP_021968895.1	XP_022029738.1	0.8463	1.5785	0.53614
XP_022002418.1	XP_022034230.1	0.989	1.8432	0.53657
XP_021989736.1	XP_022002420.1	0.8592	1.601	0.53666
XP_021976022.1	XP_022002420.1	1.0041	1.871	0.53666
XP_021981388.1	XP_021993621.2	0.9808	1.8271	0.53681
XP_021989736.1	XP_022010296.1	1.0185	1.8972	0.53684
XP_021987458.1	XP_022010919.1	1.4386	2.6794	0.53691
XP_021973855.1	XP_021993220.1	2.0705	3.8562	0.53693
XP_022035551.1	XP_022039074.2	1.294	2.4087	0.53722
XP_022035552.1	XP_022039074.2	1.294	2.4087	0.53722
XP_021993621.2	XP_022002419.1	0.9223	1.7161	0.53744
XP_021973854.1	XP_022029622.1	1.7074	3.1756	0.53766
XP_022004979.1	XP_022021912.1	1.2665	2.3541	0.538
XP_021973011.1	XP_022008276.2	1.2735	2.3668	0.53807
XP_022021911.1	XP_035835830.1	1.1504	2.137	0.53832
XP_022021913.1	XP_035835830.1	1.1504	2.137	0.53832
XP_022021914.1	XP_035835830.1	1.1504	2.137	0.53832
XP_021972810.1	XP_022004455.1	0.5559	1.0314	0.53898
XP_021984766.1	XP_022018622.1	1.4017	2.5983	0.53947
XP_022008276.2	XP_022026963.1	1.4825	2.7477	0.53954
XP_021989733.1	XP_022029738.1	1.1583	2.1463	0.53967
XP_022013239.1	XP_022037775.1	1.677	3.1074	0.53968

XP_021989737.1	XP_022035551.1	0.8196	1.5183	0.53981
XP_021973854.1	XP_022034230.1	1.4577	2.7	0.53989
XP_021989733.1	XP_022026963.1	1.3213	2.4447	0.54048
XP_021988229.1	XP_021993222.1	1.466	2.7115	0.54066
XP_021988229.1	XP_022013183.1	1.3156	2.4331	0.54071
XP_022006926.1	XP_022033162.1	1.1468	2.1203	0.54087
XP_021972810.1	XP_022000224.2	0.9069	1.6752	0.54137
XP_021989737.1	XP_022034230.1	1.1837	2.1862	0.54144
XP_021986500.1	XP_022002417.1	0.7796	1.4397	0.5415
XP_022006926.1	XP_022010296.1	0.8857	1.6352	0.54165
XP_021989735.1	XP_022006926.1	1.293	2.3866	0.54177
XP_021987458.1	XP_035838717.1	0.9683	1.7867	0.54195
XP_022010292.1	XP_022014870.1	0.8088	1.4917	0.5422
XP_021981395.1	XP_022026963.1	1.5519	2.8619	0.54226
XP_022000196.1	XP_022026963.1	1.2482	2.3003	0.54262
XP_021972810.1	XP_021993221.1	1.3114	2.4161	0.54278
XP_021987457.1	XP_022021914.1	1.2004	2.2111	0.5429
XP_021985239.1	XP_021987458.1	0.9717	1.7898	0.54291
XP_021989737.1	XP_022013558.1	0.9337	1.7191	0.54313
XP_021978807.1	XP_021987457.1	1.0131	1.865	0.54322
XP_021978807.1	XP_021987458.1	1.0131	1.865	0.54322
XP_021978842.1	XP_022010292.1	0.9551	1.7581	0.54326
XP_022010291.1	XP_022035551.1	1.2121	2.2299	0.54357
XP_022010291.1	XP_022035552.1	1.2121	2.2299	0.54357
XP_022010295.2	XP_022022835.1	1.0695	1.9673	0.54364
XP_021981395.1	XP_022039074.2	1.4333	2.6353	0.54388
XP_022010291.1	XP_022037775.1	1.0376	1.9074	0.54399
XP_021978807.1	XP_021981407.1	1.0877	1.9989	0.54415
XP_021995117.1	XP_022010291.1	1.035	1.9016	0.54428
XP_021969020.1	XP_021992525.1	1.2929	2.3746	0.54447
XP_022002415.1	XP_022006668.1	1.0183	1.8697	0.54463
XP_021988229.1	XP_035838717.1	1.26	2.3134	0.54465
XP_021988953.1	XP_022026963.1	1.2537	2.2997	0.54516
XP_021989734.1	XP_035835830.1	1.0591	1.9427	0.54517
XP_021985239.1	XP_022002421.1	0.8112	1.4877	0.54527
XP_021993621.2	XP_035835830.1	1.1804	2.1646	0.54532
XP_021973316.1	XP_021989733.1	1.3202	2.4209	0.54533
XP_021988953.1	XP_022029622.1	1.2822	2.3509	0.54541
XP_021993283.1	XP_035833070.1	1.6914	3.1002	0.54558
XP_021973011.1	XP_021987458.1	1.2856	2.3541	0.54611
XP_021984981.2	XP_022004455.1	0.9801	1.794	0.54632
XP_021988229.1	XP_035835830.1	0.6686	1.2236	0.54642
XP_022002416.1	XP_022010296.1	0.5164	0.9434	0.54738
XP_021993620.1	XP_022029738.1	1.1236	2.0525	0.54743
XP_022010295.2	XP_035835830.1	0.7302	1.3328	0.54787
XP_021989735.1	XP_022035552.1	0.9475	1.7293	0.54791
XP_021993621.2	XP_022002421.1	0.8914	1.6268	0.54795
XP_022000224.2	XP_022006668.1	1.033	1.8849	0.54804
XP_021968895.1	XP_022002417.1	0.7902	1.4414	0.54822
XP_022029738.1	XP_022035384.1	1.3203	2.4071	0.5485
XP_021985239.1	XP_021987457.1	0.9699	1.7676	0.54871
XP_021973011.1	XP_021989734.1	1.1056	2.0147	0.54877
XP_021981626.1	XP_022014870.1	1.0136	1.847	0.54878
XP_021981407.1	XP_022010101.1	1.2892	2.3488	0.54888
XP_021985239.1	XP_022013239.1	1.2429	2.2627	0.5493
XP_021986500.1	XP_022010919.1	1.183	2.1531	0.54944
XP_022010101.1	XP_022029739.1	1.018	1.8526	0.5495
XP_022034230.1	XP_035838717.1	1.1218	2.0413	0.54955
XP_021989734.1	XP_022014870.1	0.9584	1.7439	0.54957
XP_021972810.1	XP_021981407.1	1.0417	1.8953	0.54962
XP_022002420.1	XP_022021911.1	1.1279	2.052	0.54966
XP_022002420.1	XP_022021913.1	1.1279	2.052	0.54966
XP_022002420.1	XP_022021914.1	1.1279	2.052	0.54966
XP_021986500.1	XP_022002418.1	0.7794	1.4176	0.5498
XP_022008275.1	XP_022010101.1	1.2915	2.349	0.54981
XP_021968895.1	XP_021989736.1	1.1583	2.1048	0.55031
XP_021968895.1	XP_022002421.1	0.7363	1.3376	0.55046
XP_021993222.1	XP_022002417.1	1.0596	1.9237	0.55081
XP_021993621.2	XP_022013183.1	1.239	2.249	0.55091
XP_021981626.1	XP_022004455.1	0.9363	1.6992	0.55102
XP_021984981.2	XP_022010919.1	1.0075	1.8279	0.55118
XP_022006926.1	XP_022026964.1	1.3391	2.429	0.5513
XP_022026963.1	XP_022029738.1	1.0498	1.903	0.55166
XP_022002420.1	XP_022013182.1	1.1458	2.077	0.55166
XP_021981395.1	XP_022006927.1	1.4122	2.5598	0.55168
XP_022000224.2	XP_022022487.2	1.0403	1.8854	0.55177
XP_021985239.1	XP_022029738.1	0.8776	1.5902	0.55188
XP_021978842.1	XP_022000224.2	1.2989	2.3532	0.55197
XP_021984766.1	XP_022006668.1	1.436	2.5999	0.55233
XP_021973854.1	XP_021988229.1	1.5573	2.8194	0.55235
XP_022002416.1	XP_022005608.1	0.8524	1.5432	0.55236
XP_021989734.1	XP_022026963.1	1.3481	2.4391	0.5527
XP_022013183.1	XP_035835830.1	1.2574	2.2748	0.55275
XP_022010291.1	XP_022029622.1	1.0508	1.9003	0.55297
XP_022004455.1	XP_022034230.1	0.9791	1.7702	0.5531
XP_021969020.1	XP_022002419.1	1.0734	1.94	0.5533
XP_022002417.1	XP_022006926.1	0.8842	1.5976	0.55346
XP_021993220.1	XP_022006926.1	1.1718	2.1141	0.55428

XP_021986500.1	XP_022002415.1	0.7983	1.4397	0.55449
XP_021989735.1	XP_022010101.1	1.1023	1.9861	0.55501
XP_021985239.1	XP_022006327.1	1.4861	2.6771	0.55512
XP_022005608.1	XP_022021911.1	1.2549	2.2605	0.55514
XP_022005608.1	XP_022021913.1	1.2549	2.2605	0.55514
XP_022005608.1	XP_022021914.1	1.2549	2.2605	0.55514
XP_021987457.1	XP_022021911.1	1.1651	2.0982	0.55529
XP_022002418.1	XP_022006927.1	0.9152	1.6479	0.55537
XP_022010294.1	XP_022029738.1	0.6874	1.2376	0.55543
XP_022029740.1	XP_022034230.1	0.9879	1.777	0.55594
XP_022002418.1	XP_022004980.1	1.2359	2.2225	0.55609
XP_021981407.1	XP_022010296.1	1.1382	2.0467	0.55611
XP_021973011.1	XP_021985239.1	1.1159	2.0066	0.55611
XP_021987457.1	XP_035835830.1	0.973	1.7496	0.55613
XP_021987458.1	XP_035835830.1	0.973	1.7496	0.55613
XP_021989736.1	XP_022010294.1	0.9132	1.6418	0.55622
XP_021993221.1	XP_022002420.1	0.9916	1.7827	0.55623
XP_022010296.1	XP_022035384.1	0.9609	1.7275	0.55624
XP_022033162.1	XP_035833070.1	1.2353	2.2206	0.55629
XP_022002421.1	XP_022010294.1	0.6313	1.1348	0.55631
XP_021973316.1	XP_035838717.1	1.2511	2.2485	0.55642
XP_021993220.1	XP_022013558.1	0.9475	1.7028	0.55644
XP_021993052.1	XP_022021912.1	1.0921	1.962	0.55663
XP_021981626.1	XP_022010919.1	1.4635	2.6278	0.55693
XP_021981407.1	XP_021984766.1	0.8871	1.5928	0.55694
XP_021992525.1	XP_022010919.1	1.0782	1.9352	0.55715
XP_022010295.2	XP_035842399.1	1.0054	1.8039	0.55735
XP_022009213.1	XP_022034230.1	1.1615	2.0839	0.55737
XP_021973011.1	XP_022004455.1	1.0011	1.7959	0.55744
XP_021995117.1	XP_022013182.1	1.1707	2.0993	0.55766
XP_021989736.1	XP_022014870.1	0.9021	1.617	0.55788
XP_021973316.1	XP_021993621.2	1.5446	2.7684	0.55794
XP_021985239.1	XP_022002415.1	0.7878	1.4112	0.55825
XP_021999258.1	XP_022002420.1	1.0145	1.8165	0.55849
XP_021973316.1	XP_021993620.1	1.2064	2.1599	0.55854
XP_021999258.1	XP_022002416.1	1.0995	1.9685	0.55855
XP_021987457.1	XP_022033162.1	1.1589	2.0746	0.55861
XP_022002417.1	XP_022005608.1	0.8492	1.5199	0.55872
XP_021981626.1	XP_021989734.1	1.1585	2.0715	0.55926
XP_021993621.2	XP_022039085.1	1.1254	2.0108	0.55968
XP_022008276.2	XP_022029740.1	1.1005	1.9662	0.55971
XP_021993221.1	XP_022035551.1	1.4226	2.5406	0.55995
XP_022000196.1	XP_022034230.1	1.4182	2.5326	0.55998
XP_021989737.1	XP_022002416.1	0.913	1.6302	0.56005
XP_021993221.1	XP_022002418.1	0.9393	1.6768	0.56017
XP_022002420.1	XP_022022835.1	0.9842	1.7569	0.56019
XP_022006668.1	XP_035842399.1	1.2859	2.2954	0.56021
XP_021969020.1	XP_022008276.2	1.6198	2.8911	0.56027
XP_022021911.1	XP_022026964.1	1.4328	2.5561	0.56054
XP_022021913.1	XP_022026964.1	1.4328	2.5561	0.56054
XP_022021914.1	XP_022026964.1	1.4328	2.5561	0.56054
XP_021973011.1	XP_021981395.1	0.9281	1.6556	0.56058
XP_022008275.1	XP_022022487.2	1.5125	2.698	0.5606
XP_022002419.1	XP_035833070.1	1.1154	1.9888	0.56084
XP_022000224.2	XP_022022835.1	1.1176	1.992	0.56104
XP_022002421.1	XP_022010292.1	0.5767	1.0272	0.56143
XP_022014870.1	XP_022035384.1	1.0582	1.8845	0.56153
XP_021973011.1	XP_021993622.1	1.252	2.2287	0.56176
XP_021973011.1	XP_022039085.1	1.1728	2.0873	0.56187
XP_022002418.1	XP_022010294.1	0.5844	1.0397	0.56209
XP_021981395.1	XP_022034230.1	1.244	2.2115	0.56251
XP_022008276.2	XP_022021913.1	1.3477	2.3956	0.56257
XP_022004980.1	XP_022008275.1	0.8751	1.5551	0.56273
XP_022010919.1	XP_022022835.1	1.1647	2.0696	0.56277
XP_022002417.1	XP_022013558.1	0.4812	0.8549	0.56287
XP_021973316.1	XP_022018622.1	1.3966	2.4809	0.56294
XP_022002421.1	XP_022033162.1	0.7124	1.2654	0.56298
XP_021989735.1	XP_022035551.1	0.9487	1.685	0.56303
XP_021969020.1	XP_022010292.1	1.2763	2.2667	0.56307
XP_022002415.1	XP_022010294.1	0.5627	0.9992	0.56315
XP_022013183.1	XP_022033162.1	1.2621	2.2411	0.56316
XP_022006668.1	XP_035833070.1	1.4029	2.491	0.56319
XP_022022487.2	XP_022033162.1	1.1618	2.0628	0.56322
XP_022001254.1	XP_022010296.1	1.1322	2.01	0.56328
XP_021972810.1	XP_022010295.2	0.7199	1.278	0.5633
XP_021989733.1	XP_022002420.1	0.9396	1.6669	0.56368
XP_021968895.1	XP_022010295.2	0.8247	1.4627	0.56382
XP_021989734.1	XP_022005608.1	0.9246	1.6397	0.56388
XP_022002421.1	XP_022037775.1	1.094	1.9395	0.56406
XP_022002421.1	XP_022009213.1	1.0506	1.8623	0.56414
XP_021985239.1	XP_022002418.1	0.7876	1.3956	0.56435
XP_021993052.1	XP_021993221.1	1.1444	2.0278	0.56436
XP_021968895.1	XP_021993220.1	1.3733	2.4333	0.56438
XP_021973011.1	XP_022002417.1	0.9579	1.697	0.56447
XP_021995116.1	XP_022002419.1	1.1424	2.0236	0.56454
XP_021978842.1	XP_022010295.2	0.9507	1.6836	0.56468
XP_021995117.1	XP_022039085.1	1.0544	1.867	0.56476
XP_021993620.1	XP_022006668.1	1.4201	2.5136	0.56497

XP_021969020.1	XP_021988953.1	1.332	2.3575	0.56501
XP_021988229.1	XP_021989737.1	1.1391	2.0153	0.56523
XP_022004979.1	XP_022022835.1	1.2351	2.1849	0.56529
XP_021984766.1	XP_021989736.1	0.9496	1.679	0.56557
XP_022004979.1	XP_022015084.1	1.1559	2.0434	0.56567
XP_021989737.1	XP_022008276.2	1.4231	2.5152	0.5658
XP_021985239.1	XP_022035551.1	1.0482	1.8513	0.5662
XP_021985239.1	XP_022035552.1	1.0482	1.8513	0.5662
XP_021993620.1	XP_022015084.1	1.1314	1.9982	0.56621
XP_021981407.1	XP_022000196.1	1.141	2.0143	0.56645
XP_021968895.1	XP_022002416.1	0.7791	1.3751	0.56658
XP_021989735.1	XP_022034230.1	1.1465	2.0232	0.56668
XP_022015084.1	XP_022026963.1	1.1291	1.9918	0.56687
XP_022002417.1	XP_022010296.1	0.538	0.9487	0.56709
XP_022001254.1	XP_022029738.1	1.1396	2.0093	0.56716
XP_021993052.1	XP_022010101.1	0.8943	1.5761	0.56741
XP_022004979.1	XP_022033162.1	1.2906	2.2744	0.56745
XP_022013239.1	XP_022029739.1	1.322	2.3278	0.56792
XP_021972810.1	XP_021988953.1	1.2446	2.1913	0.56797
XP_021989735.1	XP_021993052.1	1.0854	1.91	0.56827
XP_022010616.1	XP_022014870.1	0.8843	1.5559	0.56835
XP_021978807.1	XP_021985239.1	0.8221	1.4463	0.56842
XP_022002416.1	XP_022004980.1	1.191	2.0945	0.56863
XP_022002417.1	XP_022021913.1	1.1176	1.9651	0.56872
XP_022002417.1	XP_022021914.1	1.1176	1.9651	0.56872
XP_021993622.1	XP_022029738.1	1.3804	2.4271	0.56874
XP_021973011.1	XP_022002415.1	0.9261	1.6274	0.56907
XP_022000224.2	XP_022010919.1	0.9725	1.708	0.56938
XP_021993283.1	XP_022022835.1	1.5482	2.7191	0.56938
XP_021987458.1	XP_022001254.1	1.2349	2.1667	0.56995
XP_021992525.1	XP_021993620.1	1.1386	1.9977	0.56996
XP_021972810.1	XP_022010292.1	0.6538	1.1468	0.57011
XP_021978842.1	XP_022037775.1	1.2197	2.1389	0.57025
XP_021993221.1	XP_021993622.1	1.369	2.3999	0.57044
XP_022013558.1	XP_022029740.1	0.611	1.0711	0.57044
XP_021972810.1	XP_021987457.1	1.1866	2.08	0.57048
XP_021989733.1	XP_022002418.1	1.0048	1.7605	0.57075
XP_022006927.1	XP_022010296.1	1.0404	1.8218	0.57108
XP_022013183.1	XP_022034230.1	1.1185	1.9584	0.57113
XP_021987457.1	XP_022010919.1	1.4279	2.5001	0.57114
XP_021978842.1	XP_022013239.1	1.4252	2.4953	0.57115
XP_022021912.1	XP_035835830.1	1.1224	1.9651	0.57117
XP_021993620.1	XP_022029740.1	0.8828	1.5446	0.57154
XP_021993222.1	XP_022002416.1	1.0233	1.7902	0.57161
XP_021993620.1	XP_022010291.1	1.1385	1.9912	0.57177
XP_021981626.1	XP_022010294.1	1.1087	1.9379	0.57211
XP_021973854.1	XP_022010295.2	1.1902	2.08	0.57221
XP_022002415.1	XP_022035384.1	1.2751	2.2278	0.57236
XP_022004455.1	XP_022010291.1	0.5426	0.9474	0.57273
XP_021995116.1	XP_022010294.1	1.2123	2.1167	0.57273
XP_021984766.1	XP_022000224.2	1.1793	2.0578	0.57309
XP_022002420.1	XP_022035384.1	1.3184	2.3003	0.57314
XP_021973011.1	XP_022006668.1	1.2485	2.1773	0.57342
XP_021993221.1	XP_022029740.1	1.1389	1.9861	0.57344
XP_021978842.1	XP_022039085.1	1.3065	2.2776	0.57363
XP_021988229.1	XP_021993620.1	1.0606	1.8486	0.57373
XP_022009213.1	XP_022015084.1	1.1572	2.0166	0.57384
XP_021984981.2	XP_022010291.1	1.3145	2.29	0.57402
XP_021989733.1	XP_022026964.1	1.1481	1.9996	0.57416
XP_022002419.1	XP_035835830.1	0.4153	0.7231	0.57433
XP_022006668.1	XP_022029739.1	1.051	1.8298	0.57438
XP_021988229.1	XP_022022487.2	1.0552	1.8364	0.5746
XP_021984766.1	XP_022002421.1	1.1375	1.9788	0.57484
XP_022013239.1	XP_035835830.1	1.1141	1.9377	0.57496
XP_021984766.1	XP_035835830.1	1.1622	2.0213	0.57498
XP_022004455.1	XP_022035551.1	0.9562	1.6626	0.57512
XP_022004455.1	XP_022035552.1	0.9562	1.6626	0.57512
XP_021988229.1	XP_021992525.1	1.2421	2.1597	0.57513
XP_021973854.1	XP_022018622.1	1.3203	2.2954	0.57519
XP_022002420.1	XP_022039085.1	0.6115	1.063	0.57526
XP_021972810.1	XP_022013560.1	0.7977	1.3864	0.57538
XP_022006926.1	XP_022026963.1	1.1362	1.9736	0.5757
XP_021999258.1	XP_022015084.1	1.4174	2.462	0.57571
XP_021984766.1	XP_021993220.1	1.4247	2.4743	0.5758
XP_021988953.1	XP_022002420.1	0.9905	1.7195	0.57604
XP_022010295.2	XP_022039085.1	0.7575	1.3142	0.5764
XP_021993620.1	XP_022022835.1	1.1923	2.068	0.57655
XP_021989737.1	XP_022002415.1	0.9155	1.5878	0.57658
XP_022002421.1	XP_022006668.1	1.1371	1.9714	0.5768
XP_022004455.1	XP_022026964.1	0.9455	1.6391	0.57684
XP_022010296.1	XP_022029740.1	0.7011	1.2153	0.57689
XP_021981626.1	XP_022010292.1	0.9983	1.7284	0.57759
XP_022013560.1	XP_022014870.1	1.0638	1.8417	0.57762
XP_022013239.1	XP_022013560.1	1.0024	1.7353	0.57765
XP_021972810.1	XP_021973316.1	1.4664	2.5383	0.57771
XP_022000224.2	XP_022004455.1	0.8745	1.5136	0.57776
XP_021993621.2	XP_035838717.1	1.0962	1.8972	0.5778
XP_021993220.1	XP_022010292.1	0.9661	1.6712	0.57809

XP_021987457.1	XP_022026964.1	1.2277	2.1222	0.5785
XP_021988953.1	XP_022006668.1	1.3106	2.2651	0.57861
XP_022001254.1	XP_022010291.1	1.1676	2.0179	0.57862
XP_021968895.1	XP_021988953.1	1.2049	2.0819	0.57875
XP_022013560.1	XP_022022487.2	0.9845	1.7007	0.57888
XP_022014870.1	XP_022021913.1	1.1943	2.0613	0.57939
XP_022014870.1	XP_022021914.1	1.1943	2.0613	0.57939
XP_022002418.1	XP_022035384.1	1.3686	2.3601	0.57989
XP_022004455.1	XP_035838717.1	0.9262	1.5966	0.58011
XP_022013239.1	XP_022029740.1	1.005	1.7312	0.58052
XP_021978842.1	XP_022013558.1	0.9483	1.6328	0.58078
XP_021993620.1	XP_022010295.2	0.9838	1.6937	0.58086
XP_021993052.1	XP_022008276.2	1.1399	1.962	0.58099
XP_022029738.1	XP_022039085.1	0.7497	1.2903	0.58103
XP_021973011.1	XP_022013182.1	1.1605	1.9969	0.58115
XP_021973011.1	XP_022013183.1	1.1605	1.9969	0.58115
XP_021987458.1	XP_022004980.1	1.3612	2.3422	0.58116
XP_021969020.1	XP_021976022.1	1.4652	2.5203	0.58136
XP_022013558.1	XP_022026964.1	0.9289	1.5977	0.5814
XP_021978807.1	XP_022015084.1	1.1412	1.9624	0.58153
XP_022018622.1	XP_022029622.1	1.2831	2.2062	0.58159
XP_022021912.1	XP_022026964.1	1.3524	2.3251	0.58165
XP_022037775.1	XP_035833070.1	1.8256	3.1384	0.5817
XP_021972810.1	XP_021986500.1	0.8712	1.4974	0.58181
XP_022008275.1	XP_022013183.1	1.0846	1.8637	0.58196
XP_022008276.2	XP_022010101.1	1.0603	1.8216	0.58207
XP_022006668.1	XP_022029622.1	1.4413	2.4757	0.58218
XP_022001254.1	XP_022022487.2	1.2189	2.0936	0.5822
XP_022006927.1	XP_022026963.1	1.1176	1.9191	0.58236
XP_021989733.1	XP_035835830.1	0.9819	1.6858	0.58245
XP_022002419.1	XP_022022487.2	0.9638	1.6547	0.58246
XP_021984766.1	XP_022002417.1	1.1944	2.0504	0.58252
XP_021976022.1	XP_022008276.2	1.3785	2.3663	0.58256
XP_022014870.1	XP_022021912.1	1.2425	2.1324	0.58268
XP_021988953.1	XP_022006927.1	1.2298	2.1101	0.58282
XP_021981388.1	XP_022029740.1	1.1542	1.9803	0.58284
XP_021978807.1	XP_022029740.1	0.9607	1.6479	0.58298
XP_021993220.1	XP_022039085.1	1.3476	2.3114	0.58302
XP_022002416.1	XP_022022487.2	0.904	1.5504	0.58308
XP_022002418.1	XP_022002419.1	0.1488	0.2551	0.5833
XP_021969020.1	XP_022006668.1	1.6368	2.8058	0.58336
XP_021993220.1	XP_022010616.1	1.3748	2.3563	0.58346
XP_021981407.1	XP_022029738.1	1.162	1.9912	0.58357
XP_022018622.1	XP_022022487.2	1.4897	2.5527	0.58358
XP_022006926.1	XP_022010295.2	0.8701	1.4904	0.5838
XP_022010101.1	XP_022010294.1	0.824	1.4112	0.5839
XP_022029739.1	XP_022037775.1	1.0417	1.7828	0.58431
XP_021973854.1	XP_022010294.1	1.1392	1.9496	0.58432
XP_021995116.1	XP_022029738.1	1.0829	1.853	0.5844
XP_022002420.1	XP_022029738.1	0.4632	0.7921	0.58477
XP_022010294.1	XP_022021912.1	1.1625	1.9878	0.58482
XP_022002415.1	XP_022006927.1	0.947	1.619	0.58493
XP_021993052.1	XP_022029738.1	0.8682	1.4842	0.58496
XP_021981626.1	XP_022002421.1	1.018	1.7396	0.58519
XP_021973316.1	XP_022002417.1	1.0329	1.7643	0.58544
XP_022006327.1	XP_035835830.1	1.2773	2.1815	0.58551
XP_021995116.1	XP_022002415.1	1.1818	2.0166	0.58604
XP_022010101.1	XP_022013182.1	1.3595	2.3195	0.58612
XP_021972810.1	XP_021993621.2	1.0586	1.8052	0.58642
XP_022002419.1	XP_022026963.1	1.0636	1.8134	0.58652
XP_021976022.1	XP_022006926.1	1.3161	2.2439	0.58652
XP_021981407.1	XP_022035384.1	1.2614	2.1503	0.58662
XP_021993621.2	XP_022002420.1	0.902	1.5374	0.5867
XP_022006927.1	XP_022008276.2	1.2443	2.1195	0.58707
XP_021972810.1	XP_022002420.1	0.5969	1.0166	0.58715
XP_021981407.1	XP_021993052.1	1.2725	2.1661	0.58746
XP_021988953.1	XP_022006327.1	1.2705	2.1623	0.58757
XP_021987457.1	XP_022000224.2	1.0221	1.7386	0.58789
XP_021972810.1	XP_021987458.1	1.2094	2.0567	0.58803
XP_021981388.1	XP_021993222.1	1.2827	2.181	0.58812
XP_021981407.1	XP_022035552.1	0.909	1.5449	0.58839
XP_021987457.1	XP_021993283.1	1.5277	2.5959	0.5885
XP_021993222.1	XP_022002418.1	1.0237	1.7393	0.58857
XP_022006926.1	XP_022018622.1	1.0759	1.8266	0.58902
XP_022002418.1	XP_022002420.1	0.1696	0.2879	0.58909
XP_021969020.1	XP_022022835.1	1.4416	2.4466	0.58923
XP_022010296.1	XP_035835830.1	0.729	1.2371	0.58928
XP_022002416.1	XP_022022835.1	1.0273	1.743	0.58939
XP_022002415.1	XP_022034230.1	0.9678	1.6419	0.58944
XP_021987458.1	XP_022033162.1	1.1696	1.9841	0.58949
XP_021984766.1	XP_022006926.1	1.2237	2.0756	0.58956
XP_021993551.1	XP_022004979.1	1.3394	2.2714	0.58968
XP_022010616.1	XP_022029739.1	0.8876	1.505	0.58977
XP_021969020.1	XP_021989735.1	1.2449	2.1107	0.5898
XP_021989736.1	XP_022010919.1	1.1293	1.9144	0.5899
XP_022010294.1	XP_022018622.1	1.054	1.7861	0.59011
XP_022013239.1	XP_022039074.2	1.3416	2.2729	0.59026
XP_022008275.1	XP_022013182.1	1.0713	1.8143	0.59048

XP_021973011.1	XP_022009213.1	1.0352	1.7522	0.5908
XP_021981407.1	XP_021987457.1	1.0939	1.8511	0.59095
XP_022010919.1	XP_022022487.2	1.4399	2.4363	0.59102
XP_021978842.1	XP_022004980.1	1.4954	2.5299	0.59109
XP_022002417.1	XP_022010291.1	0.6268	1.0603	0.59115
XP_021984766.1	XP_021989735.1	0.9659	1.6332	0.59142
XP_021995116.1	XP_022002416.1	1.2289	2.0776	0.5915
XP_021973316.1	XP_021995116.1	1.4981	2.5326	0.59153
XP_022006926.1	XP_022008276.2	1.0388	1.7557	0.59167
XP_021973011.1	XP_022002420.1	0.9033	1.5261	0.5919
XP_021985239.1	XP_021993220.1	1.0823	1.8263	0.59262
XP_021981407.1	XP_021987458.1	1.1386	1.9203	0.59293
XP_022006668.1	XP_022039085.1	1.1928	2.0108	0.5932
XP_021988953.1	XP_021999258.1	1.233	2.0784	0.59324
XP_021981395.1	XP_022035384.1	1.3271	2.2354	0.59367
XP_021981626.1	XP_022013182.1	1.2352	2.0805	0.5937
XP_022029739.1	XP_022039085.1	0.8033	1.353	0.59372
XP_021968895.1	XP_022002418.1	0.7687	1.2946	0.59377
XP_021993052.1	XP_035835830.1	0.9967	1.6785	0.5938
XP_021989736.1	XP_022010101.1	1.1087	1.8666	0.59397
XP_021981626.1	XP_022009213.1	1.2477	2.099	0.59443
XP_022004455.1	XP_022010292.1	0.5961	1.0028	0.59444
XP_021987458.1	XP_022008276.2	1.2929	2.1743	0.59463
XP_022010291.1	XP_022021913.1	1.3655	2.2947	0.59507
XP_022010291.1	XP_022021914.1	1.3655	2.2947	0.59507
XP_021968895.1	XP_021984981.2	1.0771	1.8094	0.59528
XP_021993222.1	XP_021993551.1	2.0768	3.4864	0.59569
XP_022034230.1	XP_035833070.1	2.0497	3.4372	0.59633
XP_022002416.1	XP_022013558.1	0.4775	0.8006	0.59643
XP_022010295.2	XP_035833070.1	1.3508	2.2633	0.59683
XP_021969020.1	XP_022029740.1	1.0558	1.7689	0.59687
XP_021986500.1	XP_022039074.2	1.2519	2.0973	0.59691
XP_022013183.1	XP_022015084.1	1.1848	1.9844	0.59706
XP_022013560.1	XP_022033162.1	0.84	1.4068	0.5971
XP_021992525.1	XP_035833070.1	1.5135	2.5347	0.59711
XP_022001254.1	XP_022004455.1	0.9631	1.6129	0.59712
XP_022010919.1	XP_022013183.1	1.2689	2.1249	0.59716
XP_021989737.1	XP_022010292.1	0.9454	1.5823	0.59748
XP_022037775.1	XP_035842399.1	1.3377	2.2381	0.59769
XP_022006926.1	XP_022013239.1	1.3204	2.2083	0.59793
XP_021999258.1	XP_022013558.1	1.0565	1.7661	0.59821
XP_021981626.1	XP_021993221.1	1.7268	2.8852	0.5985
XP_021993220.1	XP_022035552.1	1.4823	2.4763	0.59859
XP_022002420.1	XP_022029739.1	0.4423	0.7387	0.59875
XP_021978807.1	XP_021989735.1	1.144	1.9102	0.59889
XP_021981395.1	XP_021984981.2	0.8436	1.4081	0.59911
XP_021978842.1	XP_022021912.1	1.2413	2.0696	0.59978
XP_021973011.1	XP_021981407.1	1.0899	1.8171	0.5998
XP_022010101.1	XP_022029622.1	1.1113	1.8518	0.60012
XP_021995117.1	XP_022006927.1	1.1959	1.9926	0.60017
XP_021973011.1	XP_021987457.1	1.2772	2.1274	0.60036
XP_021984981.2	XP_022006327.1	1.0825	1.8027	0.60049
XP_021989734.1	XP_022029738.1	1.0681	1.7787	0.60049
XP_021993621.2	XP_022013182.1	1.2308	2.049	0.60068
XP_022010294.1	XP_022022487.2	0.9546	1.5889	0.60079
XP_022002416.1	XP_022006926.1	0.8572	1.4264	0.60095
XP_022006927.1	XP_022029739.1	1.0635	1.7696	0.60098
XP_022002421.1	XP_022035551.1	1.0876	1.8097	0.60098
XP_022002421.1	XP_022035552.1	1.0876	1.8097	0.60098
XP_022000224.2	XP_022033162.1	0.9217	1.533	0.60124
XP_022002421.1	XP_022010296.1	0.6258	1.0407	0.60133
XP_021989736.1	XP_022006668.1	1.1582	1.9258	0.60141
XP_021978842.1	XP_021989733.1	0.9278	1.5422	0.60161
XP_021978842.1	XP_022008276.2	1.2652	2.1018	0.60196
XP_021984766.1	XP_022004980.1	1.625	2.6994	0.60199
XP_021968895.1	XP_022000196.1	1.3062	2.1678	0.60255
XP_021978807.1	XP_021993221.1	1.2053	1.9997	0.60274
XP_021968895.1	XP_022014870.1	1.5807	2.6224	0.60277
XP_022002421.1	XP_022034230.1	1.0114	1.6778	0.60281
XP_022002416.1	XP_022004455.1	0.2289	0.3796	0.603
XP_022010296.1	XP_035842399.1	1.0355	1.7169	0.60312
XP_021985239.1	XP_021989733.1	1.0211	1.6912	0.60377
XP_021981395.1	XP_022000196.1	1.0997	1.8207	0.604
XP_021995117.1	XP_022013183.1	1.1929	1.9747	0.60409
XP_022004979.1	XP_022008275.1	0.8894	1.4719	0.60425
XP_022022835.1	XP_035835830.1	1.2172	2.0142	0.60431
XP_022009213.1	XP_035842399.1	1.6307	2.6977	0.60448
XP_021984766.1	XP_022029740.1	1.1468	1.8969	0.60457
XP_021993283.1	XP_022018622.1	1.3407	2.2176	0.60457
XP_022006926.1	XP_022029738.1	1.0361	1.7128	0.60492
XP_022002420.1	XP_022013183.1	1.1337	1.8741	0.60493
XP_022013182.1	XP_022033162.1	1.2743	2.1064	0.60497
XP_035838717.1	XP_035842399.1	1.6177	2.6734	0.60511
XP_022006668.1	XP_022022835.1	1.3121	2.1678	0.60527
XP_021993620.1	XP_022002420.1	0.941	1.554	0.60553
XP_022006327.1	XP_022021913.1	1.4345	2.3686	0.60563
XP_022006327.1	XP_022021914.1	1.4345	2.3686	0.60563
XP_021976022.1	XP_022010294.1	1.0924	1.8035	0.60571

XP_022002418.1	XP_022005608.1	0.8481	1.3999	0.60583
XP_021993283.1	XP_035835830.1	1.5378	2.534	0.60687
XP_022002419.1	XP_022008275.1	1.1092	1.8272	0.60705
XP_021999258.1	XP_022000224.2	1.0729	1.767	0.60719
XP_021988229.1	XP_022000196.1	1.2971	2.1361	0.60723
XP_021981395.1	XP_021993222.1	1.3835	2.2777	0.60741
XP_022002418.1	XP_022004455.1	0.2641	0.4346	0.60769
XP_021984981.2	XP_021995116.1	1.3409	2.2054	0.60801
XP_021993622.1	XP_022002419.1	1.1351	1.8667	0.60808
XP_022005608.1	XP_022039085.1	1.1545	1.8986	0.60808
XP_022002416.1	XP_022010291.1	0.5679	0.9339	0.6081
XP_022000196.1	XP_022002421.1	1.0978	1.8051	0.60817
XP_021968895.1	XP_021989735.1	1.1838	1.945	0.60864
XP_021973316.1	XP_021984981.2	1.2288	2.0171	0.60919
XP_022010291.1	XP_022039085.1	0.7261	1.1919	0.6092
XP_021968895.1	XP_022013182.1	1.1809	1.9383	0.60925
XP_021993222.1	XP_022013558.1	1.2516	2.0543	0.60926
XP_021981626.1	XP_022013560.1	1.1547	1.895	0.60934
XP_021981395.1	XP_022010291.1	0.9824	1.6122	0.60935
XP_021968895.1	XP_022022487.2	0.9869	1.6194	0.60942
XP_022013558.1	XP_022022835.1	0.9885	1.622	0.60943
XP_022000196.1	XP_022004455.1	1.1606	1.904	0.60956
XP_022006926.1	XP_022035551.1	1.26	2.0665	0.60973
XP_022006926.1	XP_022035552.1	1.26	2.0665	0.60973
XP_022002417.1	XP_022033162.1	0.6832	1.1205	0.60973
XP_021989735.1	XP_022002421.1	0.9489	1.5562	0.60975
XP_021993220.1	XP_022002416.1	0.9441	1.5479	0.60992
XP_021989737.1	XP_022002419.1	0.9117	1.4942	0.61016
XP_021984766.1	XP_021987457.1	1.112	1.8222	0.61025
XP_021984766.1	XP_021987458.1	1.112	1.8222	0.61025
XP_022010296.1	XP_035833070.1	1.4278	2.3394	0.61033
XP_022018622.1	XP_035835830.1	1.1117	1.8211	0.61046
XP_022002415.1	XP_022005608.1	0.8398	1.3755	0.61054
XP_021976022.1	XP_035833070.1	1.4269	2.3364	0.61073
XP_021981407.1	XP_022035551.1	0.9059	1.4829	0.6109
XP_021981626.1	XP_022010616.1	1.3046	2.1354	0.61094
XP_022026964.1	XP_022029739.1	1.0018	1.6397	0.61097
XP_021981626.1	XP_022013183.1	1.3058	2.1368	0.6111
XP_021993221.1	XP_022029739.1	1.3485	2.2066	0.61112
XP_021969020.1	XP_022029738.1	1.3386	2.1904	0.61112
XP_021987458.1	XP_021993622.1	1.3264	2.1699	0.61127
XP_022013560.1	XP_022029738.1	0.6528	1.0678	0.61135
XP_021978807.1	XP_022034230.1	1.1394	1.8637	0.61136
XP_022002418.1	XP_022010292.1	0.5421	0.886	0.61185
XP_021989735.1	XP_022029738.1	0.9895	1.6172	0.61186
XP_022006668.1	XP_022035551.1	1.355	2.2125	0.61243
XP_022006668.1	XP_022035552.1	1.355	2.2125	0.61243
XP_022006327.1	XP_022021911.1	1.3865	2.2632	0.61263
XP_021993220.1	XP_022002417.1	1.0227	1.6693	0.61265
XP_021993220.1	XP_022013560.1	1.0497	1.7111	0.61347
XP_022002418.1	XP_022006327.1	1.1197	1.8252	0.61347
XP_021992525.1	XP_022014870.1	1.0077	1.6426	0.61348
XP_021984981.2	XP_022006926.1	1.4408	2.3482	0.61358
XP_021987458.1	XP_022000224.2	1.0592	1.7261	0.61364
XP_022006668.1	XP_022018622.1	1.2586	2.0508	0.61371
XP_022008276.2	XP_035838717.1	1.2267	1.9979	0.61399
XP_022033162.1	XP_022039085.1	0.858	1.3974	0.614
XP_021981407.1	XP_022002421.1	1.0443	1.7007	0.61404
XP_021981626.1	XP_022018622.1	1.4996	2.4412	0.61429
XP_021984981.2	XP_022000196.1	1.2572	2.0463	0.61438
XP_021984766.1	XP_021995117.1	1.0527	1.7134	0.61439
XP_022002417.1	XP_022008276.2	1.1005	1.79	0.6148
XP_021987458.1	XP_021993283.1	1.5653	2.5459	0.61483
XP_022002417.1	XP_022002420.1	0.1508	0.2452	0.61501
XP_021989735.1	XP_022013560.1	0.9468	1.5387	0.61532
XP_022002417.1	XP_022002419.1	0.136	0.2209	0.61566
XP_021968895.1	XP_021992525.1	1.2303	1.9983	0.61567
XP_021989735.1	XP_022013239.1	1.4341	2.3291	0.61573
XP_021968895.1	XP_022029739.1	0.8347	1.3556	0.61574
XP_021981626.1	XP_035838717.1	1.1909	1.9335	0.61593
XP_022002415.1	XP_022022835.1	0.9933	1.6124	0.61604
XP_022010919.1	XP_022034230.1	1.3245	2.1497	0.61613
XP_021995117.1	XP_022002421.1	0.9285	1.5068	0.61621
XP_021973011.1	XP_021973854.1	1.4428	2.3407	0.6164
XP_021972810.1	XP_022006668.1	1.186	1.9237	0.61652
XP_021972810.1	XP_022029622.1	1.1744	1.9042	0.61674
XP_022010291.1	XP_022021911.1	1.3518	2.1912	0.61692
XP_022004980.1	XP_022022487.2	1.7396	2.8197	0.61695
XP_021993620.1	XP_035842399.1	1.5125	2.4514	0.61699
XP_022004980.1	XP_035838717.1	1.1123	1.8027	0.61702
XP_022002421.1	XP_022013558.1	0.5573	0.9032	0.61703
XP_022000224.2	XP_022010292.1	0.8339	1.3512	0.61716
XP_021989737.1	XP_022010296.1	0.9908	1.6045	0.61751
XP_022014870.1	XP_022021911.1	1.1739	1.9003	0.61774
XP_021988229.1	XP_022029622.1	1.3943	2.2561	0.61801
XP_021973316.1	XP_022002416.1	1.0305	1.6673	0.61807
XP_021999258.1	XP_022002417.1	1.1021	1.7831	0.61808
XP_021969020.1	XP_021989736.1	1.2151	1.9656	0.61818

XP_021993620.1	XP_022001254.1	1.1918	1.9279	0.61819
XP_021993221.1	XP_022022487.2	1.4581	2.3586	0.61821
XP_021988953.1	XP_021989737.1	1	1.6175	0.61824
XP_022000196.1	XP_035833070.1	1.59	2.5716	0.61829
XP_021968895.1	XP_022009213.1	1.3836	2.2356	0.61889
XP_022002418.1	XP_022010296.1	0.558	0.9014	0.61904
XP_021993052.1	XP_022033162.1	1.0154	1.6402	0.61907
XP_021985239.1	XP_021993621.2	1.1786	1.9037	0.61911
XP_022010296.1	XP_022039085.1	0.7085	1.1442	0.61921
XP_021993222.1	XP_022013182.1	1.5475	2.4969	0.61977
XP_021978807.1	XP_021993222.1	1.332	2.149	0.61982
XP_021985239.1	XP_022013182.1	1.189	1.9179	0.61995
XP_021981407.1	XP_022006668.1	1.1724	1.891	0.61999
XP_021987457.1	XP_022002417.1	1.1855	1.9109	0.62039
XP_021989733.1	XP_022002417.1	1.0118	1.6307	0.62047
XP_021972810.1	XP_021984981.2	1.0319	1.663	0.62051
XP_021987457.1	XP_022004980.1	1.3519	2.1773	0.62091
XP_021981626.1	XP_022010295.2	0.9938	1.6005	0.62093
XP_021981407.1	XP_022033162.1	1.1653	1.8765	0.621
XP_022000224.2	XP_022029738.1	0.9226	1.4856	0.62103
XP_022002421.1	XP_022026963.1	1.0718	1.7257	0.62108
XP_021988229.1	XP_022002419.1	0.881	1.418	0.6213
XP_021978807.1	XP_022004979.1	1.2724	2.0475	0.62144
XP_022002420.1	XP_022010291.1	0.5565	0.8952	0.62165
XP_022010616.1	XP_022010919.1	1.0418	1.6754	0.62182
XP_021989734.1	XP_022002421.1	1.0211	1.6421	0.62183
XP_021993621.2	XP_022008275.1	1.1547	1.8567	0.62191
XP_021969020.1	XP_021993222.1	1.3531	2.1756	0.62194
XP_021993052.1	XP_022034230.1	1.184	1.9037	0.62195
XP_022004455.1	XP_022010295.2	0.6098	0.9804	0.62199
XP_022001254.1	XP_022029739.1	1.2271	1.9727	0.62204
XP_021973011.1	XP_022010295.2	0.9745	1.5661	0.62225
XP_022002417.1	XP_022004980.1	1.1885	1.9096	0.62238
XP_022010919.1	XP_022037775.1	1.2427	1.9961	0.62256
XP_022002415.1	XP_022010292.1	0.5252	0.8434	0.62272
XP_022006668.1	XP_022037775.1	1.0528	1.6905	0.62277
XP_021981407.1	XP_021993621.2	1.178	1.8913	0.62285
XP_022013182.1	XP_022018622.1	1.5219	2.4431	0.62294
XP_022022487.2	XP_022029738.1	1.0795	1.7329	0.62294
XP_021981395.1	XP_022039085.1	1.15	1.8459	0.623
XP_021993283.1	XP_035842399.1	1.4226	2.2834	0.62302
XP_021999258.1	XP_035835830.1	1.193	1.9136	0.62343
XP_022010616.1	XP_022039074.2	1.3351	2.1415	0.62344
XP_021986500.1	XP_022029739.1	0.8674	1.3912	0.62349
XP_021973011.1	XP_022022835.1	1.0282	1.6479	0.62395
XP_021987458.1	XP_022002417.1	1.2015	1.9256	0.62396
XP_021984981.2	XP_022014870.1	0.7857	1.2585	0.62431
XP_022002419.1	XP_022013558.1	0.5634	0.9024	0.62434
XP_021993622.1	XP_022002417.1	1.1619	1.8609	0.62438
XP_022010291.1	XP_022034230.1	0.9469	1.5155	0.62481
XP_022022835.1	XP_022029740.1	1.016	1.6259	0.62488
XP_021981388.1	XP_022026963.1	1.3912	2.2256	0.62509
XP_021973011.1	XP_021981626.1	1.2167	1.946	0.62523
XP_021992525.1	XP_022033162.1	1.2166	1.945	0.6255
XP_021999258.1	XP_022008276.2	1.5393	2.4599	0.62576
XP_022002417.1	XP_022014870.1	1.0399	1.6616	0.62584
XP_021978842.1	XP_021981407.1	1.0283	1.6426	0.62602
XP_022021911.1	XP_022033162.1	1.2965	2.0709	0.62606
XP_022021913.1	XP_022033162.1	1.2965	2.0709	0.62606
XP_022021914.1	XP_022033162.1	1.2965	2.0709	0.62606
XP_022002415.1	XP_035835830.1	0.4889	0.7808	0.62615
XP_021985239.1	XP_022013183.1	1.1373	1.816	0.62627
XP_021973011.1	XP_022006927.1	1.1653	1.86	0.62651
XP_021989733.1	XP_022010919.1	1.1836	1.8892	0.62651
XP_022010294.1	XP_022013558.1	0.2806	0.4475	0.62704
XP_021989733.1	XP_022002415.1	0.995	1.5867	0.62709
XP_021981388.1	XP_021981395.1	0.0604	0.0963	0.62721
XP_022010295.2	XP_022013558.1	0.1459	0.2326	0.62726
XP_021981407.1	XP_022034230.1	1.2242	1.9513	0.62738
XP_022010291.1	XP_022010294.1	0.6007	0.9574	0.62743
XP_021976022.1	XP_021981407.1	1.2261	1.9538	0.62755
XP_021969020.1	XP_022013560.1	1.0814	1.7232	0.62755
XP_022002419.1	XP_022035551.1	0.9876	1.5737	0.62757
XP_022002419.1	XP_022035552.1	0.9876	1.5737	0.62757
XP_022022487.2	XP_035842399.1	1.5927	2.5369	0.62781
XP_021973011.1	XP_022002419.1	0.9954	1.5853	0.62789
XP_022018622.1	XP_022022835.1	1.3066	2.0799	0.6282
XP_022002415.1	XP_022002419.1	0.1413	0.2249	0.62828
XP_021989737.1	XP_022029738.1	0.9933	1.5807	0.62839
XP_022005608.1	XP_022029739.1	0.9426	1.4994	0.62865
XP_021993052.1	XP_022021911.1	1.0913	1.735	0.62899
XP_021993052.1	XP_022021913.1	1.0913	1.735	0.62899
XP_021993052.1	XP_022021914.1	1.0913	1.735	0.62899
XP_021988953.1	XP_022026964.1	1.2235	1.945	0.62905
XP_021999258.1	XP_022002419.1	1.0144	1.6124	0.62912
XP_022002418.1	XP_035835830.1	0.4821	0.7662	0.62921
XP_022001254.1	XP_022002416.1	1.0091	1.6036	0.62927
XP_021987457.1	XP_021988229.1	1.0371	1.6479	0.62935

XP_021989734.1	XP_022035552.1	0.9525	1.5133	0.62942
XP_021992525.1	XP_022010101.1	1.5208	2.4159	0.6295
XP_021985239.1	XP_022034230.1	1.2825	2.0367	0.6297
XP_021981626.1	XP_021987457.1	1.1211	1.7798	0.6299
XP_021973316.1	XP_022010616.1	1.2635	2.0056	0.62999
XP_022001254.1	XP_022002418.1	1.0315	1.6367	0.63023
XP_022002421.1	XP_022008275.1	1.0784	1.7111	0.63024
XP_022002415.1	XP_022004455.1	0.2187	0.347	0.63026
XP_021981395.1	XP_022014870.1	1.0148	1.6097	0.63043
XP_022013558.1	XP_022039074.2	0.7117	1.1289	0.63044
XP_021995116.1	XP_022033162.1	1.6207	2.5705	0.6305
XP_021988229.1	XP_022014870.1	1.3353	2.1169	0.63078
XP_022004455.1	XP_022013239.1	1.0716	1.6988	0.6308
XP_021984766.1	XP_021985239.1	1.0935	1.7331	0.63095
XP_022006668.1	XP_022010291.1	1.0809	1.7128	0.63107
XP_022022487.2	XP_022039085.1	1.0851	1.7191	0.6312
XP_022002419.1	XP_022035384.1	1.334	2.1133	0.63124
XP_021999258.1	XP_022039074.2	1.3043	2.0662	0.63126
XP_021989736.1	XP_022000196.1	1.0828	1.7146	0.63152
XP_021984981.2	XP_035842399.1	1.2053	1.908	0.63171
XP_021993283.1	XP_022033162.1	1.5813	2.503	0.63176
XP_021973011.1	XP_022010101.1	1.0872	1.7206	0.63187
XP_021993621.2	XP_022029739.1	1.0927	1.7288	0.63206
XP_021973316.1	XP_022000196.1	1.2874	2.0363	0.63223
XP_022010616.1	XP_022021911.1	1.0522	1.6641	0.63229
XP_022010616.1	XP_022021913.1	1.0522	1.6641	0.63229
XP_022010616.1	XP_022021914.1	1.0522	1.6641	0.63229
XP_021989737.1	XP_022026964.1	1.0935	1.7282	0.63274
XP_021968895.1	XP_021976022.1	1.1692	1.8478	0.63275
XP_022008275.1	XP_035838717.1	1.0933	1.7277	0.63281
XP_021968895.1	XP_022039085.1	0.7875	1.2444	0.63284
XP_021988229.1	XP_022002415.1	0.791	1.2497	0.63295
XP_021978842.1	XP_021992525.1	1.3269	2.0961	0.63303
XP_021989737.1	XP_022039074.2	1.18	1.8637	0.63315
XP_021989736.1	XP_022013239.1	1.3863	2.1892	0.63325
XP_022013182.1	XP_022026964.1	1.1975	1.89	0.6336
XP_022013183.1	XP_022026964.1	1.1975	1.89	0.6336
XP_021995116.1	XP_022022487.2	1.3485	2.127	0.63399
XP_021993620.1	XP_022002415.1	0.9091	1.4337	0.63409
XP_022004455.1	XP_022006926.1	1.019	1.6068	0.63418
XP_022006927.1	XP_022013560.1	0.8533	1.3452	0.63433
XP_022039074.2	XP_022039085.1	0.8645	1.3626	0.63445
XP_021988229.1	XP_022002416.1	0.8224	1.2961	0.63452
XP_021978842.1	XP_021981388.1	0.9659	1.5222	0.63454
XP_022021912.1	XP_022029739.1	1.053	1.6594	0.63457
XP_022006926.1	XP_022039085.1	0.9412	1.4831	0.63462
XP_022029740.1	XP_022033162.1	0.6839	1.0776	0.63465
XP_021981388.1	XP_021993220.1	1.1895	1.872	0.63542
XP_022018622.1	XP_022035384.1	1.2159	1.9131	0.63557
XP_021989733.1	XP_022014870.1	0.9992	1.5721	0.63558
XP_021995117.1	XP_035838717.1	0.9952	1.5657	0.63563
XP_021968895.1	XP_035833070.1	1.2091	1.9018	0.63577
XP_021984766.1	XP_022002416.1	1.0716	1.6849	0.636
XP_022002416.1	XP_022002420.1	0.1474	0.2317	0.63617
XP_022013560.1	XP_022039085.1	0.7468	1.1736	0.63633
XP_022006926.1	XP_022029740.1	0.8991	1.4117	0.63689
XP_021968895.1	XP_022013239.1	1.2752	2.0016	0.63709
XP_022002415.1	XP_022010296.1	0.5444	0.8545	0.6371
XP_021989737.1	XP_022033162.1	1.2299	1.9303	0.63715
XP_022010292.1	XP_035838717.1	0.9505	1.4914	0.63732
XP_021981407.1	XP_022026963.1	1.4369	2.2534	0.63766
XP_022002419.1	XP_022010291.1	0.5133	0.8049	0.63772
XP_021972810.1	XP_022010296.1	0.7275	1.1407	0.63777
XP_021976022.1	XP_021988953.1	1.8441	2.8913	0.63781
XP_021973316.1	XP_021988953.1	1.4774	2.316	0.63791
XP_021988953.1	XP_021989735.1	0.9728	1.5246	0.63807
XP_021985239.1	XP_022039085.1	0.7849	1.23	0.63813
XP_021986500.1	XP_021988953.1	1.0454	1.6377	0.63833
XP_022010292.1	XP_022013183.1	1.2737	1.9949	0.63848
XP_022004979.1	XP_022013183.1	1.2063	1.8888	0.63866
XP_022002419.1	XP_022004455.1	0.1897	0.297	0.63872
XP_022006927.1	XP_035842399.1	1.3208	2.0673	0.6389
XP_022004455.1	XP_022009213.1	0.9894	1.5484	0.63898
XP_021981626.1	XP_021987458.1	1.0927	1.7099	0.63904
XP_021968895.1	XP_021999258.1	1.2963	2.0279	0.63923
XP_021989734.1	XP_022010919.1	1.135	1.7753	0.63933
XP_022002417.1	XP_035835830.1	0.4917	0.769	0.6394
XP_021989736.1	XP_022029738.1	1.1178	1.7464	0.64006
XP_021995117.1	XP_022026963.1	1.003	1.5668	0.64016
XP_021978842.1	XP_021989735.1	0.9742	1.5218	0.64016
XP_022021911.1	XP_022029738.1	1.0985	1.7156	0.6403
XP_022021913.1	XP_022029738.1	1.0985	1.7156	0.6403
XP_022021914.1	XP_022029738.1	1.0985	1.7156	0.6403
XP_022010919.1	XP_022029622.1	1.1949	1.8653	0.64059
XP_022004455.1	XP_022010296.1	0.6386	0.9968	0.64065
XP_022006926.1	XP_022013560.1	0.8231	1.2846	0.64074
XP_021987457.1	XP_021993622.1	1.3375	2.0871	0.64084
XP_021987457.1	XP_022029738.1	1.2659	1.9748	0.64103

XP_021999258.1	XP_022010295.2	1.0397	1.6218	0.64108
XP_021968895.1	XP_022000224.2	1.2006	1.8722	0.64128
XP_022002415.1	XP_022033162.1	0.6835	1.0656	0.64142
XP_021973011.1	XP_035833070.1	1.4167	2.2083	0.64153
XP_021973854.1	XP_021992525.1	1.4617	2.2784	0.64155
XP_021969020.1	XP_021989737.1	1.3643	2.1249	0.64205
XP_021993220.1	XP_021995117.1	1.0441	1.6259	0.64217
XP_021989735.1	XP_022033162.1	1.2163	1.8938	0.64225
XP_021993220.1	XP_022035551.1	1.4647	2.2791	0.64267
XP_021989734.1	XP_022006327.1	1.2975	2.0181	0.64293
XP_022014870.1	XP_022029738.1	0.8942	1.3904	0.64312
XP_022010294.1	XP_022039085.1	0.7303	1.1355	0.64315
XP_022013558.1	XP_035833070.1	1.3228	2.0565	0.64323
XP_022002416.1	XP_022033162.1	0.6907	1.0736	0.64335
XP_021972810.1	XP_022034230.1	1.1104	1.7259	0.64337
XP_022034230.1	XP_022039074.2	1.1889	1.8479	0.64338
XP_021969020.1	XP_022013558.1	1.0432	1.6206	0.64371
XP_021981626.1	XP_022022835.1	1.2034	1.8682	0.64415
XP_021969020.1	XP_035833070.1	2.0326	3.1552	0.64421
XP_021978807.1	XP_021993620.1	1.2411	1.9262	0.64433
XP_021973316.1	XP_021989737.1	1.3598	2.1101	0.64442
XP_021968895.1	XP_021981388.1	1.1473	1.7795	0.64473
XP_022002421.1	XP_022010295.2	0.5955	0.9235	0.64483
XP_021984766.1	XP_022029738.1	1.1648	1.8059	0.645
XP_022006926.1	XP_022013558.1	0.8484	1.3152	0.64507
XP_021973011.1	XP_022002416.1	0.9503	1.4731	0.6451
XP_021968895.1	XP_022002419.1	0.7616	1.1805	0.64515
XP_021987458.1	XP_022021913.1	1.2188	1.8882	0.64548
XP_021987458.1	XP_022021914.1	1.2188	1.8882	0.64548
XP_022000196.1	XP_022008276.2	1.8991	2.9421	0.64549
XP_021981626.1	XP_022029739.1	1.1859	1.8372	0.64549
XP_021969020.1	XP_022000224.2	1.2866	1.9931	0.64553
XP_021988229.1	XP_022029738.1	0.8192	1.2683	0.6459
XP_021993551.1	XP_022021912.1	1.4749	2.2834	0.64592
XP_021968895.1	XP_022002415.1	0.7744	1.1985	0.64614
XP_021993622.1	XP_022026964.1	1.0502	1.625	0.64628
XP_021989737.1	XP_022006327.1	1.2185	1.8853	0.64632
XP_021981407.1	XP_022014870.1	0.902	1.3956	0.64632
XP_021988229.1	XP_022008276.2	1.2983	2.0086	0.64637
XP_021989734.1	XP_022035551.1	0.9477	1.4659	0.6465
XP_021993551.1	XP_022022487.2	1.3302	2.0563	0.64689
XP_022010294.1	XP_035842399.1	1.0556	1.6317	0.64693
XP_022004455.1	XP_022004980.1	1.1945	1.8461	0.64704
XP_022005608.1	XP_022039074.2	1.1957	1.8477	0.64713
XP_022006926.1	XP_022010294.1	0.8052	1.2441	0.64721
XP_022001254.1	XP_022002417.1	1.0562	1.6318	0.64726
XP_035835830.1	XP_035842399.1	1.2973	2.004	0.64736
XP_021989735.1	XP_022010919.1	1.1796	1.8218	0.64749
XP_021981626.1	XP_022029738.1	1.1966	1.848	0.64751
XP_021989733.1	XP_022005608.1	1.0415	1.6084	0.64754
XP_021976022.1	XP_022015084.1	1.2851	1.9844	0.6476
XP_021995117.1	XP_022029738.1	1.0847	1.6742	0.64789
XP_022002417.1	XP_022021911.1	1.1199	1.7284	0.64794
XP_021978807.1	XP_021989734.1	1.173	1.8102	0.64799
XP_021973011.1	XP_022002418.1	0.9399	1.4504	0.64803
XP_021981626.1	XP_022033162.1	1.1212	1.7299	0.64813
XP_022002415.1	XP_022014870.1	0.9342	1.441	0.6483
XP_021989733.1	XP_022004980.1	1.0223	1.5764	0.6485
XP_021993222.1	XP_022029740.1	1.1311	1.7436	0.64872
XP_021987457.1	XP_022013182.1	1.1717	1.8059	0.64882
XP_021989736.1	XP_035842399.1	1.6732	2.5784	0.64893
XP_021988953.1	XP_022021912.1	1.3166	2.0287	0.64899
XP_021993620.1	XP_022002419.1	0.9738	1.4995	0.64942
XP_022010292.1	XP_022010294.1	0.3095	0.4765	0.64953
XP_021986500.1	XP_022033162.1	1.3157	2.0256	0.64954
XP_021988229.1	XP_022006668.1	1.102	1.6963	0.64965
XP_021978842.1	XP_021984981.2	1.1521	1.7727	0.64991
XP_021973316.1	XP_022022487.2	1.4882	2.2897	0.64995
XP_022000196.1	XP_022026964.1	1.0723	1.6479	0.65071
XP_021992525.1	XP_022026964.1	1.2457	1.9138	0.6509
XP_021984766.1	XP_022006327.1	1.3408	2.0599	0.65091
XP_021968895.1	XP_035835830.1	0.7953	1.2215	0.65108
XP_022010919.1	XP_022029739.1	0.9805	1.5059	0.65111
XP_022006327.1	XP_035842399.1	1.4676	2.253	0.6514
XP_021989736.1	XP_022034230.1	1.1444	1.7557	0.65182
XP_021993622.1	XP_022000196.1	1.5739	2.4142	0.65193
XP_021989736.1	XP_022006926.1	1.2742	1.9543	0.652
XP_022006668.1	XP_022021911.1	1.1586	1.7767	0.65211
XP_022006668.1	XP_022021913.1	1.1586	1.7767	0.65211
XP_022006668.1	XP_022021914.1	1.1586	1.7767	0.65211
XP_021981395.1	XP_022008276.2	1.434	2.1987	0.6522
XP_021995117.1	XP_035833070.1	1.3605	2.0857	0.6523
XP_021973011.1	XP_022010616.1	1.1287	1.7302	0.65235
XP_022002417.1	XP_022010919.1	0.9602	1.4716	0.65249
XP_021995117.1	XP_022026964.1	0.9108	1.3956	0.65262
XP_021987457.1	XP_022001254.1	1.2566	1.9252	0.65271
XP_021973855.1	XP_021992525.1	1.7657	2.7034	0.65314
XP_021981626.1	XP_021989733.1	1.2067	1.8474	0.65319

XP_021995117.1	XP_022002420.1	0.9059	1.3868	0.65323
XP_021973011.1	XP_021989737.1	1.0433	1.5968	0.65337
XP_021988953.1	XP_022002421.1	1.1204	1.7145	0.65348
XP_021993220.1	XP_022002418.1	0.9692	1.4831	0.6535
XP_022002420.1	XP_022010294.1	0.5634	0.8621	0.65352
XP_022004455.1	XP_022008275.1	1.1092	1.6969	0.65366
XP_022002419.1	XP_022010292.1	0.5798	0.887	0.65366
XP_021995116.1	XP_022002417.1	1.3092	2.0023	0.65385
XP_021981388.1	XP_021995116.1	1.2511	1.9134	0.65386
XP_021987457.1	XP_022010291.1	1.1616	1.7764	0.65391
XP_022002420.1	XP_022026964.1	0.8817	1.3482	0.65398
XP_021992525.1	XP_022029739.1	1.5593	2.3835	0.65421
XP_021999258.1	XP_022029739.1	1.0863	1.6604	0.65424
XP_022002416.1	XP_022026963.1	0.9784	1.495	0.65445
XP_022002417.1	XP_022026963.1	0.9784	1.495	0.65445
XP_021987458.1	XP_022010296.1	1.2507	1.9095	0.65499
XP_022002416.1	XP_022002419.1	0.135	0.2061	0.65502
XP_021986500.1	XP_021989737.1	0.9461	1.444	0.65519
XP_021993220.1	XP_022008276.2	1.3566	2.0701	0.65533
XP_021988229.1	XP_022006327.1	1.503	2.2924	0.65564
XP_021989734.1	XP_022029740.1	1.1623	1.7727	0.65567
XP_022002417.1	XP_022022835.1	1.0625	1.6204	0.6557
XP_022002418.1	XP_022022835.1	1.0087	1.5382	0.65577
XP_021993220.1	XP_022002419.1	0.9603	1.464	0.65594
XP_022013560.1	XP_022034230.1	0.9923	1.5127	0.65598
XP_021973854.1	XP_021989734.1	1.248	1.9018	0.65622
XP_022006927.1	XP_022010291.1	0.9819	1.4962	0.65626
XP_022006927.1	XP_022014870.1	1.1742	1.7879	0.65675
XP_021981407.1	XP_022010291.1	0.9636	1.4667	0.65699
XP_021973011.1	XP_022029739.1	0.9704	1.4769	0.65705
XP_021976022.1	XP_021993620.1	1.4877	2.2642	0.65705
XP_021988229.1	XP_022039085.1	0.8734	1.3291	0.65714
XP_022000224.2	XP_022002420.1	0.8413	1.2801	0.65721
XP_021976022.1	XP_021995116.1	1.3955	2.1233	0.65723
XP_021987458.1	XP_022035552.1	1.3218	2.0111	0.65725
XP_021989734.1	XP_022026964.1	1.0782	1.6401	0.6574
XP_022013183.1	XP_022018622.1	1.5117	2.2995	0.6574
XP_022002415.1	XP_022010291.1	0.5653	0.8597	0.65755
XP_022008275.1	XP_022026963.1	1.0167	1.5461	0.65759
XP_022010616.1	XP_022034230.1	0.8987	1.3665	0.65767
XP_022008276.2	XP_022029622.1	1.4212	2.1601	0.65793
XP_022013560.1	XP_022018622.1	1.1563	1.7572	0.65804
XP_021985239.1	XP_021995116.1	1.335	2.0285	0.65812
XP_021981626.1	XP_021992525.1	1.3508	2.0525	0.65812
XP_021993222.1	XP_022014870.1	1.581	2.4009	0.6585
XP_021981626.1	XP_022001254.1	1.2678	1.9252	0.65853
XP_022004455.1	XP_022022487.2	0.986	1.4971	0.65861
XP_021972810.1	XP_022022487.2	1.0334	1.5689	0.65868
XP_022002415.1	XP_022008276.2	1.0894	1.6537	0.65877
XP_021995116.1	XP_022014870.1	1.5159	2.301	0.6588
XP_021988953.1	XP_021989736.1	1.1564	1.7552	0.65884
XP_022008276.2	XP_022014870.1	1.2863	1.952	0.65897
XP_022002415.1	XP_022004980.1	1.2153	1.8442	0.65898
XP_021987457.1	XP_022035552.1	1.3086	1.985	0.65924
XP_021986500.1	XP_022004979.1	1.1691	1.7732	0.65932
XP_022002415.1	XP_022006926.1	0.8649	1.3118	0.65932
XP_022000196.1	XP_022002419.1	1.1765	1.7839	0.65951
XP_021973316.1	XP_035842399.1	1.5546	2.3567	0.65965
XP_021993551.1	XP_022035552.1	1.6794	2.5451	0.65986
XP_021973854.1	XP_022037775.1	1.4679	2.2244	0.65991
XP_021987458.1	XP_022029738.1	1.294	1.9608	0.65993
XP_021985239.1	XP_021992525.1	1.1758	1.7811	0.66015
XP_021984981.2	XP_022034230.1	1.0805	1.6361	0.66041
XP_021993220.1	XP_022010294.1	1.1311	1.7125	0.6605
XP_021988953.1	XP_022002418.1	1.0367	1.5695	0.66053
XP_021976022.1	XP_021981388.1	1.1085	1.6769	0.66104
XP_022004455.1	XP_035842399.1	1.0053	1.5207	0.66108
XP_021987458.1	XP_022000196.1	1.4606	2.2091	0.66117
XP_021993221.1	XP_022029622.1	1.3381	2.0224	0.66164
XP_021968895.1	XP_022039074.2	0.8147	1.2313	0.66166
XP_021989737.1	XP_022002418.1	0.9491	1.4337	0.66199
XP_021981395.1	XP_022005608.1	1.1444	1.7285	0.66208
XP_021989733.1	XP_022008276.2	1.2729	1.9222	0.66221
XP_021995117.1	XP_022021912.1	1.2024	1.8153	0.66237
XP_021986500.1	XP_022010616.1	0.7536	1.1377	0.66239
XP_021993052.1	XP_022002419.1	0.8487	1.281	0.66253
XP_021987458.1	XP_022035551.1	1.3384	2.02	0.66257
XP_022002420.1	XP_022010292.1	0.5481	0.8271	0.66268
XP_021976022.1	XP_022013560.1	1.1244	1.6966	0.66274
XP_022002418.1	XP_022006926.1	0.8375	1.2636	0.66279
XP_022000196.1	XP_022029622.1	1.352	2.0396	0.66288
XP_021993620.1	XP_022002421.1	0.9731	1.4673	0.66319
XP_022006327.1	XP_022029622.1	1.4948	2.2532	0.66341
XP_021972810.1	XP_021973011.1	1.1217	1.6905	0.66353
XP_022006327.1	XP_022033162.1	1.3525	2.0374	0.66384
XP_022002420.1	XP_022013558.1	0.5362	0.8077	0.66386
XP_021973011.1	XP_022001254.1	1.1543	1.7384	0.664
XP_022002418.1	XP_022008276.2	1.0659	1.6041	0.66448

XP_021987458.1	XP_022021911.1	1.1882	1.7879	0.66458
XP_022026964.1	XP_035835830.1	1.1873	1.7859	0.66482
XP_021993283.1	XP_022029622.1	1.224	1.8411	0.66482
XP_022013239.1	XP_035833070.1	1.4119	2.1233	0.66496
XP_021993551.1	XP_022002421.1	1.1256	1.6923	0.66513
XP_022002416.1	XP_022014870.1	1.0046	1.5103	0.66517
XP_022026963.1	XP_035838717.1	1.4179	2.131	0.66537
XP_022010292.1	XP_022013560.1	0.3215	0.483	0.66563
XP_021993621.2	XP_022021912.1	1.2719	1.9102	0.66585
XP_021987457.1	XP_022002420.1	1.175	1.7643	0.66599
XP_021987458.1	XP_022010291.1	1.1788	1.7696	0.66614
XP_022010616.1	XP_022029738.1	0.9237	1.3865	0.66621
XP_021988229.1	XP_022002417.1	0.8332	1.2498	0.66667
XP_021993220.1	XP_022010296.1	1.0568	1.5851	0.66671
XP_021973316.1	XP_022002415.1	1.0737	1.6102	0.66681
XP_021989736.1	XP_022002421.1	0.9608	1.4406	0.66694
XP_021993283.1	XP_022008275.1	1.3579	2.0359	0.66698
XP_021989735.1	XP_022014870.1	0.9444	1.4153	0.66728
XP_022010291.1	XP_022018622.1	1.3438	2.0132	0.66749
XP_021989737.1	XP_022013560.1	0.9898	1.4824	0.6677
XP_021988953.1	XP_021989734.1	0.9668	1.4475	0.66791
XP_022002416.1	XP_022010919.1	0.9424	1.4103	0.66823
XP_021992525.1	XP_022037775.1	1.2392	1.8544	0.66825
XP_021989736.1	XP_022035552.1	0.9459	1.4153	0.66834
XP_022013182.1	XP_022029738.1	1.302	1.9472	0.66865
XP_021984981.2	XP_022002421.1	1.2478	1.8654	0.66892
XP_021993052.1	XP_022010291.1	1.014	1.5155	0.66909
XP_022010296.1	XP_022022487.2	0.8499	1.2695	0.66948
XP_021985239.1	XP_022000224.2	1.1035	1.6479	0.66964
XP_021973855.1	XP_021988953.1	1.8878	2.8186	0.66977
XP_022039074.2	XP_035833070.1	1.2177	1.8177	0.66991
XP_022002416.1	XP_022008276.2	1.1043	1.6479	0.67013
XP_021981407.1	XP_022026964.1	1.0755	1.6044	0.67034
XP_021972810.1	XP_022039074.2	0.7736	1.1538	0.67048
XP_022008276.2	XP_035842399.1	1.6062	2.3954	0.67054
XP_021981626.1	XP_021989737.1	1.2059	1.7984	0.67054
XP_021992525.1	XP_022013239.1	1.5207	2.2676	0.67062
XP_021987458.1	XP_022002420.1	1.1924	1.7778	0.67072
XP_022006926.1	XP_022010292.1	0.8767	1.3071	0.67072
XP_021978807.1	XP_022039085.1	1.0628	1.5844	0.67079
XP_021993622.1	XP_022006926.1	1.7801	2.6534	0.67088
XP_021973855.1	XP_022004980.1	1.6167	2.4096	0.67094
XP_021973011.1	XP_022006327.1	1.2327	1.8368	0.67111
XP_021968895.1	XP_022013183.1	1.2266	1.8277	0.67112
XP_021989737.1	XP_022002420.1	0.9275	1.3816	0.67132
XP_021995117.1	XP_022022487.2	1.4404	2.1445	0.67167
XP_022004980.1	XP_022029740.1	1.0587	1.5758	0.67185
XP_021993220.1	XP_022002415.1	0.9605	1.4293	0.67201
XP_021976022.1	XP_021981395.1	1.0515	1.5639	0.67236
XP_022000224.2	XP_022029739.1	0.9619	1.4304	0.67247
XP_021987457.1	XP_022013183.1	1.184	1.7605	0.67254
XP_022029738.1	XP_035835830.1	0.6336	0.9421	0.67254
XP_021976022.1	XP_021989736.1	1.1337	1.6847	0.67294
XP_021993220.1	XP_022000224.2	1.5916	2.3646	0.67309
XP_022004980.1	XP_022022835.1	1.653	2.455	0.67332
XP_021993222.1	XP_022026963.1	1.384	2.0553	0.67338
XP_021968895.1	XP_021972810.1	0.8637	1.2821	0.67366
XP_021973316.1	XP_022002418.1	1.0734	1.5933	0.6737
XP_021993052.1	XP_021993220.1	1.1466	1.7019	0.67372
XP_022010296.1	XP_022013183.1	1.2608	1.871	0.67386
XP_022002418.1	XP_022010291.1	0.5844	0.8672	0.67389
XP_021993620.1	XP_022002417.1	0.9417	1.3973	0.67394
XP_022010295.2	XP_022010919.1	0.968	1.4362	0.674
XP_021995117.1	XP_022021911.1	1.1283	1.674	0.67401
XP_021995117.1	XP_022021913.1	1.1283	1.674	0.67401
XP_021995117.1	XP_022021914.1	1.1283	1.674	0.67401
XP_021973316.1	XP_022009213.1	1.2458	1.8476	0.67428
XP_021973011.1	XP_022015084.1	1.2334	1.8292	0.67428
XP_021993221.1	XP_022002421.1	1.0616	1.5743	0.67433
XP_021993621.2	XP_022029738.1	1.0647	1.5788	0.67437
XP_022002415.1	XP_022006327.1	1.1588	1.7174	0.67474
XP_021993052.1	XP_022029739.1	0.8447	1.2513	0.67506
XP_021993283.1	XP_022021913.1	1.598	2.3668	0.67517
XP_021993283.1	XP_022021914.1	1.598	2.3668	0.67517
XP_022013558.1	XP_022035384.1	0.9829	1.4551	0.67549
XP_021981395.1	XP_021981626.1	1.1359	1.6814	0.67557
XP_022002418.1	XP_022033162.1	0.6894	1.0201	0.67582
XP_021973316.1	XP_022026963.1	1.4143	2.0927	0.67583
XP_021985239.1	XP_022008275.1	1.2965	1.9179	0.676
XP_021985239.1	XP_021989734.1	1.0593	1.5668	0.67609
XP_022010292.1	XP_035833070.1	1.4729	2.1784	0.67614
XP_022004980.1	XP_022010294.1	1.0777	1.5937	0.67623
XP_021989737.1	XP_035842399.1	1.355	2.0036	0.67628
XP_021995117.1	XP_022001254.1	1.1664	1.7246	0.67633
XP_021993551.1	XP_022035551.1	1.7018	2.5153	0.67658
XP_022006327.1	XP_035838717.1	1.3607	2.0111	0.67659
XP_021993620.1	XP_022002418.1	0.9176	1.3561	0.67665
XP_021981407.1	XP_035835830.1	0.9233	1.3645	0.67666

XP_021972810.1	XP_022013239.1	1.2065	1.7829	0.67671
XP_021989733.1	XP_022008275.1	1.0923	1.6141	0.67672
XP_021995117.1	XP_022002416.1	0.8429	1.2446	0.67725
XP_022004979.1	XP_022006668.1	1.0681	1.5767	0.67743
XP_022002418.1	XP_022014870.1	1.0274	1.5166	0.67744
XP_021988229.1	XP_022002418.1	0.8216	1.2126	0.67755
XP_021988953.1	XP_022013560.1	1.0805	1.5939	0.6779
XP_022010291.1	XP_022010295.2	0.6276	0.9258	0.6779
XP_021995117.1	XP_022002417.1	0.8334	1.2292	0.678
XP_021981395.1	XP_022002421.1	1.042	1.5368	0.67803
XP_021973316.1	XP_022008276.2	1.8276	2.6954	0.67804
XP_021981626.1	XP_021993052.1	1.2195	1.7984	0.6781
XP_021972810.1	XP_021993620.1	1.0973	1.6177	0.67831
XP_021978842.1	XP_022002420.1	1.0818	1.5948	0.67833
XP_022021912.1	XP_035838717.1	1.1394	1.6797	0.67834
XP_022002416.1	XP_035835830.1	0.4917	0.7248	0.67839
XP_021993622.1	XP_022013182.1	1.5049	2.2179	0.67852
XP_021987458.1	XP_021988229.1	1.0433	1.5376	0.67852
XP_021984981.2	XP_022021911.1	1.1973	1.7642	0.67866
XP_021986500.1	XP_022013239.1	1.1652	1.7168	0.6787
XP_021995117.1	XP_022014870.1	1.1185	1.6479	0.67874
XP_022008276.2	XP_022010919.1	1.1546	1.7009	0.67882
XP_021987457.1	XP_022008276.2	1.286	1.8943	0.67888
XP_021989737.1	XP_022029740.1	1.074	1.5819	0.67893
XP_021993222.1	XP_022006927.1	1.5356	2.2614	0.67905
XP_021988229.1	XP_022029739.1	0.833	1.2265	0.67917
XP_022001254.1	XP_022029740.1	1.014	1.4925	0.6794
XP_021989733.1	XP_022006668.1	1.2411	1.8265	0.6795
XP_021993221.1	XP_022039085.1	1.4474	2.1293	0.67975
XP_022026963.1	XP_022039074.2	1.2439	1.8294	0.67995
XP_022029740.1	XP_035838717.1	1.0633	1.5636	0.68003
XP_021989737.1	XP_022014870.1	0.8981	1.3205	0.68012
XP_021989734.1	XP_021995116.1	1.2294	1.807	0.68035
XP_022002418.1	XP_022022487.2	0.924	1.3578	0.68051
XP_022006927.1	XP_022039074.2	1.1664	1.7137	0.68063
XP_021993622.1	XP_021995117.1	1.1262	1.6546	0.68065
XP_022002417.1	XP_022039085.1	0.7242	1.0639	0.6807
XP_021981388.1	XP_021984766.1	0.9063	1.3308	0.68102
XP_022001254.1	XP_022002419.1	1.1019	1.6173	0.68132
XP_022014870.1	XP_022022835.1	1.4743	2.1634	0.68147
XP_022010296.1	XP_022013560.1	0.35	0.5135	0.6816
XP_022004455.1	XP_022013558.1	0.6147	0.9015	0.68186
XP_021989736.1	XP_022035551.1	0.9471	1.3888	0.68196
XP_022010919.1	XP_035835830.1	1.0185	1.4931	0.68214
XP_021987458.1	XP_022010294.1	1.0293	1.5088	0.6822
XP_021981626.1	XP_022013558.1	1.0512	1.5406	0.68233
XP_021993622.1	XP_022002415.1	1.107	1.6222	0.68241
XP_021993052.1	XP_022000196.1	1.1591	1.6984	0.68247
XP_021993052.1	XP_022002421.1	0.8731	1.2789	0.6827
XP_021988229.1	XP_021988953.1	1.3549	1.9843	0.68281
XP_022002418.1	XP_022013558.1	0.5149	0.754	0.68289
XP_021989733.1	XP_022010295.2	1.1759	1.7216	0.68303
XP_022029739.1	XP_022034230.1	1.1422	1.6722	0.68305
XP_022002420.1	XP_022034230.1	1.0065	1.4735	0.68307
XP_021968895.1	XP_021973011.1	1.0592	1.5503	0.68322
XP_021972810.1	XP_022022835.1	1.1827	1.731	0.68325
XP_021987457.1	XP_022002416.1	1.1137	1.63	0.68325
XP_021987457.1	XP_022002419.1	1.048	1.5336	0.68336
XP_022002421.1	XP_022022487.2	0.9571	1.4005	0.6834
XP_021973316.1	XP_022010296.1	1.0947	1.601	0.68376
XP_021993052.1	XP_035833070.1	1.2999	1.901	0.6838
XP_021968895.1	XP_022001254.1	1.0622	1.5532	0.68388
XP_035833070.1	XP_035842399.1	1.5584	2.2785	0.68396
XP_022000224.2	XP_022039074.2	0.8823	1.2896	0.68417
XP_022006668.1	XP_022010294.1	0.9885	1.4448	0.68418
XP_021968895.1	XP_022013560.1	0.9476	1.385	0.68419
XP_021989735.1	XP_022026963.1	1.596	2.3318	0.68445
XP_021989733.1	XP_021995116.1	1.2283	1.7942	0.68459
XP_021968895.1	XP_022010919.1	1.1783	1.7211	0.68462
XP_022001254.1	XP_022002415.1	0.9846	1.4381	0.68465
XP_022010616.1	XP_022039085.1	0.9372	1.3687	0.68474
XP_021984766.1	XP_021989734.1	0.9937	1.4503	0.68517
XP_022009213.1	XP_022010292.1	0.98	1.4301	0.68527
XP_021976022.1	XP_022002416.1	1.1995	1.7502	0.68535
XP_021984766.1	XP_022002415.1	1.0759	1.5696	0.68546
XP_022009213.1	XP_022033162.1	1.2499	1.8226	0.68578
XP_021976022.1	XP_022002419.1	1.1632	1.6957	0.68597
XP_021993052.1	XP_022004979.1	1.1096	1.6167	0.68634
XP_021984766.1	XP_022002418.1	1.1214	1.6336	0.68646
XP_021981626.1	XP_021988953.1	1.1204	1.6321	0.68648
XP_022009213.1	XP_022026963.1	1.3492	1.9649	0.68665
XP_021973011.1	XP_022005608.1	0.9447	1.3751	0.687
XP_022000196.1	XP_022006668.1	1.4121	2.0553	0.68705
XP_021976022.1	XP_022010616.1	1.5743	2.2913	0.68708
XP_022002417.1	XP_022021912.1	1.1132	1.6201	0.68712
XP_022001254.1	XP_022013560.1	1.1447	1.6658	0.68718
XP_022001254.1	XP_022009213.1	1.3682	1.9908	0.68726
XP_021981388.1	XP_022002421.1	1.0672	1.5526	0.68736

XP_021987457.1	XP_022035551.1	1.3072	1.9016	0.68742
XP_022002415.1	XP_022002420.1	0.1476	0.2147	0.68747
XP_022010292.1	XP_022013182.1	1.3033	1.8953	0.68765
XP_022021911.1	XP_022029739.1	1.0577	1.5376	0.68789
XP_022000196.1	XP_022006327.1	1.1436	1.6623	0.68796
XP_022002420.1	XP_022010296.1	0.5735	0.8336	0.68798
XP_022010295.2	XP_022010296.1	0.1149	0.167	0.68802
XP_022002415.1	XP_022022487.2	0.9162	1.3316	0.68804
XP_021993620.1	XP_021993621.2	0.1758	0.2555	0.68806
XP_021984766.1	XP_022022487.2	1.337	1.9431	0.68808
XP_022002416.1	XP_022006327.1	1.1587	1.6839	0.6881
XP_022002418.1	XP_022010919.1	0.9756	1.4178	0.68811
XP_021989737.1	XP_022018622.1	1.25	1.8163	0.68821
XP_021987458.1	XP_022002416.1	1.1303	1.6419	0.68841
XP_021989734.1	XP_022010295.2	1.1046	1.6045	0.68844
XP_021968895.1	XP_021988229.1	0.8224	1.1941	0.68872
XP_021973011.1	XP_021978807.1	1.0907	1.5836	0.68875
XP_021987458.1	XP_022002419.1	1.0636	1.5441	0.68882
XP_022002421.1	XP_022004980.1	1.2643	1.8349	0.68903
XP_022002415.1	XP_022021911.1	1.0875	1.5773	0.68947
XP_022002415.1	XP_022021913.1	1.0875	1.5773	0.68947
XP_022002415.1	XP_022021914.1	1.0875	1.5773	0.68947
XP_022006926.1	XP_022022835.1	1.3244	1.9197	0.6899
XP_021984766.1	XP_022013239.1	1.5442	2.2374	0.69018
XP_021981407.1	XP_021993551.1	1.4759	2.1381	0.69029
XP_022010294.1	XP_035838717.1	1.0218	1.4793	0.69073
XP_021968895.1	XP_021978842.1	1.3535	1.9584	0.69113
XP_021993052.1	XP_022026964.1	1.3888	2.009	0.69129
XP_021993052.1	XP_022004455.1	1.0603	1.5336	0.69138
XP_021987457.1	XP_022022487.2	1.592	2.3026	0.69139
XP_021986500.1	XP_022010296.1	0.8532	1.2339	0.69147
XP_022002415.1	XP_022013558.1	0.4972	0.7189	0.69161
XP_021989733.1	XP_022001254.1	1.1499	1.6626	0.69163
XP_022010919.1	XP_022015084.1	1.3954	2.0175	0.69165
XP_022010291.1	XP_022039074.2	0.7614	1.1001	0.69212
XP_021987457.1	XP_022039085.1	1.1545	1.668	0.69215
XP_021981407.1	XP_022008275.1	1.0251	1.481	0.69217
XP_022006926.1	XP_022039074.2	0.9999	1.4444	0.69226
XP_021986500.1	XP_021989736.1	0.9183	1.3263	0.69238
XP_021968895.1	XP_022021911.1	1.3933	2.012	0.6925
XP_021968895.1	XP_022021913.1	1.3933	2.012	0.6925
XP_021968895.1	XP_022021914.1	1.3933	2.012	0.6925
XP_022029622.1	XP_022035384.1	1.547	2.2307	0.6935
XP_021973854.1	XP_021993620.1	2.2025	3.1756	0.69357
XP_021978842.1	XP_022034230.1	1.3743	1.9807	0.69385
XP_021989736.1	XP_022006327.1	1.3545	1.952	0.6939
XP_021995117.1	XP_022004980.1	1.0876	1.5672	0.69398
XP_021973854.1	XP_021978842.1	1.4183	2.0425	0.69439
XP_022000196.1	XP_022021913.1	1.3708	1.9737	0.69453
XP_021989736.1	XP_021993052.1	1.132	1.6279	0.69537
XP_022002415.1	XP_022021912.1	1.0841	1.5586	0.69556
XP_022000196.1	XP_022021911.1	1.2472	1.7921	0.69594
XP_021999258.1	XP_022026963.1	1.5895	2.2834	0.69611
XP_022014870.1	XP_022029739.1	1.0127	1.454	0.69649
XP_022004980.1	XP_022013182.1	1.211	1.7373	0.69706
XP_021987458.1	XP_022013182.1	1.143	1.6397	0.69708
XP_021978842.1	XP_021993052.1	1.3125	1.8826	0.69717
XP_021984766.1	XP_022001254.1	1.2713	1.8232	0.69729
XP_022000224.2	XP_022004980.1	1.1047	1.5832	0.69776
XP_022002417.1	XP_022010295.2	0.5539	0.7938	0.69778
XP_022010291.1	XP_035835830.1	0.6005	0.8605	0.69785
XP_021985239.1	XP_022010292.1	0.8349	1.1962	0.69796
XP_022013560.1	XP_022029740.1	0.6329	0.9067	0.69803
XP_021986500.1	XP_021989735.1	0.9798	1.4033	0.69821
XP_022006926.1	XP_022010291.1	0.8751	1.2532	0.69829
XP_021984766.1	XP_021993551.1	1.6337	2.3391	0.69843
XP_022002417.1	XP_022006327.1	1.2506	1.7904	0.6985
XP_021987458.1	XP_022039085.1	1.1582	1.6578	0.69864
XP_021981395.1	XP_021999258.1	1.3216	1.8916	0.69867
XP_021984766.1	XP_022013560.1	0.89	1.2737	0.69875
XP_021988229.1	XP_022013560.1	0.861	1.2316	0.69909
XP_021993220.1	XP_022014870.1	1.4603	2.0888	0.69911
XP_021981395.1	XP_022029738.1	1.196	1.7106	0.69917
XP_022002418.1	XP_022021911.1	1.0822	1.5478	0.69919
XP_022002418.1	XP_022021913.1	1.0822	1.5478	0.69919
XP_022002418.1	XP_022021914.1	1.0822	1.5478	0.69919
XP_021993222.1	XP_021993620.1	0.8631	1.2339	0.69949
XP_021981388.1	XP_022010291.1	0.9635	1.3764	0.70001
XP_022015084.1	XP_022018622.1	1.01	1.4428	0.70003
XP_021999258.1	XP_022010296.1	1.0325	1.4747	0.70014
XP_022006927.1	XP_022029740.1	0.9597	1.3704	0.70031
XP_021995116.1	XP_022018622.1	1.5483	2.2102	0.70052
XP_021978842.1	XP_022002415.1	1.0856	1.5491	0.70079
XP_021984981.2	XP_035833070.1	1.4779	2.1082	0.70102
XP_022004979.1	XP_022035551.1	1.4217	2.0276	0.70117
XP_022004979.1	XP_022035552.1	1.4217	2.0276	0.70117
XP_022006927.1	XP_022008275.1	1.1748	1.6747	0.7015
XP_021973854.1	XP_021989733.1	1.2812	1.8262	0.70157

XP_022022835.1	XP_022035552.1	1.3818	1.9694	0.70164
XP_022021912.1	XP_022022835.1	1.4308	2.0387	0.70182
XP_021985239.1	XP_035842399.1	1.2484	1.7787	0.70186
XP_022010291.1	XP_022013182.1	1.3515	1.9254	0.70193
XP_021978842.1	XP_022004455.1	1.1237	1.6003	0.70218
XP_021993620.1	XP_022005608.1	1.3103	1.8659	0.70223
XP_021984981.2	XP_022021913.1	1.2061	1.7172	0.70236
XP_021984981.2	XP_022021914.1	1.2061	1.7172	0.70236
XP_022010295.2	XP_022029740.1	0.72	1.0247	0.70264
XP_021995116.1	XP_022029622.1	1.5675	2.2303	0.70282
XP_022002416.1	XP_022010295.2	0.5446	0.7748	0.70289
XP_022004455.1	XP_022010919.1	1.0314	1.4668	0.70316
XP_021968895.1	XP_022013558.1	0.8859	1.2595	0.70337
XP_022022835.1	XP_022039085.1	1.2672	1.8002	0.70392
XP_021987457.1	XP_022006327.1	1.4316	2.0335	0.70401
XP_021993052.1	XP_021995117.1	0.9529	1.3535	0.70403
XP_022002418.1	XP_022021912.1	1.0789	1.5315	0.70447
XP_021973854.1	XP_022010919.1	1.3346	1.8938	0.70472
XP_021993551.1	XP_035838717.1	1.4144	2.007	0.70473
XP_021993222.1	XP_035842399.1	1.8655	2.647	0.70476
XP_022009213.1	XP_022010919.1	1.046	1.4839	0.7049
XP_021968895.1	XP_022033162.1	0.8961	1.2712	0.70492
XP_022026964.1	XP_022029738.1	1.0815	1.5339	0.70507
XP_021969020.1	XP_022004979.1	1.6054	2.2757	0.70545
XP_021981626.1	XP_021993283.1	1.3049	1.8496	0.7055
XP_022022835.1	XP_022029622.1	1.3258	1.879	0.70559
XP_022004455.1	XP_022010616.1	0.9545	1.3525	0.70573
XP_021973316.1	XP_022039085.1	1.3577	1.9237	0.70578
XP_021978807.1	XP_021992525.1	1.1959	1.6944	0.7058
XP_021993620.1	XP_022039074.2	1.1224	1.5898	0.706
XP_021988229.1	XP_022026963.1	1.2103	1.7141	0.70608
XP_022006327.1	XP_022009213.1	1.3976	1.9793	0.70611
XP_021993620.1	XP_035835830.1	1.2376	1.7527	0.70611
XP_022010295.2	XP_022022487.2	0.8507	1.2046	0.70621
XP_021992525.1	XP_022013560.1	1.273	1.8025	0.70624
XP_021993283.1	XP_022021911.1	1.559	2.2064	0.70658
XP_022001254.1	XP_022006327.1	1.2586	1.7806	0.70684
XP_022009213.1	XP_022029738.1	1.2236	1.7309	0.70692
XP_021993052.1	XP_022006927.1	1.0074	1.4249	0.707
XP_021973854.1	XP_035835830.1	1.2184	1.7232	0.70706
XP_021986500.1	XP_022010291.1	1.1098	1.5695	0.7071
XP_021985239.1	XP_022010296.1	0.8771	1.2404	0.70711
XP_021995117.1	XP_022002415.1	0.8221	1.1619	0.70755
XP_021984766.1	XP_022010294.1	0.9901	1.3993	0.70757
XP_022002421.1	XP_022021912.1	1.1284	1.5945	0.70768
XP_021993220.1	XP_022010295.2	0.9473	1.3384	0.70779
XP_022002420.1	XP_022029740.1	0.3986	0.5631	0.70787
XP_022010295.2	XP_035838717.1	0.949	1.3406	0.70789
XP_022002416.1	XP_022021911.1	1.111	1.5686	0.70827
XP_022002416.1	XP_022021913.1	1.111	1.5686	0.70827
XP_022002416.1	XP_022021914.1	1.111	1.5686	0.70827
XP_021993222.1	XP_022021912.1	1.6938	2.3911	0.70838
XP_021972810.1	XP_022021911.1	1.2608	1.7795	0.70851
XP_021972810.1	XP_022021913.1	1.2608	1.7795	0.70851
XP_021972810.1	XP_022021914.1	1.2608	1.7795	0.70851
XP_022002421.1	XP_022035384.1	1.4021	1.9789	0.70852
XP_022021913.1	XP_022029739.1	1.0704	1.5105	0.70864
XP_022021914.1	XP_022029739.1	1.0704	1.5105	0.70864
XP_022013183.1	XP_022039085.1	1.5996	2.2564	0.70892
XP_021972810.1	XP_021985239.1	0.8939	1.2608	0.70899
XP_021987457.1	XP_022010294.1	1.0222	1.4414	0.70917
XP_022010292.1	XP_022026964.1	0.9803	1.3822	0.70923
XP_021972810.1	XP_022029738.1	0.6308	0.8892	0.7094
XP_021992525.1	XP_022018622.1	1.4219	2.0042	0.70946
XP_022013183.1	XP_022029622.1	1.3143	1.8522	0.70959
XP_021989735.1	XP_035842399.1	1.5018	2.1164	0.7096
XP_021988953.1	XP_022000224.2	0.9598	1.3525	0.70965
XP_021993551.1	XP_022035384.1	1.6954	2.3879	0.71
XP_022000224.2	XP_022008276.2	1.2321	1.7352	0.71006
XP_021973854.1	XP_022002419.1	1.1254	1.5846	0.71021
XP_021989737.1	XP_022013239.1	1.4724	2.0721	0.71058
XP_021969020.1	XP_021988229.1	1.7835	2.5088	0.7109
XP_021993221.1	XP_022013558.1	1.2026	1.6915	0.71097
XP_021995116.1	XP_035833070.1	1.2969	1.824	0.71102
XP_021989734.1	XP_022010291.1	1.036	1.4566	0.71125
XP_021973316.1	XP_022002419.1	1.0266	1.4431	0.71139
XP_021978842.1	XP_021986500.1	1.1641	1.6363	0.71142
XP_021973011.1	XP_021992525.1	1.276	1.7932	0.71158
XP_021992525.1	XP_022010291.1	1.4328	2.0132	0.7117
XP_021981626.1	XP_022002417.1	1.0265	1.4418	0.71196
XP_021973854.1	XP_021993551.1	1.3214	1.8555	0.71215
XP_022004455.1	XP_022029738.1	0.5043	0.708	0.71229
XP_021987457.1	XP_022000196.1	1.4776	2.0727	0.71289
XP_022004980.1	XP_022013183.1	1.175	1.6479	0.71303
XP_022000224.2	XP_022001254.1	1.3904	1.9499	0.71306
XP_022002418.1	XP_022002421.1	0.2018	0.283	0.71307
XP_022018622.1	XP_022039085.1	1.1926	1.6716	0.71345
XP_021989735.1	XP_022006668.1	1.2316	1.7259	0.7136

XP_021987458.1	XP_022010295.2	1.3098	1.8351	0.71375
XP_021984766.1	XP_021989737.1	1.0353	1.4505	0.71375
XP_022004980.1	XP_022006327.1	1.5756	2.2073	0.71381
XP_022009213.1	XP_022010294.1	1.0526	1.4743	0.71397
XP_021968895.1	XP_022010294.1	0.9346	1.3089	0.71403
XP_021981395.1	XP_022026964.1	1.1966	1.6757	0.71409
XP_021988953.1	XP_022002415.1	0.9831	1.3767	0.7141
XP_021993620.1	XP_022000196.1	1.2894	1.8056	0.71411
XP_022013183.1	XP_022013558.1	1.1774	1.6479	0.71449
XP_021973011.1	XP_021989736.1	1.1159	1.5615	0.71463
XP_021973316.1	XP_022006926.1	1.2735	1.7816	0.71481
XP_021973854.1	XP_022010616.1	1.3322	1.8637	0.71481
XP_021984981.2	XP_022013239.1	1.339	1.8732	0.71482
XP_022002420.1	XP_022010919.1	0.9413	1.3168	0.71484
XP_021984981.2	XP_035835830.1	1.0093	1.4117	0.71495
XP_021995117.1	XP_022009213.1	1.0203	1.427	0.715
XP_021968895.1	XP_021989737.1	1.161	1.6236	0.71508
XP_021984766.1	XP_022021913.1	1.5692	2.1944	0.71509
XP_021984766.1	XP_022021914.1	1.5692	2.1944	0.71509
XP_021992525.1	XP_022013558.1	1.2171	1.7015	0.71531
XP_021993221.1	XP_022018622.1	1.4042	1.963	0.71533
XP_022013183.1	XP_022039074.2	1.3693	1.9131	0.71575
XP_021989734.1	XP_022000196.1	1.0785	1.5068	0.71576
XP_021981388.1	XP_022035384.1	1.2422	1.7355	0.71576
XP_021986500.1	XP_021995116.1	0.8696	1.2144	0.71607
XP_021978807.1	XP_022013239.1	1.2717	1.7758	0.71613
XP_022002419.1	XP_022029739.1	0.3957	0.5525	0.7162
XP_021981407.1	XP_022005608.1	1.1029	1.5399	0.71622
XP_021973011.1	XP_022029740.1	1.0018	1.398	0.7166
XP_022002419.1	XP_022006327.1	1.3223	1.8448	0.71677
XP_021973316.1	XP_021993221.1	1.4737	2.0548	0.7172
XP_022001254.1	XP_022034230.1	1.3998	1.951	0.71748
XP_022004455.1	XP_022029739.1	0.5128	0.7147	0.7175
XP_021978807.1	XP_022014870.1	1.0311	1.4362	0.71794
XP_021985239.1	XP_022026963.1	1.1546	1.6076	0.71821
XP_021993052.1	XP_022002417.1	0.7808	1.0871	0.71824
XP_021993283.1	XP_022004979.1	1.0946	1.523	0.71871
XP_022000196.1	XP_022021914.1	1.2695	1.7656	0.71902
XP_021972810.1	XP_022010291.1	0.6685	0.9291	0.71951
XP_021969020.1	XP_022035384.1	1.3995	1.9444	0.71976
XP_022013182.1	XP_022013558.1	1.2345	1.7148	0.71991
XP_022004979.1	XP_022005608.1	1.3294	1.8464	0.72
XP_021987458.1	XP_022013183.1	1.1555	1.6042	0.7203
XP_021969020.1	XP_022006927.1	1.3111	1.8201	0.72035
XP_021995116.1	XP_022029739.1	1.235	1.7143	0.72041
XP_021981407.1	XP_022006926.1	1.3905	1.9298	0.72054
XP_022006668.1	XP_022013239.1	1.3442	1.8653	0.72063
XP_021981626.1	XP_022002416.1	1.0217	1.4176	0.72073
XP_021995116.1	XP_022029740.1	1.2034	1.6696	0.72077
XP_022013239.1	XP_022022835.1	1.1748	1.6299	0.72078
XP_021968895.1	XP_021973316.1	1.3185	1.8291	0.72085
XP_021989734.1	XP_022006668.1	1.2592	1.7464	0.72103
XP_021981388.1	XP_021989737.1	0.4289	0.5946	0.72133
XP_021989736.1	XP_022010291.1	1.0336	1.4328	0.72138
XP_021972810.1	XP_022006327.1	1.285	1.7804	0.72175
XP_021993221.1	XP_021993551.1	1.3876	1.9225	0.72177
XP_022002419.1	XP_022026964.1	0.9609	1.3311	0.72188
XP_022004979.1	XP_022018622.1	1.3797	1.911	0.72198
XP_021987457.1	XP_021989734.1	1.1284	1.5628	0.72204
XP_021973854.1	XP_022002420.1	1.1499	1.5921	0.72225
XP_021995116.1	XP_022006927.1	1.3089	1.8122	0.72227
XP_022022835.1	XP_022035551.1	1.4045	1.9436	0.72263
XP_022004980.1	XP_022013560.1	1.0571	1.4628	0.72266
XP_021973316.1	XP_021992525.1	1.3486	1.8661	0.72268
XP_022010294.1	XP_022013239.1	0.9824	1.3592	0.72278
XP_022010616.1	XP_022018622.1	1.0528	1.4563	0.72293
XP_021984981.2	XP_021993551.1	1.3554	1.8742	0.72319
XP_021968895.1	XP_022004455.1	0.8321	1.1504	0.72331
XP_022002419.1	XP_022005608.1	0.9336	1.2906	0.72338
XP_021993221.1	XP_021995117.1	1.0804	1.4933	0.7235
XP_021981388.1	XP_021981626.1	1.024	1.4153	0.72352
XP_021986500.1	XP_035842399.1	1.1368	1.5707	0.72375
XP_021981626.1	XP_022010296.1	1.1536	1.5937	0.72385
XP_022000196.1	XP_022029740.1	1.2066	1.6664	0.72408
XP_021993220.1	XP_022029739.1	1.2273	1.6948	0.72416
XP_022009213.1	XP_022029740.1	1.113	1.5366	0.72433
XP_022002421.1	XP_022010919.1	0.9995	1.3798	0.72438
XP_021981388.1	XP_021984981.2	0.7937	1.0956	0.72444
XP_021993222.1	XP_022002421.1	1.0327	1.4255	0.72445
XP_021981626.1	XP_021989736.1	1.1911	1.6438	0.7246
XP_021976022.1	XP_035835830.1	1.4844	2.0485	0.72463
XP_021992525.1	XP_022039085.1	1.5532	2.1433	0.72468
XP_021972810.1	XP_021989736.1	1.2262	1.692	0.7247
XP_021987458.1	XP_022022487.2	1.655	2.2834	0.7248
XP_021992525.1	XP_022000196.1	1.4403	1.9862	0.72515
XP_022006927.1	XP_022010294.1	0.9014	1.2426	0.72541
XP_021993622.1	XP_022002418.1	1.1607	1.599	0.72589
XP_022029740.1	XP_022039074.2	0.7214	0.9938	0.7259

XP_021989736.1	XP_022026964.1	1.0274	1.4153	0.72592
XP_022021912.1	XP_022022487.2	1.3983	1.9259	0.72605
XP_021972810.1	XP_021999258.1	1.2781	1.7603	0.72607
XP_022004979.1	XP_022013239.1	1.288	1.7738	0.72612
XP_021987458.1	XP_021989734.1	1.1526	1.5872	0.72618
XP_022008276.2	XP_022035384.1	1.5849	2.1824	0.72622
XP_022002419.1	XP_022010296.1	0.6407	0.8822	0.72625
XP_021969020.1	XP_021993551.1	1.6499	2.2716	0.72632
XP_022002416.1	XP_022013560.1	0.6007	0.827	0.72636
XP_022026963.1	XP_022026964.1	0.4456	0.6134	0.72644
XP_022000196.1	XP_022014870.1	1.3929	1.9173	0.72649
XP_022039074.2	XP_035842399.1	1.505	2.0713	0.7266
XP_022005608.1	XP_022021912.1	1.2034	1.6557	0.72682
XP_021989737.1	XP_022001254.1	1.2013	1.6524	0.727
XP_021993551.1	XP_022013560.1	1.3293	1.8284	0.72703
XP_021993222.1	XP_022008275.1	1.205	1.6558	0.72774
XP_022009213.1	XP_022022487.2	1.2686	1.7431	0.72778
XP_021985239.1	XP_022021911.1	1.3024	1.7893	0.72788
XP_021985239.1	XP_022021913.1	1.3024	1.7893	0.72788
XP_021985239.1	XP_022021914.1	1.3024	1.7893	0.72788
XP_022001254.1	XP_022010294.1	1.1194	1.5376	0.72802
XP_022002419.1	XP_022022835.1	1.0838	1.4879	0.72841
XP_022010296.1	XP_022010919.1	0.9876	1.3555	0.72859
XP_021993622.1	XP_022004979.1	1.2071	1.6564	0.72875
XP_021993221.1	XP_022010295.2	1.2134	1.6649	0.72881
XP_021989735.1	XP_022010291.1	1.0547	1.4467	0.72904
XP_022010296.1	XP_035838717.1	0.9632	1.3206	0.72937
XP_021995116.1	XP_022010919.1	1.666	2.2834	0.72961
XP_021984766.1	XP_022013558.1	1.0122	1.3869	0.72983
XP_021988229.1	XP_022010291.1	0.9355	1.2817	0.72989
XP_022013183.1	XP_022022835.1	1.3208	1.8095	0.72993
XP_021976022.1	XP_021989734.1	1.1193	1.5333	0.72999
XP_021993620.1	XP_022002416.1	0.9465	1.2964	0.7301
XP_021989734.1	XP_022013239.1	1.4268	1.9542	0.73012
XP_021993220.1	XP_022002420.1	1.0237	1.402	0.73017
XP_021973854.1	XP_021989735.1	1.3492	1.8477	0.73021
XP_021984766.1	XP_022002420.1	1.1214	1.5356	0.73027
XP_021986500.1	XP_021993052.1	0.9984	1.3669	0.73041
XP_021989734.1	XP_022022835.1	1.2518	1.7137	0.73047
XP_021993052.1	XP_021993622.1	1.6323	2.2335	0.73083
XP_022006668.1	XP_022013558.1	0.9165	1.2538	0.73098
XP_021985239.1	XP_021989737.1	1.0715	1.4658	0.731
XP_021993622.1	XP_035833070.1	1.3165	1.8003	0.73127
XP_022010291.1	XP_022013239.1	1.0996	1.5035	0.73136
XP_022005608.1	XP_022009213.1	1.041	1.4231	0.7315
XP_021981626.1	XP_022004979.1	1.1949	1.6333	0.73159
XP_021999258.1	XP_022010292.1	1.0966	1.4989	0.7316
XP_021976022.1	XP_021989735.1	1.219	1.6662	0.7316
XP_022008276.2	XP_022026964.1	1.1972	1.6364	0.73161
XP_022006327.1	XP_022018622.1	1.5226	2.0799	0.73205
XP_022002420.1	XP_022010295.2	0.5574	0.7612	0.73226
XP_022006926.1	XP_022035384.1	1.2023	1.6417	0.73235
XP_021995116.1	XP_021995117.1	1.1161	1.5236	0.73254
XP_021984981.2	XP_021993283.1	1.4208	1.9395	0.73256
XP_022005608.1	XP_035838717.1	1.0224	1.3956	0.73259
XP_022010292.1	XP_022022835.1	1.0097	1.3777	0.73289
XP_022006327.1	XP_022010616.1	1.5938	2.1741	0.73308
XP_021985239.1	XP_021995117.1	0.9185	1.2529	0.7331
XP_021993222.1	XP_022000196.1	1.5598	2.1267	0.73344
XP_021981626.1	XP_022013239.1	1.444	1.9677	0.73385
XP_021972810.1	XP_035833070.1	1.1935	1.6257	0.73415
XP_021986500.1	XP_021988229.1	1.1711	1.5951	0.73419
XP_021993052.1	XP_022010296.1	0.8747	1.1913	0.73424
XP_021989733.1	XP_021993220.1	1.4898	2.0289	0.73429
XP_021978807.1	XP_021989733.1	1.2559	1.7098	0.73453
XP_021973854.1	XP_022013560.1	1.1628	1.5823	0.73488
XP_021988953.1	XP_022014870.1	1.1201	1.5241	0.73493
XP_021981407.1	XP_022013182.1	0.8645	1.1758	0.73524
XP_022013560.1	XP_022022835.1	1.1908	1.6195	0.73529
XP_021973855.1	XP_021989737.1	1.821	2.4765	0.73531
XP_021984981.2	XP_022035551.1	0.8783	1.1941	0.73553
XP_021984766.1	XP_021989733.1	1.0139	1.3783	0.73562
XP_021978842.1	XP_021988953.1	1.2822	1.7428	0.73571
XP_022010292.1	XP_022035384.1	0.9944	1.3514	0.73583
XP_022002421.1	XP_022021913.1	1.1116	1.5105	0.73592
XP_022002421.1	XP_022021914.1	1.1116	1.5105	0.73592
XP_021986500.1	XP_021992525.1	1.0938	1.486	0.73607
XP_022000196.1	XP_022039074.2	1.222	1.66	0.73614
XP_021993052.1	XP_022010616.1	1.1369	1.5443	0.73619
XP_022010291.1	XP_022014870.1	1.0763	1.4616	0.73638
XP_021976022.1	XP_022002418.1	1.1838	1.6075	0.73642
XP_021984981.2	XP_022001254.1	1.354	1.8386	0.73643
XP_022013182.1	XP_022022835.1	1.3496	1.8323	0.73656
XP_021987458.1	XP_021989735.1	1.1882	1.6129	0.73669
XP_021988229.1	XP_021993622.1	1.4844	2.0132	0.73733
XP_021993052.1	XP_022022835.1	1.3319	1.806	0.73749
XP_022000196.1	XP_022002416.1	1.0174	1.3795	0.73751
XP_022006327.1	XP_022013182.1	1.1983	1.6243	0.73773

XP_021988953.1	XP_022004980.1	1.264	1.7125	0.7381
XP_021993220.1	XP_022034230.1	1.2021	1.6285	0.73816
XP_022010291.1	XP_022029740.1	0.5393	0.7304	0.73836
XP_022010296.1	XP_022013182.1	1.307	1.7687	0.73896
XP_021993620.1	XP_035838717.1	1.2287	1.6626	0.73902
XP_021978842.1	XP_022005608.1	1.0637	1.4389	0.73925
XP_022004979.1	XP_022021911.1	1.2691	1.7167	0.73927
XP_022004979.1	XP_022021913.1	1.2691	1.7167	0.73927
XP_022004979.1	XP_022021914.1	1.2691	1.7167	0.73927
XP_021973316.1	XP_022013183.1	1.4267	1.9293	0.73949
XP_021988953.1	XP_022010616.1	1.2644	1.7098	0.7395
XP_021986500.1	XP_022014870.1	0.8833	1.1942	0.73966
XP_022001254.1	XP_022010292.1	1.1189	1.5127	0.73967
XP_021973854.1	XP_022000196.1	1.4917	2.0166	0.73971
XP_022002420.1	XP_022026963.1	1.0461	1.4138	0.73992
XP_022002417.1	XP_022018622.1	0.9215	1.2451	0.7401
XP_022013558.1	XP_022013560.1	0.3028	0.4091	0.74016
XP_021992525.1	XP_022029738.1	1.3894	1.8771	0.74018
XP_022008276.2	XP_022013239.1	1.5237	2.0581	0.74034
XP_022002419.1	XP_022039085.1	0.6462	0.8723	0.7408
XP_022013558.1	XP_022022487.2	0.8315	1.1223	0.74089
XP_021988229.1	XP_022010101.1	1.1659	1.5733	0.74105
XP_021999258.1	XP_022008275.1	1.5962	2.1538	0.74111
XP_021985239.1	XP_022010295.2	0.8546	1.1531	0.74113
XP_021981395.1	XP_035835830.1	1.0159	1.3705	0.74126
XP_021993622.1	XP_022010294.1	1.345	1.8141	0.74141
XP_021973854.1	XP_021993052.1	1.312	1.7693	0.74154
XP_021993052.1	XP_022002416.1	0.7682	1.0359	0.74158
XP_022006327.1	XP_022035384.1	1.4713	1.9837	0.74169
XP_022005608.1	XP_022018622.1	1.0384	1.3999	0.74177
XP_022004455.1	XP_022010294.1	0.5814	0.7837	0.74187
XP_021993620.1	XP_022010919.1	1.3381	1.8034	0.74199
XP_021993052.1	XP_022005608.1	1.1393	1.535	0.74221
XP_021978807.1	XP_021981626.1	1.1901	1.6031	0.74237
XP_021981626.1	XP_022002415.1	0.9683	1.3042	0.74245
XP_022001254.1	XP_022010295.2	1.1155	1.5023	0.74253
XP_021993052.1	XP_022018622.1	0.6857	0.9234	0.74258
XP_021986500.1	XP_022000196.1	1.2156	1.6362	0.74294
XP_021973854.1	XP_022013182.1	1.4721	1.9813	0.743
XP_022001254.1	XP_022002421.1	1.1853	1.5952	0.74304
XP_021985239.1	XP_021993622.1	1.6371	2.2029	0.74316
XP_021988953.1	XP_022002417.1	1.1182	1.504	0.74348
XP_021973854.1	XP_022013558.1	1.158	1.5573	0.74359
XP_021978842.1	XP_021989737.1	0.9585	1.289	0.7436
XP_021993052.1	XP_022039085.1	1.0485	1.4099	0.74367
XP_021978842.1	XP_021981395.1	1.112	1.4947	0.74396
XP_021985239.1	XP_021993620.1	1.2361	1.6611	0.74415
XP_021989736.1	XP_021995116.1	1.3712	1.842	0.74441
XP_021993221.1	XP_022010296.1	1.289	1.7312	0.74457
XP_022002416.1	XP_022021912.1	1.1045	1.4829	0.74482
XP_021981388.1	XP_022022835.1	1.3578	1.8225	0.74502
XP_022008276.2	XP_035833070.1	1.2241	1.6426	0.74522
XP_021992525.1	XP_021993222.1	1.4881	1.9965	0.74535
XP_022002418.1	XP_022018622.1	0.8482	1.1377	0.74554
XP_021981388.1	XP_022029738.1	1.1429	1.5329	0.74558
XP_021989736.1	XP_022005608.1	0.9785	1.3124	0.74558
XP_022006327.1	XP_022039085.1	1.4731	1.9749	0.74591
XP_021993221.1	XP_022034230.1	1.5085	2.0213	0.7463
XP_022010294.1	XP_022039074.2	0.8579	1.1492	0.74652
XP_022004980.1	XP_022009213.1	1.1328	1.5174	0.74654
XP_021978807.1	XP_021993621.2	1.1675	1.5637	0.74663
XP_021987457.1	XP_021992525.1	1.2933	1.7321	0.74667
XP_021978842.1	XP_022035384.1	1.5087	2.0204	0.74673
XP_021988953.1	XP_022013239.1	1.3904	1.8609	0.74717
XP_022006927.1	XP_022021913.1	1.3738	1.8383	0.74732
XP_022006927.1	XP_022021914.1	1.3738	1.8383	0.74732
XP_022006927.1	XP_022034230.1	0.7629	1.0207	0.74743
XP_021976022.1	XP_022002415.1	1.1835	1.5834	0.74744
XP_021973011.1	XP_021989735.1	1.1516	1.5406	0.7475
XP_022006327.1	XP_022010919.1	1.1737	1.5697	0.74772
XP_021993283.1	XP_022006668.1	1.9014	2.5412	0.74823
XP_021978842.1	XP_022006927.1	1.3505	1.8046	0.74837
XP_021973011.1	XP_021988229.1	1.1433	1.5277	0.74838
XP_021978842.1	XP_022013560.1	1.175	1.5699	0.74846
XP_021993222.1	XP_022029622.1	1.3476	1.7995	0.74887
XP_021978807.1	XP_022035551.1	1.2108	1.6167	0.74893
XP_021978807.1	XP_022035552.1	1.2108	1.6167	0.74893
XP_021989735.1	XP_022005608.1	1.0165	1.3572	0.74897
XP_021978807.1	XP_022037775.1	1.1305	1.5093	0.74902
XP_021993621.2	XP_022022835.1	1.2207	1.6297	0.74903
XP_022010291.1	XP_022029738.1	0.7417	0.99	0.74919
XP_022014870.1	XP_022037775.1	1.1515	1.5369	0.74924
XP_021989735.1	XP_021995116.1	1.3762	1.8361	0.74952
XP_022004455.1	XP_022005608.1	0.917	1.2231	0.74973
XP_021989734.1	XP_022039085.1	1.2163	1.6222	0.74978
XP_021989736.1	XP_022039074.2	1.1611	1.5474	0.75036
XP_022006327.1	XP_022029738.1	1.2849	1.7123	0.75039
XP_021995116.1	XP_022002420.1	1.2161	1.6201	0.75063

XP_022005608.1	XP_022006926.1	1.1996	1.5975	0.75092
XP_021968895.1	XP_022006668.1	1.3339	1.7763	0.75094
XP_021969020.1	XP_022010294.1	1.0511	1.3994	0.75111
XP_021987457.1	XP_022006926.1	1.5116	2.0124	0.75114
XP_021988953.1	XP_022035384.1	1.3787	1.8353	0.75121
XP_021988229.1	XP_022001254.1	1.346	1.7917	0.75124
XP_022006327.1	XP_022029739.1	1.3229	1.7608	0.75131
XP_022006668.1	XP_022010292.1	0.9628	1.2815	0.75131
XP_021993622.1	XP_022002420.1	1.1971	1.5933	0.75133
XP_022002421.1	XP_022026964.1	0.9811	1.3058	0.75134
XP_021969020.1	XP_021973316.1	1.6595	2.207	0.75193
XP_022002420.1	XP_022004980.1	1.157	1.5378	0.75237
XP_021981388.1	XP_022014870.1	0.9517	1.2648	0.75245
XP_022013182.1	XP_022039074.2	1.3812	1.8349	0.75274
XP_021973316.1	XP_022001254.1	1.1074	1.4709	0.75287
XP_021984981.2	XP_021985239.1	1.026	1.3624	0.75308
XP_022010291.1	XP_022013183.1	1.3111	1.7408	0.75316
XP_021973855.1	XP_022004455.1	1.3676	1.8158	0.75317
XP_021978807.1	XP_022039074.2	1.058	1.4046	0.75324
XP_021972810.1	XP_022009213.1	1.237	1.6419	0.7534
XP_021978807.1	XP_022033162.1	1.1809	1.5673	0.75346
XP_021973855.1	XP_022010616.1	1.3205	1.7525	0.7535
XP_021973011.1	XP_022033162.1	1.0595	1.4061	0.7535
XP_022013239.1	XP_022029738.1	1.1755	1.5596	0.75372
XP_022010101.1	XP_035833070.1	1.0976	1.4562	0.75374
XP_021989733.1	XP_022035552.1	0.901	1.1953	0.75379
XP_021972810.1	XP_022035551.1	1.1993	1.5909	0.75385
XP_021972810.1	XP_022035552.1	1.1993	1.5909	0.75385
XP_021984981.2	XP_022021912.1	1.1709	1.5526	0.75415
XP_022002421.1	XP_022010291.1	0.6375	0.8451	0.75435
XP_021993222.1	XP_022039074.2	1.4271	1.8917	0.7544
XP_021973855.1	XP_021984766.1	1.8391	2.4378	0.75441
XP_022010295.2	XP_022039074.2	0.8179	1.0841	0.75445
XP_021973011.1	XP_021989733.1	1.0458	1.3858	0.75465
XP_021987457.1	XP_021989733.1	1.0428	1.3817	0.75472
XP_022022487.2	XP_035833070.1	1.4265	1.89	0.75476
XP_022021911.1	XP_035838717.1	1.1981	1.5867	0.75509
XP_022029740.1	XP_022039085.1	0.7246	0.9596	0.75511
XP_022002416.1	XP_022039085.1	0.6787	0.8987	0.7552
XP_022002421.1	XP_035835830.1	0.5099	0.6749	0.75552
XP_021993551.1	XP_021995116.1	1.5891	2.1025	0.75581
XP_021981388.1	XP_021999258.1	1.2849	1.6998	0.75591
XP_022010291.1	XP_022013560.1	0.5648	0.747	0.75609
XP_022021913.1	XP_035838717.1	1.204	1.5921	0.75623
XP_022021914.1	XP_035838717.1	1.204	1.5921	0.75623
XP_021984981.2	XP_021993052.1	1.4605	1.9311	0.7563
XP_021985239.1	XP_021988229.1	0.8148	1.0773	0.75634
XP_021973855.1	XP_021989735.1	1.7773	2.3494	0.75649
XP_022010291.1	XP_022029739.1	0.7003	0.9256	0.75659
XP_021984981.2	XP_022035552.1	0.8925	1.1794	0.75674
XP_022004979.1	XP_022039074.2	1.0725	1.4171	0.75683
XP_021989736.1	XP_022010295.2	1.0667	1.4092	0.75695
XP_021993620.1	XP_022021911.1	1.3349	1.7635	0.75696
XP_021993620.1	XP_022021913.1	1.3349	1.7635	0.75696
XP_021993620.1	XP_022021914.1	1.3349	1.7635	0.75696
XP_021981407.1	XP_022022835.1	1.1792	1.5578	0.75696
XP_022002417.1	XP_022026964.1	0.9522	1.2579	0.75698
XP_022022487.2	XP_022029740.1	1.0099	1.3334	0.75739
XP_021973011.1	XP_022014870.1	1.125	1.4851	0.75752
XP_021987458.1	XP_021989737.1	1.082	1.4278	0.75781
XP_021993052.1	XP_022002418.1	0.7706	1.0168	0.75787
XP_021993621.2	XP_022004980.1	1.3248	1.7478	0.75798
XP_021993220.1	XP_022001254.1	1.5346	2.0244	0.75805
XP_022021912.1	XP_022029738.1	1.1465	1.5121	0.75822
XP_021984981.2	XP_022039074.2	1.2545	1.6544	0.75828
XP_021993283.1	XP_022010294.1	1.4644	1.9307	0.75848
XP_021969020.1	XP_022010616.1	1.2425	1.6381	0.7585
XP_022002415.1	XP_022010919.1	0.9982	1.3159	0.75857
XP_022002416.1	XP_022029739.1	0.4446	0.5859	0.75883
XP_021981407.1	XP_021993220.1	1.4081	1.8556	0.75884
XP_021981626.1	XP_022039085.1	1.6818	2.2155	0.75911
XP_021986500.1	XP_022005608.1	0.9607	1.2648	0.75957
XP_021987457.1	XP_022010101.1	1.3675	1.8003	0.7596
XP_022006926.1	XP_022021913.1	1.7216	2.2653	0.75999
XP_022006926.1	XP_022021914.1	1.7216	2.2653	0.75999
XP_021978807.1	XP_022013183.1	1.1826	1.555	0.76051
XP_022010292.1	XP_022010295.2	0.1716	0.2256	0.76064
XP_021987458.1	XP_021989733.1	1.0547	1.3865	0.76069
XP_022002419.1	XP_022013560.1	0.6429	0.8449	0.76092
XP_021968895.1	XP_022010292.1	0.9314	1.224	0.76095
XP_021987457.1	XP_021995116.1	1.6033	2.1069	0.76098
XP_022000196.1	XP_022010294.1	1.1458	1.5055	0.76108
XP_022002421.1	XP_022006926.1	0.885	1.1621	0.76155
XP_022013239.1	XP_022013558.1	0.9381	1.231	0.76206
XP_021989737.1	XP_022010295.2	0.9941	1.3044	0.76211
XP_021973855.1	XP_021984981.2	1.736	2.2778	0.76214
XP_021993221.1	XP_022000224.2	1.5921	2.0888	0.76221
XP_021984981.2	XP_022013182.1	0.9566	1.255	0.76223

XP_021993283.1	XP_022010101.1	1.4549	1.9087	0.76225
XP_021988229.1	XP_022029740.1	0.8634	1.1326	0.76232
XP_021989734.1	XP_022022487.2	1.2955	1.6981	0.76291
XP_021985239.1	XP_022006926.1	1.2146	1.5913	0.76328
XP_022002418.1	XP_022039085.1	0.7004	0.9175	0.76338
XP_021981388.1	XP_022026964.1	1.132	1.4817	0.76399
XP_021993222.1	XP_022022835.1	1.4716	1.9255	0.76427
XP_021989734.1	XP_022008275.1	1.0492	1.3728	0.76428
XP_021993621.2	XP_022037775.1	1.6108	2.1067	0.76461
XP_022000224.2	XP_022002419.1	0.9182	1.2008	0.76466
XP_022002417.1	XP_035842399.1	1.174	1.5348	0.76492
XP_022010919.1	XP_022013560.1	0.9903	1.2946	0.76495
XP_021968895.1	XP_021993222.1	1.2657	1.6543	0.7651
XP_022005608.1	XP_022026964.1	1.2826	1.6749	0.76578
XP_021993222.1	XP_022035384.1	1.7209	2.2471	0.76583
XP_022002415.1	XP_022039085.1	0.6883	0.8978	0.76665
XP_021993620.1	XP_022022487.2	1.5408	2.0096	0.76672
XP_022001254.1	XP_022010919.1	1.428	1.8623	0.76679
XP_021995116.1	XP_022037775.1	1.349	1.7587	0.76704
XP_021985239.1	XP_022010294.1	0.8577	1.1177	0.76738
XP_022010291.1	XP_022013558.1	0.535	0.6966	0.76802
XP_021984766.1	XP_022021911.1	1.5491	2.0159	0.76844
XP_021989733.1	XP_022000196.1	1.2079	1.5714	0.76868
XP_021987457.1	XP_022029622.1	1.4475	1.8828	0.7688
XP_021981626.1	XP_021986500.1	1.1293	1.4689	0.76881
XP_022010296.1	XP_022026964.1	1.0171	1.3227	0.76896
XP_022018622.1	XP_022029739.1	0.9687	1.2594	0.76918
XP_021989733.1	XP_022035551.1	0.8969	1.1658	0.76934
XP_022010101.1	XP_035842399.1	1.6494	2.1439	0.76935
XP_022002417.1	XP_022029739.1	0.4762	0.6186	0.7698
XP_021989735.1	XP_022008275.1	1.0949	1.4221	0.76992
XP_021987457.1	XP_021989737.1	1.0828	1.4058	0.77024
XP_021993220.1	XP_022029738.1	0.9214	1.1959	0.77047
XP_022002417.1	XP_022002421.1	0.2177	0.2825	0.77062
XP_021973316.1	XP_021989736.1	1.2624	1.6381	0.77065
XP_022006668.1	XP_022010296.1	0.9548	1.2389	0.77068
XP_021993620.1	XP_022009213.1	1.2755	1.655	0.77069
XP_021993222.1	XP_022010101.1	1.5439	2.0027	0.77091
XP_021981388.1	XP_021989735.1	0.4062	0.5269	0.77092
XP_021993621.2	XP_022006668.1	1.1823	1.5332	0.77113
XP_021987457.1	XP_022010296.1	1.2386	1.6058	0.77133
XP_022018622.1	XP_022021912.1	1.045	1.354	0.77179
XP_022004455.1	XP_022018622.1	1.142	1.4794	0.77193
XP_021988229.1	XP_021993220.1	1.2958	1.6785	0.772
XP_021987457.1	XP_021989735.1	1.1931	1.5454	0.77203
XP_021987458.1	XP_021989736.1	1.1515	1.4914	0.77209
XP_021986500.1	XP_021989733.1	1.0212	1.3225	0.77217
XP_021981407.1	XP_021984981.2	0.7638	0.9887	0.77253
XP_022006668.1	XP_022010295.2	0.918	1.1881	0.77266
XP_022002418.1	XP_022029739.1	0.4379	0.5667	0.77272
XP_022013560.1	XP_022035384.1	1.2173	1.575	0.77289
XP_022013239.1	XP_035842399.1	1.5413	1.9935	0.77316
XP_021988953.1	XP_022021913.1	1.3578	1.7552	0.77359
XP_021988953.1	XP_022021914.1	1.3578	1.7552	0.77359
XP_022021912.1	XP_035842399.1	1.8329	2.3679	0.77406
XP_021993052.1	XP_021993551.1	1.2398	1.6016	0.7741
XP_021981388.1	XP_022013239.1	1.2485	1.6125	0.77426
XP_021988953.1	XP_022002416.1	1.0447	1.349	0.77443
XP_021978842.1	XP_021993551.1	1.4833	1.9142	0.77489
XP_021989737.1	XP_022005608.1	1.0487	1.3533	0.77492
XP_021973316.1	XP_022010295.2	1.12	1.4452	0.77498
XP_022000224.2	XP_022002416.1	0.8297	1.0706	0.77499
XP_022000224.2	XP_022002417.1	0.8297	1.0706	0.77499
XP_022005608.1	XP_022010101.1	1.263	1.6297	0.77499
XP_022002417.1	XP_022013560.1	0.6085	0.7851	0.77506
XP_021989736.1	XP_022022487.2	1.1224	1.4463	0.77605
XP_021995117.1	XP_022002419.1	0.967	1.2459	0.77615
XP_022000224.2	XP_022035551.1	1.251	1.6117	0.7762
XP_022000224.2	XP_022035552.1	1.251	1.6117	0.7762
XP_022002421.1	XP_022021911.1	1.1043	1.4222	0.77647
XP_021988953.1	XP_035838717.1	1.1485	1.4777	0.77722
XP_021993222.1	XP_021993283.1	1.5426	1.9846	0.77729
XP_021973854.1	XP_021989736.1	1.3295	1.71	0.77749
XP_022004979.1	XP_022035384.1	1.366	1.7555	0.77813
XP_021978807.1	XP_022013182.1	1.237	1.5896	0.77818
XP_021993222.1	XP_022039085.1	1.7582	2.2592	0.77824
XP_021973854.1	XP_022029738.1	1.2885	1.6555	0.77831
XP_022006327.1	XP_022029740.1	1.1898	1.5283	0.77851
XP_021989737.1	XP_021993621.2	1.1391	1.4625	0.77887
XP_021993220.1	XP_021993551.1	1.5819	2.031	0.77888
XP_021989733.1	XP_022039074.2	1.0502	1.3482	0.77896
XP_021981395.1	XP_021988953.1	1.1739	1.5068	0.77907
XP_022000224.2	XP_022002415.1	0.8043	1.0318	0.77951
XP_022002421.1	XP_022029739.1	0.4684	0.6008	0.77963
XP_022013182.1	XP_022029622.1	1.3671	1.7535	0.77964
XP_021972810.1	XP_021988229.1	0.8374	1.074	0.7797
XP_022001254.1	XP_022008275.1	1.2918	1.6566	0.77979
XP_021987458.1	XP_022006926.1	1.5219	1.9512	0.77998

XP_022010296.1	XP_022022835.1	1.1796	1.512	0.78016
XP_022022487.2	XP_022029739.1	1.0545	1.3509	0.78059
XP_021985239.1	XP_022033162.1	1.0205	1.3072	0.78068
XP_021987458.1	XP_021995116.1	1.6301	2.0878	0.78077
XP_022029740.1	XP_022035384.1	1.1637	1.4902	0.7809
XP_021989735.1	XP_022026964.1	1.1288	1.4454	0.78096
XP_021973316.1	XP_022034230.1	1.4834	1.8992	0.78107
XP_021984766.1	XP_022010292.1	0.9732	1.2451	0.78162
XP_021985239.1	XP_022014870.1	1.0903	1.3946	0.7818
XP_021993621.2	XP_022010616.1	1.2156	1.554	0.78224
XP_021976022.1	XP_022010295.2	1.0607	1.3556	0.78246
XP_021981407.1	XP_022006927.1	1.204	1.5385	0.78258
XP_021986500.1	XP_022006668.1	1.0551	1.3473	0.78312
XP_021993620.1	XP_022021912.1	1.3354	1.7045	0.78346
XP_021969020.1	XP_021984766.1	1.3174	1.6814	0.78351
XP_022029622.1	XP_035838717.1	1.3135	1.6761	0.78366
XP_021987458.1	XP_022010101.1	1.3601	1.7322	0.78519
XP_022002415.1	XP_022026964.1	0.9342	1.1897	0.78524
XP_022000196.1	XP_022002417.1	1.0478	1.3343	0.78528
XP_021995117.1	XP_035835830.1	0.8997	1.1456	0.78535
XP_021972810.1	XP_022002415.1	0.6227	0.7925	0.78574
XP_021981388.1	XP_022034230.1	1.1528	1.467	0.78582
XP_021987458.1	XP_022029622.1	1.4731	1.8732	0.78641
XP_021993052.1	XP_022002420.1	0.8537	1.0853	0.7866
XP_021978842.1	XP_022006668.1	1.2637	1.6062	0.78676
XP_022002421.1	XP_022005608.1	0.9016	1.1459	0.78681
XP_021993222.1	XP_022010292.1	1.2868	1.6353	0.78689
XP_021993220.1	XP_021999258.1	1.4489	1.8411	0.78698
XP_021987457.1	XP_021993222.1	1.5867	2.0151	0.78741
XP_022001254.1	XP_022010616.1	1.3387	1.6997	0.78761
XP_021981388.1	XP_022000196.1	1.0734	1.3625	0.78782
XP_021973854.1	XP_035842399.1	1.489	1.89	0.78783
XP_021999258.1	XP_022002421.1	1.054	1.3377	0.78792
XP_021984981.2	XP_022013183.1	0.9635	1.2222	0.78833
XP_022010292.1	XP_022022487.2	0.8545	1.0839	0.78836
XP_021985239.1	XP_021986500.1	1.2292	1.5587	0.78861
XP_022026964.1	XP_022039085.1	1.3859	1.7572	0.7887
XP_021995116.1	XP_022026963.1	1.2572	1.5936	0.78891
XP_021988953.1	XP_021995116.1	1.5148	1.9199	0.789
XP_021993622.1	XP_022002416.1	1.1549	1.4637	0.78903
XP_021978807.1	XP_022026963.1	1.3009	1.6479	0.78943
XP_022004980.1	XP_022018622.1	1.3125	1.6625	0.78947
XP_022010295.2	XP_022035384.1	1.0872	1.3763	0.78994
XP_022006327.1	XP_022013560.1	1.1622	1.4706	0.79029
XP_021993222.1	XP_022026964.1	1.2152	1.5376	0.79032
XP_021989733.1	XP_022010291.1	1.002	1.2676	0.79047
XP_022002416.1	XP_022018622.1	0.8539	1.0802	0.7905
XP_022000196.1	XP_022005608.1	1.1281	1.4263	0.79093
XP_021988953.1	XP_022034230.1	1.2389	1.566	0.79112
XP_021987458.1	XP_022013239.1	1.4284	1.8055	0.79114
XP_021981626.1	XP_021989735.1	1.2574	1.5888	0.79141
XP_022006327.1	XP_022026964.1	1.3773	1.7403	0.79142
XP_021993222.1	XP_022004455.1	1.1261	1.4228	0.79147
XP_022009213.1	XP_022021912.1	1.2001	1.5162	0.79152
XP_022010296.1	XP_022021911.1	1.1782	1.4885	0.79154
XP_022006327.1	XP_022010295.2	1.147	1.449	0.79158
XP_021989737.1	XP_022010919.1	1.1727	1.4797	0.79253
XP_022026964.1	XP_022029622.1	1.1658	1.4706	0.79274
XP_022029740.1	XP_035842399.1	1.142	1.4401	0.793
XP_021995117.1	XP_022002418.1	0.8674	1.0938	0.79302
XP_021978807.1	XP_021993551.1	1.443	1.8196	0.79303
XP_021999258.1	XP_022004979.1	1.0803	1.3622	0.79306
XP_022010291.1	XP_022010292.1	0.5592	0.705	0.79319
XP_021993221.1	XP_022008276.2	1.4962	1.8862	0.79324
XP_022022835.1	XP_022029739.1	1.177	1.4835	0.79339
XP_022002420.1	XP_022013560.1	0.5536	0.6973	0.79392
XP_021989737.1	XP_022002421.1	1.0193	1.2838	0.79397
XP_021985239.1	XP_022010291.1	0.871	1.0969	0.79406
XP_021984981.2	XP_022033162.1	1.3972	1.7589	0.79436
XP_021981407.1	XP_022013183.1	0.863	1.0863	0.79444
XP_021973011.1	XP_022039074.2	1.1825	1.4884	0.79448
XP_021985239.1	XP_021993551.1	1.4665	1.8456	0.79459
XP_021981626.1	XP_022006926.1	1.272	1.6008	0.7946
XP_021973011.1	XP_022013560.1	1.0192	1.2824	0.79476
XP_021986500.1	XP_022022835.1	1.284	1.6149	0.7951
XP_022037775.1	XP_035838717.1	1.3396	1.6846	0.7952
XP_021999258.1	XP_022014870.1	1.2634	1.5881	0.79554
XP_022013183.1	XP_035833070.1	1.5514	1.9501	0.79555
XP_021976022.1	XP_022004979.1	1.244	1.5636	0.7956
XP_022000196.1	XP_022013183.1	1.3829	1.738	0.79568
XP_021995117.1	XP_022035384.1	1.2489	1.5695	0.79573
XP_021989737.1	XP_021993551.1	1.2788	1.607	0.79577
XP_021999258.1	XP_022034230.1	1.3769	1.7298	0.79599
XP_021968895.1	XP_022021912.1	1.2931	1.6239	0.79629
XP_022004980.1	XP_022008276.2	1.1477	1.4406	0.79668
XP_021993283.1	XP_022008276.2	1.2983	1.6295	0.79675
XP_022010919.1	XP_022013558.1	0.869	1.0905	0.79688
XP_021989733.1	XP_022002421.1	1.0431	1.3089	0.79693

XP_021986500.1	XP_022026964.1	1.1474	1.4397	0.79697
XP_022010294.1	XP_022013183.1	1.3088	1.6419	0.79713
XP_021993622.1	XP_022010616.1	1.4745	1.8497	0.79716
XP_022018622.1	XP_022021913.1	1.0193	1.2786	0.7972
XP_022018622.1	XP_022021914.1	1.0193	1.2786	0.7972
XP_022002421.1	XP_022029738.1	0.4832	0.606	0.79736
XP_021976022.1	XP_022013558.1	1.1057	1.3865	0.79748
XP_021985239.1	XP_022013560.1	0.8708	1.0912	0.79802
XP_022010296.1	XP_022013239.1	1.1184	1.4011	0.79823
XP_021978842.1	XP_035838717.1	1.1595	1.4522	0.79844
XP_021993622.1	XP_022010295.2	1.3309	1.6662	0.79876
XP_021981626.1	XP_022039074.2	1.4465	1.8107	0.79886
XP_021981388.1	XP_021989736.1	0.4191	0.5246	0.79889
XP_022008276.2	XP_022034230.1	1.5056	1.8842	0.79907
XP_021978842.1	XP_022002421.1	1.2494	1.5633	0.79921
XP_022008275.1	XP_022018622.1	1.1223	1.4039	0.79942
XP_022013182.1	XP_022015084.1	1.2638	1.5805	0.79962
XP_021999258.1	XP_022006668.1	1.5105	1.889	0.79963
XP_021986500.1	XP_022010295.2	0.9279	1.1601	0.79984
XP_021972810.1	XP_021989737.1	1.1887	1.4859	0.79999
XP_021993052.1	XP_022029740.1	0.8184	1.0229	0.80008
XP_022014870.1	XP_022029622.1	1.409	1.7609	0.80016
XP_022002418.1	XP_022026963.1	1.0124	1.2648	0.80044
XP_021978842.1	XP_022010294.1	1.1876	1.4836	0.80049
XP_021984766.1	XP_022010919.1	1.2534	1.5657	0.80054
XP_021988229.1	XP_022033162.1	1.0064	1.257	0.80064
XP_021989734.1	XP_022035384.1	1.1842	1.4789	0.80073
XP_021981407.1	XP_022008276.2	1.3389	1.6712	0.80116
XP_021993283.1	XP_022035384.1	1.2606	1.5733	0.80125
XP_022022487.2	XP_022037775.1	1.6114	2.0108	0.80137
XP_021981395.1	XP_035838717.1	0.7237	0.9028	0.80162
XP_022002419.1	XP_022018622.1	0.9527	1.1883	0.80173
XP_022006668.1	XP_022022487.2	1.4053	1.7525	0.80188
XP_021976022.1	XP_022039085.1	1.5266	1.9036	0.80195
XP_021993052.1	XP_022002415.1	0.8068	1.0059	0.80207
XP_021973316.1	XP_022013182.1	1.4207	1.7709	0.80225
XP_021985239.1	XP_022004980.1	1.3962	1.7399	0.80246
XP_021995116.1	XP_022006668.1	1.3336	1.6614	0.8027
XP_021978807.1	XP_021988229.1	1.0414	1.297	0.80293
XP_021984981.2	XP_022022835.1	0.9604	1.196	0.80301
XP_021995116.1	XP_022010291.1	1.2227	1.5224	0.80314
XP_022006927.1	XP_022021912.1	1.5358	1.9115	0.80345
XP_022002418.1	XP_022010295.2	0.5842	0.7271	0.80347
XP_021976022.1	XP_022002417.1	1.188	1.4783	0.80363
XP_022029738.1	XP_022034230.1	1.026	1.2763	0.80389
XP_022010296.1	XP_022021913.1	1.1759	1.4625	0.80403
XP_022010296.1	XP_022021914.1	1.1759	1.4625	0.80403
XP_022000196.1	XP_022013182.1	1.4293	1.7767	0.80447
XP_021973854.1	XP_022021913.1	1.7525	2.1781	0.8046
XP_021973854.1	XP_022021914.1	1.7525	2.1781	0.8046
XP_021984766.1	XP_022009213.1	0.9923	1.2327	0.80498
XP_021984981.2	XP_022005608.1	1.0763	1.337	0.80501
XP_021993052.1	XP_022010292.1	0.891	1.1068	0.80502
XP_021985239.1	XP_022037775.1	1.1934	1.482	0.80526
XP_022004979.1	XP_022009213.1	1.1436	1.42	0.80535
XP_021973854.1	XP_022013183.1	1.4443	1.7928	0.80561
XP_022006926.1	XP_022010919.1	1.238	1.5366	0.80567
XP_021984981.2	XP_022008275.1	1.0533	1.3073	0.80571
XP_022002415.1	XP_022026963.1	1.0025	1.2442	0.80574
XP_021986500.1	XP_021993220.1	1.1862	1.4721	0.80579
XP_022002420.1	XP_022005608.1	0.8333	1.0336	0.80621
XP_021973855.1	XP_022015084.1	1.5789	1.9584	0.80622
XP_021972810.1	XP_021981395.1	1.1547	1.4322	0.80624
XP_021993283.1	XP_022010919.1	1.4958	1.855	0.80636
XP_021993283.1	XP_022013183.1	1.4807	1.8356	0.80666
XP_021992525.1	XP_022013182.1	0.2767	0.343	0.80671
XP_021987458.1	XP_021992525.1	1.2394	1.5357	0.80706
XP_022004979.1	XP_022006926.1	1.2844	1.5913	0.80714
XP_022006327.1	XP_022010294.1	1.4349	1.7769	0.80753
XP_021973011.1	XP_035842399.1	1.5242	1.8871	0.80769
XP_021988953.1	XP_022021911.1	1.3509	1.6725	0.80771
XP_021981407.1	XP_021992525.1	0.8627	1.068	0.80777
XP_021993283.1	XP_022002420.1	1.2478	1.5445	0.8079
XP_021989735.1	XP_022000196.1	1.0796	1.336	0.80808
XP_021995116.1	XP_035838717.1	1.6012	1.9805	0.80848
XP_022002418.1	XP_022029740.1	0.3886	0.4806	0.80857
XP_021973316.1	XP_021988229.1	1.4209	1.7569	0.80875
XP_021973854.1	XP_022002421.1	1.1769	1.4552	0.80875
XP_021993220.1	XP_022006327.1	1.333	1.6479	0.80891
XP_021987458.1	XP_022010292.1	1.2371	1.5291	0.80904
XP_022009213.1	XP_022010295.2	1.0006	1.2367	0.80909
XP_021973854.1	XP_022013239.1	1.9848	2.4526	0.80926
XP_021973855.1	XP_021993621.2	1.1981	1.48	0.80953
XP_021985239.1	XP_021988953.1	1.1752	1.4513	0.80976
XP_021989736.1	XP_022022835.1	1.2771	1.5769	0.80988
XP_022000196.1	XP_022002415.1	1.0403	1.2845	0.80989
XP_021995117.1	XP_022005608.1	1.2754	1.5747	0.80993
XP_022029738.1	XP_022033162.1	0.8229	1.0158	0.8101

XP_021973011.1	XP_022035384.1	1.6463	2.031	0.81059
XP_022008276.2	XP_022039074.2	1.2866	1.587	0.81071
XP_022022835.1	XP_022039074.2	1.1153	1.3756	0.81077
XP_022000224.2	XP_022005608.1	1.3565	1.6722	0.81121
XP_021993622.1	XP_022026963.1	1.348	1.6616	0.81127
XP_021976022.1	XP_022026963.1	1.8677	2.3018	0.81141
XP_021989735.1	XP_022022835.1	1.3444	1.6568	0.81144
XP_022002418.1	XP_022013560.1	0.6306	0.7769	0.81169
XP_021988953.1	XP_022013558.1	1.0683	1.3156	0.81202
XP_021984766.1	XP_022004979.1	1.4285	1.7577	0.81271
XP_022002420.1	XP_022010616.1	0.8861	1.0894	0.81338
XP_021993221.1	XP_022010292.1	1.2996	1.5968	0.81388
XP_021969020.1	XP_021984981.2	1.3021	1.5995	0.81407
XP_022008276.2	XP_022009213.1	1.2781	1.5694	0.81439
XP_021993052.1	XP_022010294.1	0.9451	1.1602	0.8146
XP_021973855.1	XP_022008276.2	1.5825	1.9425	0.81467
XP_022008275.1	XP_022035552.1	1.33	1.6323	0.8148
XP_022035551.1	XP_022039085.1	1.1921	1.463	0.81483
XP_022002415.1	XP_022018622.1	0.8654	1.0618	0.81503
XP_021986500.1	XP_022006926.1	1.3262	1.6268	0.81522
XP_021981388.1	XP_035835830.1	1.0042	1.2314	0.81549
XP_022010101.1	XP_022022487.2	1.1594	1.4215	0.81562
XP_021993222.1	XP_022035552.1	1.38	1.6915	0.81584
XP_022010919.1	XP_022039074.2	1.2839	1.5737	0.81585
XP_022001254.1	XP_022013558.1	1.1032	1.3521	0.81592
XP_021993220.1	XP_022004980.1	1.1988	1.4691	0.81601
XP_022006927.1	XP_022021911.1	1.3923	1.7055	0.81636
XP_022013183.1	XP_022029738.1	1.2317	1.5083	0.81661
XP_021973855.1	XP_022009213.1	1.6838	2.0609	0.81702
XP_021987458.1	XP_022021912.1	1.2637	1.5465	0.81714
XP_021993052.1	XP_022010295.2	0.8503	1.0404	0.81728
XP_022002419.1	XP_022010919.1	1.0203	1.2482	0.81742
XP_022029738.1	XP_035842399.1	1.2821	1.5683	0.81751
XP_021978842.1	XP_022002419.1	1.1239	1.3742	0.81786
XP_022013183.1	XP_022013239.1	1.4496	1.7724	0.81787
XP_021978807.1	XP_022035384.1	1.5146	1.8511	0.81822
XP_022001254.1	XP_022018622.1	1.3667	1.6699	0.81843
XP_022002419.1	XP_022010295.2	0.6405	0.7824	0.81863
XP_021993222.1	XP_021995116.1	1.6018	1.9566	0.81867
XP_022014870.1	XP_022018622.1	1.2831	1.5673	0.81867
XP_021969020.1	XP_022039074.2	1.3637	1.6654	0.81884
XP_021987457.1	XP_022021912.1	1.2625	1.5416	0.81895
XP_021981388.1	XP_022008276.2	1.3499	1.6479	0.81916
XP_022002415.1	XP_022002421.1	0.1807	0.2205	0.8195
XP_021993621.2	XP_022021911.1	1.3508	1.6479	0.81971
XP_021993621.2	XP_022021913.1	1.3508	1.6479	0.81971
XP_021993621.2	XP_022021914.1	1.3508	1.6479	0.81971
XP_021973855.1	XP_021989733.1	1.8633	2.273	0.81975
XP_022002421.1	XP_022018622.1	0.9819	1.1974	0.82003
XP_021995116.1	XP_022001254.1	1.4371	1.752	0.82026
XP_021987458.1	XP_021993222.1	1.5838	1.9298	0.82071
XP_021972810.1	XP_035838717.1	1.1945	1.4542	0.82141
XP_022039085.1	XP_035835830.1	0.6222	0.7574	0.82149
XP_022035552.1	XP_022039085.1	1.192	1.4496	0.8223
XP_022010292.1	XP_022010919.1	0.8961	1.0888	0.82302
XP_022002416.1	XP_022026964.1	0.9545	1.1592	0.82341
XP_021972810.1	XP_021989733.1	1.2071	1.4659	0.82345
XP_022002415.1	XP_022013560.1	0.6273	0.7617	0.82355
XP_021984766.1	XP_021984981.2	0.8146	0.9886	0.82399
XP_021972810.1	XP_022002418.1	0.6424	0.7795	0.82412
XP_021995117.1	XP_022004455.1	1.0736	1.3027	0.82413
XP_021987457.1	XP_021989736.1	1.1622	1.4092	0.82472
XP_022002419.1	XP_035842399.1	1.2967	1.572	0.82487
XP_021993622.1	XP_022006668.1	1.498	1.8149	0.82539
XP_021978807.1	XP_021993622.1	1.4088	1.7065	0.82555
XP_022010919.1	XP_022029738.1	0.9627	1.1661	0.82557
XP_021992525.1	XP_022010294.1	1.1606	1.4056	0.8257
XP_022013182.1	XP_035833070.1	1.5626	1.8915	0.82612
XP_021988953.1	XP_021992525.1	1.1764	1.4236	0.82636
XP_022021914.1	XP_022022835.1	1.4418	1.7443	0.82658
XP_021973316.1	XP_022010292.1	1.1497	1.3909	0.82659
XP_021993283.1	XP_022034230.1	1.5815	1.9129	0.82676
XP_022004979.1	XP_035838717.1	1.165	1.4091	0.82677
XP_022010295.2	XP_022013239.1	1.0031	1.213	0.82696
XP_021987458.1	XP_021993620.1	1.0296	1.2447	0.82719
XP_021992525.1	XP_022005608.1	1.2776	1.5443	0.8273
XP_021985239.1	XP_022008276.2	1.2704	1.5353	0.82746
XP_021993620.1	XP_022004980.1	1.5367	1.8567	0.82765
XP_021972810.1	XP_021981388.1	1.15	1.3893	0.82775
XP_021993551.1	XP_022010616.1	1.7094	2.0645	0.828
XP_021989736.1	XP_022008275.1	1.1255	1.3587	0.82837
XP_022002420.1	XP_035833070.1	1.2468	1.5034	0.82932
XP_021993551.1	XP_022021913.1	1.3992	1.6857	0.83004
XP_021993551.1	XP_022021914.1	1.3992	1.6857	0.83004
XP_022000224.2	XP_022002418.1	0.8283	0.9978	0.83013
XP_022010291.1	XP_022022835.1	1.0407	1.2535	0.83024
XP_021989735.1	XP_022010295.2	1.1353	1.3674	0.83026
XP_022010291.1	XP_022021912.1	1.4066	1.6938	0.83044

XP_021972810.1	XP_022029740.1	0.6771	0.8153	0.83049
XP_021978842.1	XP_022010291.1	1.0967	1.3205	0.83052
XP_021993283.1	XP_022010296.1	1.3687	1.6479	0.83057
XP_021988229.1	XP_022004980.1	1.3889	1.6722	0.83058
XP_021973011.1	XP_022000224.2	1.1504	1.3844	0.83097
XP_022002421.1	XP_022013560.1	0.6604	0.7947	0.83101
XP_021978807.1	XP_022002416.1	1.0018	1.205	0.83137
XP_021993551.1	XP_022021911.1	1.3841	1.6647	0.83144
XP_021993052.1	XP_021999258.1	1.2463	1.4987	0.83159
XP_021981388.1	XP_022006926.1	1.5427	1.8547	0.83178
XP_021993621.2	XP_022002418.1	0.9697	1.1653	0.83215
XP_021973854.1	XP_022021911.1	1.7174	2.0631	0.83244
XP_021973854.1	XP_021993222.1	1.7268	2.0743	0.83247
XP_022039085.1	XP_035842399.1	1.4293	1.7165	0.83268
XP_022009213.1	XP_022010291.1	1.158	1.3903	0.83291
XP_021976022.1	XP_021989733.1	1.1852	1.4227	0.83306
XP_021984766.1	XP_022010291.1	1.245	1.4938	0.83344
XP_022010295.2	XP_022026964.1	0.8637	1.0356	0.83401
XP_022013239.1	XP_022035384.1	1.5109	1.8115	0.83406
XP_021981626.1	XP_021993220.1	1.3983	1.6761	0.83426
XP_022002415.1	XP_022010295.2	0.5604	0.6717	0.8343
XP_021978807.1	XP_022000196.1	1.2889	1.5447	0.8344
XP_022009213.1	XP_022029622.1	1.3527	1.6208	0.83459
XP_021978807.1	XP_022002420.1	1.0355	1.2406	0.83468
XP_022006327.1	XP_022013183.1	1.2276	1.4706	0.83476
XP_022009213.1	XP_022010296.1	1.0166	1.2178	0.83478
XP_022006927.1	XP_022037775.1	1.0295	1.2332	0.83482
XP_022004980.1	XP_022013558.1	1.0953	1.3115	0.83515
XP_022022487.2	XP_022035384.1	1.5904	1.9039	0.83534
XP_021976022.1	XP_021995117.1	1.2526	1.4995	0.83535
XP_022006927.1	XP_022039085.1	1.0566	1.2648	0.83539
XP_021972810.1	XP_021978807.1	1.0726	1.2839	0.83542
XP_021973011.1	XP_022010294.1	1.0372	1.2411	0.83571
XP_021978807.1	XP_022021913.1	1.2037	1.4397	0.83608
XP_021978807.1	XP_022021914.1	1.2037	1.4397	0.83608
XP_021993621.2	XP_022014870.1	1.3942	1.6675	0.8361
XP_022026964.1	XP_022034230.1	1.2709	1.5195	0.83639
XP_021978807.1	XP_021999258.1	1.3541	1.6186	0.83659
XP_021988953.1	XP_022037775.1	1.3012	1.555	0.83678
XP_021992525.1	XP_022013183.1	0.3048	0.3642	0.8369
XP_021992525.1	XP_035842399.1	1.5094	1.8034	0.83697
XP_021993221.1	XP_022021912.1	1.5067	1.8	0.83706
XP_021981388.1	XP_022037775.1	1.2652	1.5112	0.83722
XP_021973316.1	XP_021987457.1	1.4197	1.6956	0.83728
XP_021976022.1	XP_022010292.1	1.1809	1.4103	0.83734
XP_021989733.1	XP_022022487.2	1.3151	1.5704	0.83743
XP_022002420.1	XP_022013239.1	0.9762	1.1655	0.83758
XP_021989737.1	XP_022006927.1	1.2821	1.5307	0.83759
XP_021981407.1	XP_022037775.1	1.2673	1.5128	0.83772
XP_021987457.1	XP_022002418.1	1.1788	1.4069	0.83787
XP_021973316.1	XP_021993220.1	1.3928	1.6623	0.83788
XP_021993283.1	XP_021999258.1	1.4119	1.6846	0.83812
XP_021988229.1	XP_022021912.1	1.2718	1.5174	0.83814
XP_022018622.1	XP_022034230.1	1.2421	1.4815	0.83841
XP_021999258.1	XP_022021912.1	1.3593	1.6211	0.8385
XP_022001254.1	XP_022033162.1	1.1814	1.4089	0.83853
XP_021995116.1	XP_022022835.1	1.3857	1.6525	0.83855
XP_022000196.1	XP_035835830.1	1.1042	1.3165	0.83874
XP_021968895.1	XP_021993221.1	1.297	1.5463	0.83878
XP_021969020.1	XP_021973011.1	1.5864	1.8912	0.83883
XP_021985239.1	XP_022006927.1	1.2934	1.5419	0.83884
XP_021987458.1	XP_022035384.1	1.7469	2.0824	0.83889
XP_021973011.1	XP_022010296.1	0.9649	1.1496	0.83934
XP_022001254.1	XP_022026963.1	1.4234	1.6957	0.83942
XP_021973316.1	XP_022000224.2	1.1962	1.425	0.83944
XP_021981395.1	XP_022013239.1	1.2649	1.5068	0.83946
XP_021986500.1	XP_021989734.1	0.9763	1.1629	0.83954
XP_022033162.1	XP_035842399.1	1.2693	1.5119	0.83954
XP_021972810.1	XP_022014870.1	1.1766	1.4012	0.83971
XP_021993551.1	XP_022009213.1	1.3838	1.6479	0.83974
XP_021978807.1	XP_022004455.1	1.0201	1.2144	0.84
XP_022000224.2	XP_035838717.1	1.0455	1.2443	0.84023
XP_021988229.1	XP_022013239.1	1.3774	1.6392	0.84029
XP_022000196.1	XP_022039085.1	1.6142	1.92	0.84073
XP_021988229.1	XP_022006926.1	1.0336	1.2293	0.8408
XP_021981388.1	XP_022035552.1	1.0001	1.1894	0.84084
XP_021978807.1	XP_022010919.1	1.3101	1.5576	0.8411
XP_021993222.1	XP_022010919.1	1.3759	1.6358	0.84112
XP_021993283.1	XP_035838717.1	1.5127	1.7984	0.84114
XP_021981407.1	XP_021995116.1	1.3533	1.6075	0.84187
XP_022010291.1	XP_035838717.1	1.1209	1.3314	0.8419
XP_021993283.1	XP_022013182.1	1.5158	1.8003	0.84197
XP_021988953.1	XP_022010296.1	1.1114	1.3199	0.84203
XP_022006926.1	XP_022013183.1	1.5898	1.8875	0.84228
XP_021972810.1	XP_022002419.1	0.5837	0.693	0.84228
XP_022000196.1	XP_022002418.1	1.0258	1.2178	0.84234
XP_021993222.1	XP_022035551.1	1.3793	1.6366	0.84278
XP_022021911.1	XP_022022835.1	1.4433	1.7121	0.843

XP_021984981.2	XP_022004980.1	1.1636	1.3803	0.84301
XP_021981395.1	XP_022009213.1	0.7244	0.8591	0.84321
XP_021988953.1	XP_022010294.1	1.1596	1.3751	0.84328
XP_021992525.1	XP_022015084.1	1.212	1.4372	0.84331
XP_021987457.1	XP_022035384.1	1.7477	2.0716	0.84365
XP_022004979.1	XP_022013182.1	1.2467	1.4777	0.84368
XP_021973316.1	XP_022002421.1	1.0136	1.2011	0.84389
XP_021989736.1	XP_022039085.1	1.1363	1.3464	0.84395
XP_021993622.1	XP_022035384.1	1.6454	1.9493	0.8441
XP_021981626.1	XP_022029622.1	1.4341	1.6978	0.84468
XP_021987458.1	XP_022002418.1	1.1965	1.4165	0.84469
XP_022021912.1	XP_022037775.1	1.2031	1.424	0.84487
XP_021992525.1	XP_022034230.1	1.1836	1.4005	0.84513
XP_021993551.1	XP_022004455.1	1.2017	1.4218	0.8452
XP_021973316.1	XP_021995117.1	1.2185	1.4414	0.84536
XP_021981407.1	XP_021999258.1	1.1004	1.3015	0.84549
XP_021993551.1	XP_021999258.1	1.5294	1.8089	0.84549
XP_022000196.1	XP_022018622.1	1.2747	1.5073	0.84568
XP_021988229.1	XP_021999258.1	1.4179	1.6765	0.84575
XP_022010101.1	XP_022014870.1	1.4108	1.6681	0.84575
XP_022002416.1	XP_022010616.1	0.8563	1.0124	0.84581
XP_022018622.1	XP_035838717.1	1.3449	1.5899	0.8459
XP_021969020.1	XP_022021912.1	1.3171	1.5569	0.84598
XP_022006926.1	XP_022034230.1	0.8116	0.959	0.8463
XP_021973854.1	XP_022006927.1	1.3765	1.626	0.84656
XP_021984981.2	XP_021987457.1	1.0925	1.2905	0.84657
XP_021993551.1	XP_022034230.1	1.6076	1.8986	0.84673
XP_022026964.1	XP_022033162.1	1.0616	1.2532	0.84711
XP_022006327.1	XP_022022835.1	1.5611	1.8426	0.84723
XP_022018622.1	XP_022029740.1	1.0088	1.1906	0.8473
XP_021992525.1	XP_022039074.2	1.3779	1.6262	0.84731
XP_021993622.1	XP_022002421.1	1.2366	1.4594	0.84733
XP_021993220.1	XP_022029622.1	1.709	2.0169	0.84734
XP_022006926.1	XP_022010616.1	1.1104	1.3102	0.8475
XP_022018622.1	XP_022021911.1	1.0232	1.2071	0.84765
XP_022014870.1	XP_035835830.1	1.1902	1.4039	0.84778
XP_022002418.1	XP_022026964.1	0.9591	1.1313	0.84779
XP_021995117.1	XP_022006327.1	1.3123	1.5476	0.84796
XP_021989736.1	XP_022008276.2	1.4162	1.67	0.84802
XP_022006668.1	XP_022021912.1	1.3263	1.5636	0.84823
XP_021989734.1	XP_022039074.2	1.1134	1.3126	0.84824
XP_021981388.1	XP_035838717.1	0.6625	0.781	0.84827
XP_022002416.1	XP_022002421.1	0.1834	0.2162	0.84829
XP_021993221.1	XP_022006926.1	1.2522	1.4753	0.84878
XP_022006927.1	XP_022010101.1	0.5912	0.6965	0.84882
XP_021984981.2	XP_021988953.1	1.2873	1.5163	0.84897
XP_022014870.1	XP_022035551.1	0.9756	1.149	0.84909
XP_022014870.1	XP_022035552.1	0.9756	1.149	0.84909
XP_021988229.1	XP_022006927.1	1.1115	1.3089	0.84919
XP_021987457.1	XP_022013239.1	1.4127	1.6634	0.84928
XP_021993222.1	XP_035833070.1	1.3862	1.6318	0.84949
XP_021976022.1	XP_021984766.1	1.3331	1.5692	0.84954
XP_021981626.1	XP_022002418.1	0.9811	1.1546	0.84973
XP_021993221.1	XP_022000196.1	1.4284	1.681	0.84973
XP_021993621.2	XP_021993622.1	0.9354	1.1008	0.84975
XP_021969020.1	XP_022006327.1	1.9165	2.2552	0.84981
XP_022006668.1	XP_022029738.1	1.1943	1.4051	0.84998
XP_022008276.2	XP_022013183.1	1.3567	1.596	0.85006
XP_021981388.1	XP_021981407.1	0.2495	0.2935	0.85009
XP_021976022.1	XP_021978842.1	1.2712	1.4953	0.85013
XP_021969020.1	XP_022034230.1	1.4713	1.7302	0.85036
XP_021976022.1	XP_021987457.1	1.5424	1.8136	0.85046
XP_022022835.1	XP_035842399.1	1.6393	1.9275	0.85048
XP_022039085.1	XP_035833070.1	1.5731	1.8496	0.85051
XP_021984981.2	XP_021987458.1	1.1041	1.298	0.85062
XP_021993221.1	XP_022010294.1	1.4019	1.6479	0.85072
XP_022013182.1	XP_022021911.1	1.3985	1.6437	0.85082
XP_021993052.1	XP_022013560.1	0.9468	1.1126	0.85098
XP_021989737.1	XP_035835830.1	1.0194	1.1979	0.85099
XP_021981626.1	XP_022000224.2	1.1708	1.3755	0.85118
XP_021993621.2	XP_022001254.1	1.3608	1.5987	0.85119
XP_021993283.1	XP_022010616.1	1.1887	1.3961	0.85144
XP_022021913.1	XP_022022835.1	1.4665	1.7215	0.85187
XP_021993551.1	XP_022029740.1	1.1714	1.3744	0.8523
XP_022018622.1	XP_035842399.1	1.5786	1.852	0.85238
XP_021973011.1	XP_021993283.1	1.5036	1.7635	0.85262
XP_021981626.1	XP_021993222.1	1.505	1.7646	0.85288
XP_021993621.2	XP_022002416.1	0.9586	1.1239	0.85292
XP_021993621.2	XP_022002417.1	0.9586	1.1239	0.85292
XP_021999258.1	XP_022001254.1	1.5503	1.8172	0.85313
XP_021993551.1	XP_022000196.1	1.3762	1.613	0.85319
XP_022035384.1	XP_035833070.1	1.9719	2.3111	0.85323
XP_021988229.1	XP_022010919.1	1.1538	1.3521	0.85334
XP_021989735.1	XP_022022487.2	1.2554	1.4706	0.85367
XP_022008276.2	XP_022013182.1	1.3474	1.5781	0.85381
XP_022013182.1	XP_022037775.1	1.3971	1.6363	0.85382
XP_022010294.1	XP_022022835.1	1.0831	1.2678	0.85431
XP_021989735.1	XP_022029622.1	1.2562	1.47	0.85456

XP_021995116.1	XP_022004980.1	1.3769	1.6103	0.85506
XP_021981395.1	XP_021989735.1	0.4371	0.5109	0.85555
XP_021973316.1	XP_022014870.1	1.1657	1.3623	0.85569
XP_021981388.1	XP_022006327.1	1.4939	1.7443	0.85645
XP_021985239.1	XP_022021912.1	1.2354	1.4423	0.85655
XP_021993551.1	XP_022039085.1	1.3591	1.5856	0.85715
XP_022018622.1	XP_022029738.1	0.9539	1.1127	0.85728
XP_021988953.1	XP_022029740.1	1.1239	1.3108	0.85742
XP_021993220.1	XP_022039074.2	1.3955	1.6275	0.85745
XP_022002417.1	XP_022010616.1	0.8585	1.001	0.85764
XP_022021912.1	XP_022029740.1	1.3241	1.5434	0.85791
XP_021993221.1	XP_021999258.1	1.2629	1.472	0.85795
XP_021995116.1	XP_022034230.1	1.4908	1.7371	0.85821
XP_022000196.1	XP_022002420.1	1.0938	1.2745	0.85822
XP_022004979.1	XP_022026964.1	1.0321	1.2023	0.85844
XP_022002418.1	XP_035842399.1	1.1379	1.3246	0.85905
XP_022010294.1	XP_022010919.1	0.9763	1.1363	0.85919
XP_021989733.1	XP_021989736.1	0.1796	0.209	0.85933
XP_021986500.1	XP_022029738.1	0.89	1.0356	0.85941
XP_021993283.1	XP_022000196.1	1.7269	2.0093	0.85945
XP_022002419.1	XP_022002421.1	0.1739	0.2023	0.85961
XP_022008275.1	XP_022035551.1	1.3071	1.5199	0.85999
XP_021976022.1	XP_022029738.1	1.203	1.3986	0.86015
XP_022008276.2	XP_022022487.2	1.6215	1.8842	0.86058
XP_022009213.1	XP_022021913.1	1.2668	1.4714	0.86095
XP_022009213.1	XP_022021914.1	1.2668	1.4714	0.86095
XP_021992525.1	XP_022010295.2	1.3738	1.5956	0.86099
XP_021989733.1	XP_022022835.1	1.2602	1.4635	0.86109
XP_022009213.1	XP_022021911.1	1.2617	1.4651	0.86117
XP_021972810.1	XP_022000196.1	1.3713	1.592	0.86137
XP_021984766.1	XP_035838717.1	0.9872	1.1458	0.86158
XP_021973855.1	XP_021993052.1	1.5503	1.7984	0.86204
XP_021993052.1	XP_022039074.2	1.0879	1.262	0.86204
XP_021992525.1	XP_022021911.1	1.4371	1.6666	0.86229
XP_021989735.1	XP_022029740.1	1.2686	1.4709	0.86247
XP_021993220.1	XP_022000196.1	1.5186	1.7607	0.8625
XP_021973854.1	XP_022000224.2	1.0745	1.2454	0.86278
XP_022021912.1	XP_022033162.1	1.2336	1.4291	0.8632
XP_021976022.1	XP_021988229.1	1.3432	1.5557	0.86341
XP_022002419.1	XP_022013239.1	1.0228	1.1845	0.86349
XP_021993221.1	XP_022008275.1	1.3759	1.5933	0.86355
XP_022002416.1	XP_035842399.1	1.1341	1.3132	0.86362
XP_022006668.1	XP_022006926.1	1.1786	1.3646	0.8637
XP_022039074.2	XP_035838717.1	1.1674	1.3511	0.86404
XP_021986500.1	XP_021993621.2	1.3763	1.5927	0.86413
XP_021981388.1	XP_022035551.1	0.9953	1.1517	0.8642
XP_021981626.1	XP_022006927.1	1.4112	1.632	0.86471
XP_021976022.1	XP_022009213.1	1.2118	1.4013	0.86477
XP_021999258.1	XP_022029738.1	1.2413	1.4352	0.8649
XP_021968895.1	XP_021981407.1	1.2658	1.4633	0.86503
XP_021973316.1	XP_021987458.1	1.4295	1.6525	0.86505
XP_021988229.1	XP_022039074.2	0.8684	1.0028	0.86598
XP_021972810.1	XP_021993222.1	1.5275	1.7635	0.86618
XP_022006926.1	XP_022021911.1	1.7357	2.0033	0.86642
XP_022002415.1	XP_022029739.1	0.445	0.5134	0.86677
XP_021981626.1	XP_022035552.1	1.3012	1.5009	0.86695
XP_021999258.1	XP_022013560.1	1.2045	1.3893	0.86698
XP_021993621.2	XP_022002415.1	0.9952	1.1475	0.86728
XP_022013182.1	XP_022021913.1	1.4262	1.644	0.86752
XP_021981407.1	XP_022006327.1	1.5363	1.7709	0.86752
XP_021985239.1	XP_022026964.1	1.1918	1.3733	0.86784
XP_021992525.1	XP_021993052.1	1.4486	1.6683	0.86831
XP_021993621.2	XP_022000196.1	1.4882	1.7139	0.86831
XP_021993551.1	XP_022002420.1	1.1288	1.2998	0.86844
XP_021976022.1	XP_022022835.1	1.3076	1.5056	0.86849
XP_021973316.1	XP_021989735.1	1.3031	1.5001	0.86868
XP_021993283.1	XP_022021912.1	1.7598	2.0254	0.86887
XP_022002417.1	XP_022029740.1	0.399	0.4591	0.86909
XP_021999258.1	XP_022026964.1	1.5241	1.7532	0.86932
XP_021993551.1	XP_022039074.2	1.3373	1.5382	0.86939
XP_022033162.1	XP_022035551.1	1.8589	2.1379	0.8695
XP_022033162.1	XP_022035552.1	1.8589	2.1379	0.8695
XP_022006926.1	XP_035833070.1	1.2309	1.4153	0.86971
XP_021989734.1	XP_022008276.2	1.2929	1.4863	0.86988
XP_022013560.1	XP_022039074.2	0.8175	0.9396	0.87005
XP_021993551.1	XP_022022835.1	1.2635	1.452	0.87018
XP_021989737.1	XP_021995116.1	1.256	1.4426	0.87065
XP_021995117.1	XP_022010919.1	1.2583	1.4447	0.87098
XP_021989737.1	XP_035838717.1	0.7256	0.8328	0.87128
XP_021968895.1	XP_021984766.1	1.2385	1.4214	0.87132
XP_022013239.1	XP_022034230.1	1.3674	1.569	0.87151
XP_022000224.2	XP_022009213.1	1.0787	1.237	0.87203
XP_021988953.1	XP_022039085.1	1.2899	1.4788	0.87226
XP_022026964.1	XP_035838717.1	1.2013	1.3772	0.87228
XP_021999258.1	XP_035833070.1	1.7222	1.9742	0.87235
XP_021987458.1	XP_022037775.1	1.6027	1.8369	0.8725
XP_021999258.1	XP_022021911.1	1.407	1.6123	0.87267
XP_021999258.1	XP_022021913.1	1.407	1.6123	0.87267

XP_021999258.1	XP_022021914.1	1.407	1.6123	0.87267
XP_021984766.1	XP_021999258.1	1.1786	1.3499	0.8731
XP_021973854.1	XP_021981388.1	1.5636	1.7895	0.87376
XP_022004980.1	XP_022015084.1	1.2316	1.4095	0.87379
XP_021981626.1	XP_022005608.1	1.078	1.2332	0.87415
XP_021985239.1	XP_022005608.1	1.1037	1.2624	0.87429
XP_022021912.1	XP_022026963.1	1.5805	1.8075	0.87441
XP_021978842.1	XP_022002416.1	1.1113	1.2709	0.87442
XP_022010919.1	XP_022033162.1	1.5329	1.753	0.87444
XP_021992525.1	XP_022004979.1	1.3832	1.5817	0.8745
XP_022002420.1	XP_035842399.1	1.1101	1.269	0.87478
XP_022009213.1	XP_022035551.1	1.1656	1.3322	0.87494
XP_022004980.1	XP_022026964.1	1.3178	1.5053	0.87544
XP_021993622.1	XP_022029739.1	1.4944	1.7065	0.87571
XP_022009213.1	XP_022026964.1	1.19	1.3584	0.87603
XP_021992525.1	XP_022035384.1	1.3079	1.4926	0.87626
XP_022022487.2	XP_022039074.2	1.2574	1.4347	0.87642
XP_022022835.1	XP_022033162.1	1.3791	1.5733	0.87657
XP_021978807.1	XP_022021911.1	1.1994	1.3681	0.87669
XP_021987458.1	XP_022013558.1	1.3135	1.498	0.87684
XP_022004979.1	XP_022004980.1	0.8773	1.0005	0.87686
XP_021988229.1	XP_022018622.1	1.4611	1.6662	0.87691
XP_021988953.1	XP_022029739.1	1.167	1.3305	0.87711
XP_021973854.1	XP_022002418.1	1.167	1.3296	0.87771
XP_022002416.1	XP_022029740.1	0.3851	0.4387	0.87782
XP_022010919.1	XP_022014870.1	1.2683	1.4447	0.8779
XP_021973316.1	XP_022022835.1	1.2599	1.4348	0.8781
XP_022026964.1	XP_022039074.2	1.065	1.2126	0.87828
XP_021995117.1	XP_022013239.1	1.2887	1.467	0.87846
XP_021976022.1	XP_035838717.1	1.2391	1.4104	0.87855
XP_022009213.1	XP_022035552.1	1.1629	1.3236	0.87859
XP_021987457.1	XP_021993620.1	1.0379	1.1812	0.87868
XP_021973854.1	XP_022029739.1	1.3253	1.5082	0.87873
XP_021989737.1	XP_022039085.1	1.2402	1.4113	0.87876
XP_021987457.1	XP_022002415.1	1.1991	1.3645	0.87878
XP_022029622.1	XP_022037775.1	1.096	1.2468	0.87905
XP_021989734.1	XP_021993220.1	1.5503	1.7635	0.8791
XP_021989733.1	XP_022013239.1	1.4419	1.6393	0.87958
XP_021973011.1	XP_022035551.1	1.4446	1.6422	0.87967
XP_021973011.1	XP_022035552.1	1.4446	1.6422	0.87967
XP_021993220.1	XP_022021912.1	1.8047	2.0514	0.87974
XP_021973855.1	XP_022010919.1	1.3539	1.5381	0.88024
XP_022021913.1	XP_022029740.1	1.2716	1.4444	0.88037
XP_022021914.1	XP_022029740.1	1.2716	1.4444	0.88037
XP_021989737.1	XP_021993222.1	1.4433	1.6393	0.88044
XP_021976022.1	XP_022013239.1	1.4582	1.6556	0.88077
XP_022035384.1	XP_022037775.1	1.2243	1.3895	0.88111
XP_021976022.1	XP_022034230.1	1.7521	1.9883	0.88121
XP_021989733.1	XP_022035384.1	1.1863	1.3462	0.88122
XP_022021911.1	XP_022029740.1	1.2546	1.4235	0.88135
XP_022010101.1	XP_022022835.1	1.4986	1.6994	0.88184
XP_022013182.1	XP_022021914.1	1.4392	1.6317	0.88202
XP_021981395.1	XP_022035552.1	1.1123	1.2608	0.88222
XP_021989733.1	XP_022039085.1	1.2945	1.4673	0.88223
XP_021993220.1	XP_021993222.1	1.0877	1.2328	0.8823
XP_022029739.1	XP_022035384.1	1.4991	1.6988	0.88245
XP_022013183.1	XP_022037775.1	1.372	1.5547	0.88249
XP_021992525.1	XP_022022835.1	1.2392	1.404	0.88262
XP_021989734.1	XP_022006927.1	1.2194	1.3813	0.88279
XP_021988953.1	XP_021995117.1	1.3511	1.5304	0.88284
XP_021988953.1	XP_021993620.1	1.2818	1.4511	0.88333
XP_022006327.1	XP_022010296.1	1.2345	1.3974	0.88343
XP_021978842.1	XP_021993220.1	1.4209	1.608	0.88364
XP_021968895.1	XP_021978807.1	1.1054	1.2509	0.88368
XP_021987458.1	XP_022015084.1	1.0704	1.2102	0.88448
XP_022004980.1	XP_022010291.1	1.3809	1.5612	0.88451
XP_021972810.1	XP_021993551.1	1.2778	1.4436	0.88515
XP_021973854.1	XP_021993220.1	1.4894	1.6826	0.88518
XP_021978842.1	XP_022018622.1	1.3086	1.4783	0.88521
XP_021993283.1	XP_022002419.1	1.2384	1.3988	0.88533
XP_021972810.1	XP_022029739.1	0.814	0.9194	0.88536
XP_022010291.1	XP_022022487.2	1.1913	1.3455	0.8854
XP_021973316.1	XP_022010291.1	1.2326	1.392	0.88549
XP_021999258.1	XP_022010294.1	1.1743	1.3259	0.88566
XP_021984766.1	XP_022010296.1	0.9961	1.1246	0.88574
XP_021987458.1	XP_022002415.1	1.2173	1.374	0.88595
XP_021973855.1	XP_022037775.1	1.6617	1.8756	0.88596
XP_022010292.1	XP_022021911.1	1.3246	1.4941	0.88655
XP_022010292.1	XP_022021913.1	1.3246	1.4941	0.88655
XP_022010292.1	XP_022021914.1	1.3246	1.4941	0.88655
XP_022002417.1	XP_022013239.1	1.0813	1.2192	0.88689
XP_021984981.2	XP_021986500.1	1.1318	1.2753	0.88748
XP_021973855.1	XP_021993283.1	2.0738	2.3367	0.88749
XP_021993551.1	XP_035833070.1	1.9348	2.1793	0.88781
XP_021987457.1	XP_022010295.2	1.3117	1.4772	0.88796
XP_021993221.1	XP_022039074.2	1.154	1.2995	0.88803
XP_021987457.1	XP_022015084.1	1.0834	1.2196	0.88832
XP_021993621.2	XP_021995116.1	0.6285	0.7072	0.88872

XP_022026963.1	XP_035833070.1	1.5206	1.7095	0.8895
XP_022005608.1	XP_022022835.1	1.1828	1.3295	0.88966
XP_021993222.1	XP_022004979.1	1.4952	1.68	0.89
XP_021986500.1	XP_022029622.1	1.1468	1.2882	0.89023
XP_022006668.1	XP_022013182.1	1.2911	1.4501	0.89035
XP_021973855.1	XP_021995116.1	1.7297	1.9427	0.89036
XP_021984981.2	XP_021989736.1	0.7907	0.888	0.89043
XP_022002415.1	XP_022029740.1	0.3674	0.4126	0.89045
XP_022021912.1	XP_022039085.1	1.3842	1.5539	0.89079
XP_021973316.1	XP_022008275.1	1.6434	1.8445	0.89097
XP_021993283.1	XP_022006926.1	1.422	1.5956	0.8912
XP_021972810.1	XP_022002421.1	0.6313	0.7079	0.89179
XP_021976022.1	XP_021987458.1	1.4778	1.6571	0.8918
XP_021999258.1	XP_022004980.1	1.4701	1.6479	0.89211
XP_021999258.1	XP_022006926.1	1.2854	1.4397	0.89282
XP_021993622.1	XP_022022835.1	1.7201	1.9255	0.89333
XP_021999258.1	XP_022035552.1	1.3168	1.4736	0.89359
XP_021985239.1	XP_035833070.1	1.2352	1.382	0.89378
XP_021972810.1	XP_022002417.1	0.685	0.7664	0.89379
XP_021973855.1	XP_022013239.1	1.8142	2.029	0.89414
XP_022004980.1	XP_022010296.1	1.1107	1.2419	0.89436
XP_021981626.1	XP_022035551.1	1.3219	1.4778	0.89451
XP_021972810.1	XP_021989734.1	1.3379	1.4948	0.89504
XP_021973854.1	XP_022010296.1	1.275	1.4245	0.89505
XP_021973316.1	XP_022010294.1	1.1013	1.2304	0.89507
XP_021978807.1	XP_022002417.1	1.0058	1.1236	0.89516
XP_021993620.1	XP_022013239.1	0.8293	0.9263	0.89528
XP_021978842.1	XP_022029739.1	1.1292	1.2611	0.89541
XP_021981626.1	XP_021999258.1	1.6693	1.8637	0.89569
XP_021978807.1	XP_022010101.1	1.0001	1.1161	0.89607
XP_021992525.1	XP_022029740.1	1.3484	1.5041	0.89648
XP_021987457.1	XP_021999258.1	1.5532	1.7324	0.89656
XP_021984766.1	XP_021986500.1	1.2834	1.4313	0.89667
XP_021972810.1	XP_022021912.1	1.3281	1.4811	0.8967
XP_021992525.1	XP_022021914.1	1.4666	1.6345	0.89728
XP_021993551.1	XP_022004980.1	1.5677	1.7459	0.89793
XP_021973855.1	XP_021993622.1	2.3554	2.6224	0.89818
XP_021989735.1	XP_022006927.1	1.1762	1.3089	0.89862
XP_022009213.1	XP_022018622.1	1.3456	1.4972	0.89874
XP_022002421.1	XP_022006327.1	1.2528	1.3938	0.89884
XP_021988229.1	XP_022021911.1	1.252	1.3927	0.89897
XP_021993551.1	XP_022006927.1	1.6509	1.8361	0.89913
XP_021984981.2	XP_021993222.1	1.6316	1.8125	0.90019
XP_021985239.1	XP_021989736.1	1.1921	1.3241	0.90031
XP_021981407.1	XP_021988953.1	1.2581	1.3971	0.90051
XP_022002418.1	XP_022029738.1	0.5282	0.5865	0.9006
XP_021972810.1	XP_021995117.1	1.148	1.2745	0.90075
XP_021986500.1	XP_022008276.2	1.0606	1.177	0.9011
XP_022002415.1	XP_022010616.1	0.8671	0.9619	0.90145
XP_021993622.1	XP_022010296.1	1.4179	1.5729	0.90146
XP_021973855.1	XP_022026963.1	1.7569	1.9487	0.90158
XP_021981395.1	XP_021992525.1	1.0582	1.1729	0.90221
XP_021976022.1	XP_021984981.2	1.3683	1.5165	0.90227
XP_021973855.1	XP_022021913.1	1.7224	1.9085	0.90249
XP_021973855.1	XP_022021914.1	1.7224	1.9085	0.90249
XP_021973316.1	XP_022006927.1	1.4676	1.6247	0.90331
XP_021973855.1	XP_022021911.1	1.6778	1.8572	0.9034
XP_021993052.1	XP_022006668.1	1.0907	1.2071	0.90357
XP_021985239.1	XP_022013558.1	0.8817	0.9753	0.90403
XP_021973316.1	XP_022013558.1	1.1184	1.2369	0.9042
XP_022029622.1	XP_022029740.1	1.1121	1.2296	0.90444
XP_021984766.1	XP_022039074.2	1.2716	1.4056	0.90467
XP_021973855.1	XP_022039074.2	1.7623	1.9476	0.90486
XP_021973855.1	XP_022006926.1	1.6136	1.7824	0.9053
XP_021988229.1	XP_022034230.1	1.233	1.3617	0.90549
XP_022000224.2	XP_022026963.1	1.365	1.5073	0.90559
XP_021968895.1	XP_021993052.1	1.0317	1.1392	0.90564
XP_021989737.1	XP_022000196.1	1.2346	1.3632	0.90566
XP_022001254.1	XP_022026964.1	1.2702	1.4019	0.90606
XP_021978842.1	XP_022002418.1	1.1278	1.2441	0.90652
XP_021984766.1	XP_035842399.1	1.4365	1.5846	0.90654
XP_022018622.1	XP_022035551.1	1.6603	1.831	0.90677
XP_022018622.1	XP_022035552.1	1.6603	1.831	0.90677
XP_021972810.1	XP_021989735.1	1.2356	1.3626	0.9068
XP_021993622.1	XP_022013558.1	1.3665	1.5055	0.90767
XP_022015084.1	XP_035842399.1	1.6174	1.7812	0.90804
XP_021992525.1	XP_022010296.1	1.2968	1.4276	0.90838
XP_021978807.1	XP_022002419.1	0.9962	1.096	0.90894
XP_021981395.1	XP_022037775.1	1.254	1.3795	0.90903
XP_021999258.1	XP_022035551.1	1.3125	1.4435	0.90925
XP_022001254.1	XP_022004979.1	1.4044	1.5445	0.90929
XP_021993220.1	XP_022022487.2	1.3982	1.537	0.90969
XP_021989737.1	XP_022009213.1	0.7382	0.8114	0.90979
XP_021987457.1	XP_021993551.1	1.5189	1.6694	0.90985
XP_021981395.1	XP_022035551.1	1.1045	1.2137	0.91003
XP_021981395.1	XP_022001254.1	1.371	1.5065	0.91006
XP_022002420.1	XP_022022487.2	0.9966	1.0944	0.91064
XP_021981388.1	XP_021988953.1	1.1492	1.2595	0.91243

XP_022001254.1	XP_035842399.1	1.746	1.9131	0.91265
XP_021984766.1	XP_022002419.1	1.157	1.2676	0.91275
XP_022010919.1	XP_022029740.1	1.1999	1.3133	0.91365
XP_021973316.1	XP_022037775.1	1.4402	1.5763	0.91366
XP_022004980.1	XP_022010295.2	1.1836	1.2954	0.91369
XP_022010296.1	XP_022021912.1	1.1509	1.2592	0.91399
XP_021978842.1	XP_021993221.1	1.3576	1.485	0.91421
XP_022010291.1	XP_022010296.1	0.6922	0.7571	0.91428
XP_022000196.1	XP_022013239.1	1.6744	1.831	0.91447
XP_021993221.1	XP_021993222.1	1.2453	1.361	0.91499
XP_021993551.1	XP_022002419.1	1.1255	1.2297	0.91526
XP_021988229.1	XP_021993283.1	1.7292	1.8891	0.91536
XP_022000196.1	XP_022010291.1	1.2685	1.3857	0.91542
XP_022000224.2	XP_022035384.1	1.3492	1.4732	0.91583
XP_021988229.1	XP_022021913.1	1.2707	1.3871	0.91608
XP_021988229.1	XP_022021914.1	1.2707	1.3871	0.91608
XP_021993551.1	XP_022002418.1	1.1439	1.2486	0.91615
XP_021989735.1	XP_022039074.2	1.2188	1.3298	0.91653
XP_021972810.1	XP_022010616.1	1.0062	1.0977	0.91664
XP_021981395.1	XP_021989736.1	0.4667	0.5087	0.91744
XP_022005608.1	XP_022026963.1	1.2352	1.3462	0.91755
XP_021987458.1	XP_021999258.1	1.5795	1.7208	0.91789
XP_021993283.1	XP_022013558.1	1.3936	1.5178	0.91817
XP_021993222.1	XP_022008276.2	1.4147	1.5402	0.91852
XP_021984981.2	XP_035838717.1	0.9001	0.9798	0.91866
XP_021973855.1	XP_021989734.1	1.9464	2.1181	0.91894
XP_021988953.1	XP_022009213.1	1.1583	1.2602	0.91914
XP_021969020.1	XP_022021911.1	1.3718	1.4917	0.91962
XP_021969020.1	XP_022021913.1	1.3718	1.4917	0.91962
XP_021969020.1	XP_022021914.1	1.3718	1.4917	0.91962
XP_022026963.1	XP_035842399.1	1.3976	1.5197	0.91966
XP_021989735.1	XP_022008276.2	1.4247	1.549	0.91975
XP_021989736.1	XP_022035384.1	1.2276	1.3345	0.9199
XP_021973316.1	XP_021999258.1	1.4539	1.5805	0.9199
XP_021973011.1	XP_022021911.1	1.3515	1.469	0.92001
XP_021973011.1	XP_022021913.1	1.3515	1.469	0.92001
XP_021973011.1	XP_022021914.1	1.3515	1.469	0.92001
XP_022010292.1	XP_022013239.1	0.9961	1.0826	0.9201
XP_021995117.1	XP_021999258.1	1.3353	1.4501	0.92083
XP_021993222.1	XP_022034230.1	1.6418	1.7827	0.92096
XP_022002416.1	XP_022013239.1	0.9554	1.0373	0.92105
XP_021992525.1	XP_021995116.1	1.3705	1.4862	0.92215
XP_021992525.1	XP_022008276.2	1.1768	1.2761	0.92218
XP_022010292.1	XP_022021912.1	1.3053	1.4153	0.92228
XP_022010294.1	XP_022013182.1	1.3296	1.4414	0.92244
XP_021987457.1	XP_022037775.1	1.5955	1.7296	0.92247
XP_021984766.1	XP_022029739.1	1.2313	1.3346	0.9226
XP_022035384.1	XP_022039074.2	1.4585	1.5806	0.92275
XP_021981395.1	XP_021993551.1	1.448	1.5692	0.92276
XP_022010291.1	XP_035833070.1	1.3258	1.4362	0.92313
XP_021993221.1	XP_021993620.1	0.8317	0.9008	0.92329
XP_021993551.1	XP_022013183.1	1.6186	1.7524	0.92365
XP_021978807.1	XP_021984766.1	1.1876	1.2857	0.9237
XP_021972810.1	XP_022039085.1	0.678	0.7337	0.92408
XP_022005608.1	XP_022035551.1	1.2519	1.3547	0.92412
XP_022005608.1	XP_022035552.1	1.2519	1.3547	0.92412
XP_021992525.1	XP_022021913.1	1.4253	1.5423	0.92414
XP_021993283.1	XP_021993551.1	1.451	1.5701	0.92414
XP_021978842.1	XP_021985239.1	1.2942	1.4001	0.92436
XP_021989733.1	XP_022029740.1	1.1731	1.269	0.92443
XP_022005608.1	XP_022010295.2	1.2563	1.3587	0.92463
XP_021995116.1	XP_022009213.1	1.6034	1.7337	0.92484
XP_021993551.1	XP_022002417.1	1.161	1.2551	0.92503
XP_022006668.1	XP_022006927.1	1.1723	1.2671	0.92518
XP_021987457.1	XP_022010292.1	1.2238	1.3214	0.92614
XP_021973011.1	XP_021993052.1	1.0918	1.1785	0.92643
XP_022005608.1	XP_022029622.1	1.1068	1.1944	0.92666
XP_021981388.1	XP_021993283.1	1.449	1.5633	0.92689
XP_021976022.1	XP_022033162.1	1.2252	1.3216	0.92706
XP_021981388.1	XP_022039074.2	1.3003	1.4026	0.92706
XP_022000224.2	XP_022029740.1	0.9213	0.9937	0.92714
XP_022002416.1	XP_022029738.1	0.5134	0.5537	0.92722
XP_022002417.1	XP_022029738.1	0.5134	0.5537	0.92722
XP_022013183.1	XP_035842399.1	1.6207	1.7478	0.92728
XP_021973316.1	XP_022004455.1	1.1694	1.2611	0.92729
XP_021984981.2	XP_022004979.1	1.1704	1.2618	0.92756
XP_021984766.1	XP_022033162.1	1.2484	1.3458	0.92763
XP_021993052.1	XP_022006926.1	1.0209	1.1002	0.92792
XP_021976022.1	XP_022010291.1	1.2042	1.2974	0.92816
XP_022013182.1	XP_022013239.1	1.5234	1.6404	0.92868
XP_021993622.1	XP_022008276.2	1.6908	1.8201	0.92896
XP_022010919.1	XP_022013239.1	1.4223	1.5307	0.92918
XP_022029739.1	XP_022029740.1	0.4706	0.5064	0.9293
XP_021993621.2	XP_022029622.1	1.473	1.5847	0.92951
XP_021973854.1	XP_022002415.1	1.1899	1.2798	0.92975
XP_021993220.1	XP_035833070.1	1.4087	1.5148	0.92996
XP_035833070.1	XP_035835830.1	1.1146	1.1983	0.93015
XP_022010616.1	XP_022035551.1	1.1629	1.2499	0.93039

XP_022010616.1	XP_022035552.1	1.1629	1.2499	0.93039
XP_021999258.1	XP_035838717.1	1.2861	1.3816	0.93088
XP_021989735.1	XP_022006327.1	1.3465	1.4462	0.93106
XP_021984766.1	XP_022021912.1	1.478	1.5873	0.93114
XP_021981407.1	XP_035838717.1	0.6808	0.7306	0.93184
XP_022006668.1	XP_022034230.1	1.5792	1.6935	0.93251
XP_021993222.1	XP_035838717.1	1.644	1.7629	0.93255
XP_022006668.1	XP_022014870.1	1.307	1.4015	0.93257
XP_021999258.1	XP_022029622.1	1.3022	1.396	0.93281
XP_021978807.1	XP_021978842.1	1.1702	1.2539	0.93325
XP_022005608.1	XP_035835830.1	1.2058	1.2919	0.93335
XP_021973316.1	XP_022033162.1	1.3938	1.4932	0.93343
XP_021999258.1	XP_022022835.1	1.027	1.0992	0.93432
XP_021968895.1	XP_022026963.1	1.1675	1.2491	0.93467
XP_022010101.1	XP_022021913.1	1.4335	1.5336	0.93473
XP_022010101.1	XP_022021914.1	1.4335	1.5336	0.93473
XP_021978807.1	XP_022021912.1	1.3049	1.3959	0.93481
XP_021976022.1	XP_022001254.1	1.4225	1.5213	0.93506
XP_021993551.1	XP_021993620.1	1.4815	1.5841	0.93523
XP_021999258.1	XP_022010101.1	1.4019	1.4989	0.93529
XP_021993221.1	XP_022014870.1	1.8252	1.9514	0.93533
XP_021993221.1	XP_022029738.1	1.2814	1.3695	0.93567
XP_021993052.1	XP_021993283.1	1.4891	1.5914	0.93572
XP_021993622.1	XP_022000224.2	1.5508	1.657	0.93591
XP_021993622.1	XP_022013560.1	1.5044	1.6053	0.93715
XP_022002415.1	XP_035842399.1	1.1134	1.1878	0.93736
XP_021993551.1	XP_035835830.1	1.2321	1.3141	0.9376
XP_021993551.1	XP_022010291.1	1.4074	1.5008	0.93777
XP_021976022.1	XP_022035552.1	1.482	1.579	0.93857
XP_021973316.1	XP_022035384.1	1.2482	1.3298	0.93864
XP_021973855.1	XP_022010292.1	1.4436	1.5378	0.93874
XP_022006927.1	XP_022013239.1	1.2987	1.3826	0.93932
XP_021989733.1	XP_021989735.1	0.1851	0.197	0.93959
XP_021978807.1	XP_022006668.1	1.1895	1.2656	0.93987
XP_021981395.1	XP_021989737.1	0.5094	0.5419	0.94003
XP_021999258.1	XP_022018622.1	1.463	1.556	0.94023
XP_021978842.1	XP_035835830.1	1.2375	1.3159	0.94042
XP_021993052.1	XP_021993222.1	1.3593	1.445	0.94069
XP_021972810.1	XP_022002416.1	0.6767	0.7193	0.94078
XP_021993283.1	XP_022010292.1	1.4024	1.4905	0.94089
XP_021999258.1	XP_022010919.1	1.5628	1.6602	0.94133
XP_022010101.1	XP_022021911.1	1.4128	1.5007	0.94143
XP_021988229.1	XP_035842399.1	1.5621	1.6585	0.94188
XP_021978807.1	XP_022002418.1	1.0134	1.0751	0.94261
XP_021973316.1	XP_021993551.1	1.7152	1.8193	0.94278
XP_021981626.1	XP_022006668.1	1.4279	1.5142	0.94301
XP_022013183.1	XP_022035551.1	1.0455	1.1086	0.94308
XP_021993221.1	XP_022035384.1	1.9987	2.1192	0.94314
XP_022013239.1	XP_022022487.2	1.498	1.5878	0.94344
XP_021981407.1	XP_022022487.2	1.288	1.3652	0.94345
XP_021989735.1	XP_021989737.1	0.3121	0.3308	0.94347
XP_021993622.1	XP_022010291.1	1.3214	1.4002	0.94372
XP_021978807.1	XP_022002415.1	0.9876	1.0462	0.94399
XP_021984981.2	XP_021989737.1	0.764	0.8093	0.94403
XP_022004980.1	XP_022035384.1	1.6495	1.7473	0.94403
XP_022004980.1	XP_022010292.1	1.1151	1.1805	0.9446
XP_021989736.1	XP_021993220.1	1.5997	1.6933	0.94472
XP_021969020.1	XP_021973855.1	1.9711	2.0855	0.94515
XP_022022835.1	XP_022035384.1	1.3419	1.4197	0.9452
XP_022006926.1	XP_022022487.2	1.1855	1.2542	0.94522
XP_021972810.1	XP_035835830.1	0.1838	0.1944	0.94547
XP_022002418.1	XP_022010616.1	0.9075	0.9595	0.94581
XP_021999258.1	XP_022004455.1	1.197	1.2655	0.94587
XP_021973855.1	XP_022006927.1	1.295	1.369	0.94595
XP_021981626.1	XP_021984981.2	1.2929	1.3665	0.94614
XP_021978842.1	XP_022022487.2	1.1374	1.2019	0.94633
XP_021978842.1	XP_022029740.1	1.347	1.4232	0.94646
XP_021969020.1	XP_022039085.1	1.6275	1.7192	0.94666
XP_021976022.1	XP_022029739.1	1.2978	1.3709	0.94668
XP_021989736.1	XP_022001254.1	1.2272	1.2953	0.94743
XP_021988953.1	XP_022013182.1	1.251	1.3203	0.94751
XP_022005608.1	XP_022006927.1	1.2403	1.3089	0.94759
XP_021981388.1	XP_022001254.1	1.1995	1.2653	0.948
XP_021987457.1	XP_022018622.1	1.3909	1.467	0.94813
XP_022004979.1	XP_022026963.1	1.2151	1.2812	0.94841
XP_022014870.1	XP_035838717.1	0.9735	1.0264	0.94846
XP_021987457.1	XP_021993621.2	1.1058	1.1658	0.94853
XP_021987458.1	XP_021993621.2	1.1058	1.1658	0.94853
XP_021995116.1	XP_022039085.1	1.671	1.7609	0.94895
XP_022005608.1	XP_022008276.2	1.4958	1.5756	0.94935
XP_021969020.1	XP_022022487.2	1.5561	1.6383	0.94983
XP_022009213.1	XP_022039074.2	1.2269	1.2914	0.95005
XP_022013182.1	XP_022035551.1	1.0128	1.0657	0.95036
XP_021973011.1	XP_022021912.1	1.2683	1.3345	0.95039
XP_021992525.1	XP_022021912.1	1.4048	1.4779	0.95054
XP_022010919.1	XP_022021911.1	1.4325	1.5061	0.95113
XP_022010295.2	XP_022021913.1	1.2026	1.2642	0.95127
XP_022010295.2	XP_022021914.1	1.2026	1.2642	0.95127

XP_021995116.1	XP_022021913.1	1.6493	1.7337	0.95132
XP_021995116.1	XP_022021914.1	1.6493	1.7337	0.95132
XP_021993221.1	XP_035835830.1	1.3264	1.3939	0.95157
XP_021993622.1	XP_022010919.1	1.8444	1.9379	0.95175
XP_021978842.1	XP_022010919.1	1.3422	1.4102	0.95178
XP_021988229.1	XP_022010294.1	0.9562	1.0044	0.95201
XP_021981407.1	XP_021989737.1	0.4268	0.4483	0.95204
XP_021988953.1	XP_022035551.1	1.1532	1.2111	0.95219
XP_021988953.1	XP_022035552.1	1.1532	1.2111	0.95219
XP_021984981.2	XP_022008276.2	1.2968	1.3617	0.95234
XP_022002421.1	XP_022010616.1	0.8918	0.9362	0.95257
XP_021989736.1	XP_022029622.1	1.1845	1.2429	0.95301
XP_021995116.1	XP_022006327.1	1.4643	1.5352	0.95382
XP_021972810.1	XP_022026964.1	1.212	1.2704	0.95403
XP_022013183.1	XP_022021911.1	1.4233	1.4918	0.95408
XP_021987458.1	XP_021993551.1	1.5815	1.6564	0.95478
XP_022006927.1	XP_022029622.1	1.3374	1.4005	0.95494
XP_021973316.1	XP_022006668.1	1.5865	1.6613	0.95498
XP_021984766.1	XP_022037775.1	1.3595	1.4228	0.95551
XP_021988953.1	XP_022013183.1	1.3012	1.3613	0.95585
XP_022013182.1	XP_022021912.1	1.3803	1.4435	0.95622
XP_021999258.1	XP_022029740.1	1.0326	1.0788	0.95717
XP_022005608.1	XP_022014870.1	1.4023	1.4638	0.95799
XP_021993222.1	XP_022021913.1	1.6652	1.7378	0.95822
XP_022010919.1	XP_022039085.1	1.194	1.2451	0.95896
XP_022029739.1	XP_035842399.1	1.317	1.3725	0.95956
XP_022010291.1	XP_035842399.1	1.3565	1.4125	0.96035
XP_021973855.1	XP_022013182.1	1.7199	1.7904	0.96062
XP_021993052.1	XP_022037775.1	1.2377	1.2871	0.96162
XP_022001254.1	XP_022014870.1	1.2284	1.2769	0.96202
XP_021989733.1	XP_021999258.1	1.3005	1.3518	0.96205
XP_021987458.1	XP_022018622.1	1.4125	1.468	0.96219
XP_022021913.1	XP_022039074.2	1.415	1.4703	0.96239
XP_022021914.1	XP_022039074.2	1.415	1.4703	0.96239
XP_021989735.1	XP_021992525.1	1.0131	1.0516	0.96339
XP_022000224.2	XP_022010101.1	1.3502	1.4013	0.96353
XP_021984981.2	XP_022029622.1	1.3404	1.3909	0.96369
XP_021993221.1	XP_022037775.1	1.5494	1.6075	0.96386
XP_021981407.1	XP_022009213.1	0.6838	0.7094	0.96391
XP_022004980.1	XP_022039085.1	1.356	1.4065	0.9641
XP_021973316.1	XP_022006327.1	1.5948	1.6537	0.96438
XP_021984766.1	XP_022010101.1	1.2961	1.3438	0.9645
XP_021989737.1	XP_022029622.1	1.1382	1.179	0.96539
XP_021989734.1	XP_022001254.1	1.1874	1.2299	0.96544
XP_021986500.1	XP_022029740.1	0.9792	1.0142	0.96549
XP_021989733.1	XP_022006927.1	1.288	1.334	0.96552
XP_021976022.1	XP_022018622.1	1.3373	1.385	0.96556
XP_021973854.1	XP_022022835.1	1.4845	1.5373	0.96565
XP_021989734.1	XP_022029622.1	1.1759	1.2176	0.96575
XP_021989737.1	XP_021999258.1	1.2825	1.3278	0.96588
XP_021981388.1	XP_022006927.1	1.3649	1.4131	0.96589
XP_022021911.1	XP_022039074.2	1.3992	1.4477	0.9665
XP_022010295.2	XP_022021912.1	1.1317	1.1706	0.96677
XP_022010616.1	XP_035835830.1	1.1243	1.1629	0.96681
XP_021969020.1	XP_021993220.1	1.6991	1.7572	0.96694
XP_022013183.1	XP_022021913.1	1.4501	1.4995	0.96706
XP_021968895.1	XP_021985239.1	0.851	0.8792	0.96793
XP_021995116.1	XP_022013182.1	1.3977	1.444	0.96794
XP_021993221.1	XP_021995116.1	1.2589	1.3005	0.96801
XP_021989736.1	XP_022006927.1	1.1827	1.2217	0.96808
XP_021995117.1	XP_022039074.2	1.1175	1.1531	0.96913
XP_021972810.1	XP_021976022.1	1.5257	1.5724	0.9703
XP_021973316.1	XP_022029738.1	1.2457	1.2838	0.97032
XP_021976022.1	XP_022035551.1	1.4941	1.5397	0.97038
XP_021993551.1	XP_022033162.1	1.7862	1.8407	0.97039
XP_021973854.1	XP_021981395.1	1.6851	1.7364	0.97046
XP_021984981.2	XP_021989735.1	0.8316	0.8568	0.97059
XP_021976022.1	XP_021978807.1	1.4144	1.4568	0.9709
XP_021981388.1	XP_021993551.1	1.3849	1.4254	0.97159
XP_021995116.1	XP_022005608.1	1.3657	1.4056	0.97161
XP_022022835.1	XP_022029738.1	1.1452	1.1775	0.97257
XP_021978842.1	XP_022002417.1	1.1427	1.1748	0.97268
XP_022006926.1	XP_022013182.1	1.5694	1.6134	0.97273
XP_021989733.1	XP_022009213.1	0.7844	0.8063	0.97284
XP_022013183.1	XP_035838717.1	1.098	1.1281	0.97332
XP_022006668.1	XP_022039074.2	1.2285	1.2618	0.97361
XP_021973316.1	XP_022013560.1	1.2489	1.2827	0.97365
XP_021989737.1	XP_022010291.1	1.1151	1.1451	0.9738
XP_021978842.1	XP_021999258.1	1.1956	1.2273	0.97417
XP_021981395.1	XP_021993283.1	1.4911	1.5302	0.97445
XP_021984766.1	XP_021993283.1	1.9832	2.0343	0.97488
XP_021989733.1	XP_035838717.1	0.7813	0.801	0.97541
XP_021978842.1	XP_022006327.1	1.4832	1.5203	0.9756
XP_021981407.1	XP_022001254.1	1.4019	1.4369	0.97564
XP_022004980.1	XP_022006668.1	2.0421	2.0921	0.9761
XP_022035551.1	XP_035838717.1	1.1802	1.2085	0.97658
XP_021993551.1	XP_022014870.1	1.4187	1.452	0.97707
XP_021995116.1	XP_022021911.1	1.6017	1.6382	0.97772

XP_021993551.1	XP_022002416.1	1.1653	1.1914	0.97809
XP_021993620.1	XP_022026963.1	1.287	1.3154	0.97841
XP_021969020.1	XP_022006926.1	1.5739	1.6084	0.97855
XP_022004455.1	XP_022029740.1	0.4072	0.4161	0.97861
XP_021973855.1	XP_021999258.1	1.6403	1.6745	0.97958
XP_021978842.1	XP_022029622.1	1.5275	1.5567	0.98124
XP_022002421.1	XP_035842399.1	1.2961	1.3208	0.9813
XP_022035552.1	XP_035838717.1	1.1775	1.1997	0.9815
XP_021993283.1	XP_022022487.2	1.6109	1.6408	0.98178
XP_022008275.1	XP_022009213.1	1.1765	1.1982	0.98189
XP_022013239.1	XP_022035552.1	1.6864	1.7175	0.98189
XP_022004455.1	XP_022013560.1	0.6204	0.6315	0.98242
XP_021969020.1	XP_022018622.1	1.2905	1.3133	0.98264
XP_022013183.1	XP_022035552.1	1.0509	1.069	0.98307
XP_021988953.1	XP_022010919.1	1.5281	1.5544	0.98308
XP_021989735.1	XP_021999258.1	1.3601	1.3832	0.9833
XP_021993222.1	XP_022022487.2	1.6912	1.7192	0.98371
XP_021978842.1	XP_022009213.1	1.2142	1.2339	0.98403
XP_021986500.1	XP_022001254.1	0.9612	0.9767	0.98413
XP_022006327.1	XP_022039074.2	1.3894	1.4118	0.98413
XP_021988953.1	XP_022008276.2	1.5726	1.5978	0.98423
XP_021993283.1	XP_022029739.1	1.5922	1.6174	0.98442
XP_021989737.1	XP_022022487.2	1.2913	1.3113	0.98475
XP_022008276.2	XP_022037775.1	1.3827	1.4039	0.9849
XP_021995116.1	XP_022035551.1	1.8344	1.8625	0.98491
XP_021995116.1	XP_022035552.1	1.8344	1.8625	0.98491
XP_022013239.1	XP_022035551.1	1.672	1.6971	0.98521
XP_022013183.1	XP_022021914.1	1.4641	1.4848	0.98606
XP_021989733.1	XP_022029622.1	1.1678	1.1841	0.98623
XP_022010919.1	XP_022021913.1	1.465	1.4854	0.98627
XP_022010919.1	XP_022021914.1	1.465	1.4854	0.98627
XP_022006926.1	XP_022021912.1	1.8365	1.8592	0.98779
XP_021993283.1	XP_022010291.1	1.3974	1.4141	0.98819
XP_021988953.1	XP_021993220.1	1.7184	1.7384	0.9885
XP_022013560.1	XP_035833070.1	1.4479	1.4639	0.98907
XP_021973316.1	XP_021984766.1	1.26	1.2732	0.98963
XP_021985239.1	XP_022029740.1	0.9293	0.9388	0.98988
XP_022013182.1	XP_022035552.1	1.0176	1.0274	0.99046
XP_021993622.1	XP_022004455.1	1.1552	1.1663	0.99048
XP_021993551.1	XP_022013182.1	1.6443	1.6599	0.9906
XP_022006327.1	XP_022021912.1	1.4741	1.4866	0.99159
XP_021984981.2	XP_022009213.1	0.9425	0.9502	0.9919
XP_021995116.1	XP_022039074.2	1.5668	1.5796	0.9919
XP_021978807.1	XP_021993283.1	1.4796	1.4894	0.99342
XP_022006668.1	XP_022013183.1	1.3684	1.3774	0.99347
XP_021978842.1	XP_022010101.1	1.312	1.3202	0.99379
XP_021992525.1	XP_021993622.1	1.63	1.6397	0.99408
XP_021984766.1	XP_022015084.1	1.1763	1.1832	0.99417
XP_021989735.1	XP_022035384.1	1.1952	1.2015	0.99476
XP_022013558.1	XP_022021911.1	1.2032	1.2093	0.99496
XP_022013558.1	XP_022021913.1	1.2032	1.2093	0.99496
XP_022013558.1	XP_022021914.1	1.2032	1.2093	0.99496
XP_022000196.1	XP_035842399.1	1.5138	1.5212	0.99514
XP_021993221.1	XP_022021911.1	1.4896	1.4964	0.99546
XP_021973855.1	XP_022000196.1	2.0477	2.0558	0.99606
XP_022004980.1	XP_022013239.1	1.431	1.4365	0.99617
XP_021989733.1	XP_021989737.1	0.2948	0.2959	0.99628
XP_022000196.1	XP_022035551.1	1.6466	1.6527	0.99631
XP_021981407.1	XP_021989735.1	0.428	0.4295	0.99651
XP_021981388.1	XP_022022487.2	1.2824	1.2863	0.99697
XP_022022487.2	XP_022026964.1	1.3514	1.3548	0.99749
XP_022002421.1	XP_022013239.1	1.1075	1.1102	0.99757
XP_022000196.1	XP_022035384.1	1.2728	1.2748	0.99843
XP_021993622.1	XP_022029622.1	1.4305	1.4318	0.99909
XP_022006927.1	XP_022035551.1	1.2471	1.2481	0.9992
XP_021981395.1	XP_021989733.1	0.4446	0.4449	0.99933
XP_022013183.1	XP_022035384.1	1.3641	1.3646	0.99963
XP_021988953.1	XP_021993221.1	1.6479	1.6479	1
XP_022013182.1	XP_035842399.1	1.6479	1.6479	1
XP_022021911.1	XP_022037775.1	1.199	1.1987	1.00025
XP_022021913.1	XP_022037775.1	1.199	1.1987	1.00025
XP_022021914.1	XP_022037775.1	1.199	1.1987	1.00025
XP_021989735.1	XP_021993220.1	1.6785	1.6764	1.00125
XP_021993222.1	XP_021993621.2	0.8437	0.8426	1.00131
XP_022001254.1	XP_022029622.1	1.3334	1.3316	1.00135
XP_022021912.1	XP_022039074.2	1.3132	1.3113	1.00145
XP_021973854.1	XP_021981407.1	1.5418	1.5391	1.00175
XP_021988229.1	XP_021993551.1	1.482	1.4792	1.00189
XP_021993222.1	XP_022000224.2	1.5487	1.5449	1.00246
XP_021988229.1	XP_022022835.1	1.1716	1.1686	1.00257
XP_021981626.1	XP_022015084.1	1.5836	1.5793	1.00272
XP_022035384.1	XP_022039085.1	1.7807	1.7758	1.00276
XP_021973855.1	XP_022018622.1	1.7437	1.7384	1.00305
XP_021989734.1	XP_021989735.1	0.1755	0.1749	1.00343
XP_022013182.1	XP_035838717.1	1.0888	1.085	1.0035
XP_021993622.1	XP_035842399.1	1.9566	1.9493	1.00374
XP_022022487.2	XP_022022835.1	1.4832	1.4773	1.00399
XP_022021911.1	XP_022035551.1	1.442	1.4362	1.00404

XP_022021913.1	XP_022035551.1	1.442	1.4362	1.00404
XP_022021914.1	XP_022035551.1	1.442	1.4362	1.00404
XP_022005608.1	XP_022013182.1	1.145	1.1399	1.00447
XP_022005608.1	XP_022013183.1	1.145	1.1399	1.00447
XP_021988229.1	XP_022000224.2	1.101	1.0959	1.00465
XP_022035384.1	XP_022035551.1	1.469	1.4614	1.0052
XP_021972810.1	XP_022018622.1	1.2637	1.2569	1.00541
XP_021987457.1	XP_022013558.1	1.2992	1.2921	1.00549
XP_022022835.1	XP_035833070.1	1.7479	1.7378	1.00581
XP_021973854.1	XP_021989737.1	1.3169	1.3089	1.00611
XP_021989734.1	XP_021989737.1	0.2623	0.2607	1.00614
XP_021993551.1	XP_022002415.1	1.1738	1.1665	1.00626
XP_021968895.1	XP_021969020.1	1.5249	1.5152	1.0064
XP_021978842.1	XP_022013182.1	1.2949	1.2861	1.00684
XP_021976022.1	XP_021993052.1	1.4952	1.485	1.00687
XP_021989736.1	XP_022029740.1	1.1642	1.1557	1.00735
XP_021993221.1	XP_022021914.1	1.5109	1.4994	1.00767
XP_021989734.1	XP_021999258.1	1.3058	1.2957	1.0078
XP_021981407.1	XP_021993283.1	1.4021	1.3907	1.0082
XP_021976022.1	XP_021986500.1	1.2242	1.2142	1.00824
XP_022002417.1	XP_022002418.1	0.0483	0.0479	1.00835
XP_022002415.1	XP_022029738.1	0.5407	0.5362	1.00839
XP_021993620.1	XP_022006327.1	1.3398	1.3285	1.00851
XP_021985239.1	XP_022006668.1	1.1747	1.1646	1.00867
XP_021989735.1	XP_021993222.1	1.5465	1.5331	1.00874
XP_022021911.1	XP_022039085.1	1.4964	1.4833	1.00883
XP_022000196.1	XP_022037775.1	1.3089	1.2974	1.00886
XP_021973855.1	XP_022029738.1	1.4496	1.4367	1.00898
XP_021973316.1	XP_021985239.1	1.2016	1.1906	1.00924
XP_021993283.1	XP_022009213.1	1.5496	1.5348	1.00964
XP_021993283.1	XP_022013560.1	1.5983	1.5827	1.00986
XP_021989735.1	XP_022039085.1	1.2598	1.2475	1.00986
XP_021993220.1	XP_021993622.1	1.6651	1.6479	1.01044
XP_021978842.1	XP_022033162.1	1.2009	1.1881	1.01077
XP_022000196.1	XP_022035552.1	1.6335	1.6151	1.01139
XP_022002415.1	XP_022013239.1	0.9642	0.9531	1.01165
XP_022010101.1	XP_022010616.1	1.1809	1.1673	1.01165
XP_021989734.1	XP_021992525.1	0.9803	0.969	1.01166
XP_021978842.1	XP_022013183.1	1.2485	1.2341	1.01167
XP_022010292.1	XP_022010296.1	0.1592	0.1573	1.01208
XP_022014870.1	XP_022026964.1	1.2517	1.2366	1.01221
XP_022006327.1	XP_022010292.1	1.2567	1.2413	1.01241
XP_021978842.1	XP_021993283.1	1.5143	1.4957	1.01244
XP_022021913.1	XP_022039085.1	1.5006	1.4819	1.01262
XP_022021914.1	XP_022039085.1	1.5006	1.4819	1.01262
XP_022014870.1	XP_022026963.1	1.6235	1.6028	1.01291
XP_022010295.2	XP_022021911.1	1.1979	1.1824	1.01311
XP_021993052.1	XP_022013558.1	0.9164	0.9045	1.01316
XP_021981407.1	XP_021989733.1	0.3867	0.3816	1.01336
XP_022035384.1	XP_022035552.1	1.4576	1.4382	1.01349
XP_021988953.1	XP_022005608.1	1.2237	1.2071	1.01375
XP_021973854.1	XP_022021912.1	1.7145	1.69	1.0145
XP_022010291.1	XP_022035384.1	1.3515	1.3318	1.01479
XP_021984766.1	XP_021988953.1	1.1294	1.1126	1.0151
XP_021993222.1	XP_022006926.1	1.3768	1.3558	1.01549
XP_021988953.1	XP_022029738.1	1.1961	1.1778	1.01554
XP_021981626.1	XP_022026964.1	1.2235	1.2047	1.01561
XP_021993221.1	XP_022006927.1	1.415	1.3927	1.01601
XP_021986500.1	XP_021993622.1	1.7436	1.7161	1.01602
XP_022021911.1	XP_022022487.2	1.422	1.3995	1.01608
XP_022021913.1	XP_022022487.2	1.422	1.3995	1.01608
XP_022021914.1	XP_022022487.2	1.422	1.3995	1.01608
XP_022006327.1	XP_022006927.1	1.4645	1.4412	1.01617
XP_021973855.1	XP_022002421.1	1.5379	1.5125	1.01679
XP_021973316.1	XP_035833070.1	1.8368	1.8054	1.01739
XP_021973855.1	XP_022021912.1	1.6775	1.6479	1.01796
XP_021985239.1	XP_021993222.1	1.3573	1.3332	1.01808
XP_022006926.1	XP_022014870.1	1.4264	1.4008	1.01828
XP_021972810.1	XP_022010919.1	1.2968	1.2735	1.0183
XP_022010295.2	XP_022013183.1	1.4206	1.3947	1.01857
XP_022006926.1	XP_022037775.1	1.3119	1.2861	1.02006
XP_021987457.1	XP_022005608.1	1.1761	1.1528	1.02021
XP_021993283.1	XP_022013239.1	1.7031	1.6692	1.02031
XP_021973316.1	XP_022021913.1	1.4314	1.4028	1.02039
XP_021981626.1	XP_021993622.1	1.5039	1.4718	1.02181
XP_021995117.1	XP_022006668.1	1.2337	1.2071	1.02204
XP_022014870.1	XP_022039074.2	1.4123	1.3815	1.02229
XP_022006927.1	XP_022035552.1	1.236	1.2089	1.02242
XP_021973854.1	XP_021986500.1	1.4927	1.4594	1.02282
XP_021993220.1	XP_022026963.1	1.304	1.2745	1.02315
XP_021993551.1	XP_022013558.1	1.3262	1.295	1.02409
XP_021988229.1	XP_021995117.1	1.0655	1.0403	1.02422
XP_022021911.1	XP_022035552.1	1.4335	1.399	1.02466
XP_022021913.1	XP_022035552.1	1.4335	1.399	1.02466
XP_022021914.1	XP_022035552.1	1.4335	1.399	1.02466
XP_021993551.1	XP_022029739.1	1.2506	1.2204	1.02475
XP_021984766.1	XP_022029622.1	1.4411	1.4061	1.02489
XP_021988953.1	XP_022010292.1	1.1681	1.1396	1.02501

XP_021976022.1	XP_022006668.1	1.4997	1.4627	1.0253
XP_021989734.1	XP_021993222.1	1.5322	1.4942	1.02543
XP_021973854.1	XP_022002417.1	1.2018	1.1719	1.02551
XP_022026963.1	XP_022033162.1	1.3692	1.335	1.02562
XP_021978842.1	XP_021984766.1	1.226	1.1953	1.02568
XP_021981407.1	XP_021993622.1	1.5138	1.4757	1.02582
XP_022006926.1	XP_035838717.1	1.608	1.5673	1.02597
XP_021973855.1	XP_021993221.1	1.8503	1.803	1.02623
XP_021995116.1	XP_022004979.1	1.6125	1.5712	1.02629
XP_021992525.1	XP_022006926.1	1.526	1.4869	1.0263
XP_021993283.1	XP_022002415.1	1.1782	1.1474	1.02684
XP_021989737.1	XP_022022835.1	1.2219	1.1896	1.02715
XP_022013182.1	XP_022035384.1	1.3382	1.3019	1.02788
XP_022009213.1	XP_022037775.1	1.5171	1.4752	1.0284
XP_021973316.1	XP_022002420.1	1.0981	1.0677	1.02847
XP_021978842.1	XP_022039074.2	1.2144	1.1797	1.02941
XP_022021912.1	XP_022029622.1	1.548	1.5036	1.02953
XP_021973855.1	XP_022006668.1	1.9714	1.9129	1.03058
XP_022002419.1	XP_022010616.1	0.8792	0.8522	1.03168
XP_021987458.1	XP_022005608.1	1.2009	1.1635	1.03214
XP_022026964.1	XP_035842399.1	1.4987	1.4518	1.0323
XP_021993222.1	XP_022021914.1	1.6939	1.6396	1.03312
XP_021973316.1	XP_022021911.1	1.4493	1.4028	1.03315
XP_021973316.1	XP_022021914.1	1.4493	1.4028	1.03315
XP_021992525.1	XP_021999258.1	1.1761	1.1376	1.03384
XP_021993222.1	XP_022021911.1	1.6951	1.6395	1.03391
XP_021993221.1	XP_021993621.2	0.7456	0.7207	1.03455
XP_021993622.1	XP_022001254.1	1.4634	1.4145	1.03457
XP_021976022.1	XP_021985239.1	1.4788	1.4287	1.03507
XP_021973855.1	XP_021989736.1	2.0661	1.996	1.03512
XP_021984766.1	XP_021992525.1	0.9681	0.9351	1.03529
XP_021987457.1	XP_022006927.1	1.3341	1.2882	1.03563
XP_021993220.1	XP_022010291.1	1.2075	1.1659	1.03568
XP_021985239.1	XP_022018622.1	1.1297	1.0907	1.03576
XP_022005608.1	XP_022006327.1	1.556	1.5021	1.03588
XP_021981388.1	XP_021989734.1	0.4239	0.4092	1.03592
XP_022010616.1	XP_035833070.1	1.8526	1.7879	1.03619
XP_021993622.1	XP_022034230.1	1.9261	1.8587	1.03626
XP_021993220.1	XP_022006927.1	1.423	1.3725	1.03679
XP_022026964.1	XP_022029740.1	1.1561	1.114	1.03779
XP_021984981.2	XP_022015084.1	1.3778	1.3273	1.03805
XP_021973011.1	XP_022029622.1	1.2903	1.2429	1.03814
XP_021973854.1	XP_022039085.1	1.5828	1.5241	1.03851
XP_021973011.1	XP_022010292.1	0.9807	0.9442	1.03866
XP_022010919.1	XP_022021912.1	1.3996	1.3466	1.03936
XP_021973854.1	XP_022002416.1	1.212	1.1658	1.03963
XP_021987457.1	XP_021993052.1	1.2426	1.1951	1.03975
XP_021993622.1	XP_022006327.1	1.6203	1.5577	1.04019
XP_022000224.2	XP_022013239.1	1.595	1.5332	1.04031
XP_021973854.1	XP_021995116.1	1.4824	1.4248	1.04043
XP_021969020.1	XP_022010101.1	1.5004	1.4412	1.04108
XP_021978842.1	XP_022022835.1	1.4175	1.3612	1.04136
XP_021993622.1	XP_022008275.1	1.6623	1.5962	1.04141
XP_021973854.1	XP_021993221.1	1.8113	1.7383	1.042
XP_021989735.1	XP_022009213.1	0.8285	0.7943	1.04306
XP_021978842.1	XP_022006926.1	1.4215	1.3624	1.04338
XP_021987457.1	XP_021993220.1	1.2992	1.2451	1.04345
XP_021973855.1	XP_022022835.1	1.5506	1.486	1.04347
XP_021981626.1	XP_022035384.1	1.7661	1.6917	1.04398
XP_021993551.1	XP_022000224.2	1.6666	1.5958	1.04437
XP_021973316.1	XP_021978807.1	1.3845	1.3256	1.04443
XP_021973855.1	XP_022029740.1	1.4085	1.3484	1.04457
XP_022013239.1	XP_022014870.1	1.3733	1.314	1.04513
XP_021978807.1	XP_021988953.1	1.3856	1.3251	1.04566
XP_022009213.1	XP_022014870.1	0.9688	0.9264	1.04577
XP_021972810.1	XP_021993622.1	1.5479	1.4796	1.04616
XP_021993283.1	XP_021995116.1	1.6904	1.6155	1.04636
XP_021993551.1	XP_022006926.1	1.7663	1.6878	1.04651
XP_021999258.1	XP_022022487.2	1.1799	1.1265	1.0474
XP_021988953.1	XP_022001254.1	1.4229	1.3582	1.04764
XP_021978807.1	XP_022029738.1	1.0181	0.9718	1.04764
XP_021969020.1	XP_022000196.1	1.5465	1.4757	1.04798
XP_021976022.1	XP_022000196.1	1.4047	1.3402	1.04813
XP_021987458.1	XP_022006927.1	1.3255	1.2637	1.0489
XP_021987458.1	XP_021993052.1	1.2642	1.2047	1.04939
XP_021993221.1	XP_022021913.1	1.5431	1.47	1.04973
XP_021993221.1	XP_022013560.1	1.4173	1.3495	1.05024
XP_021973316.1	XP_022039074.2	1.4541	1.3844	1.05035
XP_022000196.1	XP_022029738.1	1.1776	1.1211	1.0504
XP_021992525.1	XP_022035552.1	0.8911	0.8476	1.05132
XP_021989735.1	XP_022001254.1	1.2599	1.1982	1.05149
XP_021993551.1	XP_022010292.1	1.3481	1.282	1.05156
XP_021973855.1	XP_022010296.1	1.4719	1.3994	1.05181
XP_021984981.2	XP_022018622.1	1.2147	1.1548	1.05187
XP_021993551.1	XP_022013239.1	1.653	1.5714	1.05193
XP_021989733.1	XP_021989734.1	0.081	0.077	1.05195
XP_022001254.1	XP_022006926.1	1.2532	1.1911	1.05214
XP_021992525.1	XP_021993551.1	1.6515	1.5692	1.05245

XP_021993551.1	XP_022029738.1	1.4739	1.3994	1.05324
XP_021976022.1	XP_022029622.1	1.5783	1.4983	1.05339
XP_021993220.1	XP_022021911.1	1.6675	1.5829	1.05345
XP_021993052.1	XP_022001254.1	1.1287	1.0713	1.05358
XP_021993220.1	XP_022013182.1	1.6077	1.5247	1.05444
XP_021973011.1	XP_021999258.1	1.2637	1.198	1.05484
XP_021989736.1	XP_022009213.1	0.7825	0.7417	1.05501
XP_021984766.1	XP_022035551.1	1.2237	1.1597	1.05519
XP_021984766.1	XP_022035552.1	1.2237	1.1597	1.05519
XP_021976022.1	XP_021989737.1	1.3527	1.28	1.0568
XP_021969020.1	XP_022013239.1	1.5109	1.4292	1.05716
XP_021973855.1	XP_022013183.1	1.7949	1.6975	1.05738
XP_022001254.1	XP_022035552.1	1.3905	1.3147	1.05766
XP_022006927.1	XP_022018622.1	1.247	1.1786	1.05803
XP_022002419.1	XP_022029740.1	0.3261	0.308	1.05877
XP_021984981.2	XP_021993620.1	1.16	1.0955	1.05888
XP_021976022.1	XP_022029740.1	1.2149	1.1468	1.05938
XP_022006927.1	XP_022010919.1	1.2279	1.1589	1.05954
XP_022029740.1	XP_022035551.1	1.2006	1.1331	1.05957
XP_022029740.1	XP_022035552.1	1.2006	1.1331	1.05957
XP_022002421.1	XP_022039085.1	0.7286	0.6876	1.05963
XP_021989734.1	XP_021989736.1	0.1713	0.1616	1.06002
XP_021999258.1	XP_022037775.1	1.4001	1.3198	1.06084
XP_021989735.1	XP_022013182.1	1.0548	0.9935	1.0617
XP_021984766.1	XP_021988229.1	1.3145	1.2377	1.06205
XP_021976022.1	XP_021993622.1	1.7072	1.6068	1.06248
XP_021973316.1	XP_022010101.1	1.285	1.2092	1.06269
XP_022022487.2	XP_022026963.1	1.2673	1.1925	1.06273
XP_021993221.1	XP_022013239.1	0.9505	0.894	1.0632
XP_021993220.1	XP_022013183.1	1.5686	1.4749	1.06353
XP_022006668.1	XP_035838717.1	1.5558	1.4627	1.06365
XP_021984766.1	XP_022010295.2	1.0423	0.9798	1.06379
XP_022009213.1	XP_022013239.1	1.3859	1.3026	1.06395
XP_022021911.1	XP_022034230.1	1.1771	1.1061	1.06419
XP_022021913.1	XP_022034230.1	1.1771	1.1061	1.06419
XP_022021914.1	XP_022034230.1	1.1771	1.1061	1.06419
XP_021989735.1	XP_035838717.1	0.8487	0.7975	1.0642
XP_021995116.1	XP_022008276.2	1.4382	1.3514	1.06423
XP_021981388.1	XP_021992525.1	0.9675	0.9087	1.06471
XP_022013183.1	XP_022013560.1	1.4047	1.3188	1.06513
XP_022001254.1	XP_022035551.1	1.3839	1.2991	1.06528
XP_021993222.1	XP_021993622.1	1.0423	0.9779	1.06586
XP_021989733.1	XP_022037775.1	1.3775	1.292	1.06618
XP_021984766.1	XP_022022835.1	1.4061	1.3188	1.0662
XP_021984981.2	XP_021989733.1	0.898	0.8421	1.06638
XP_021988953.1	XP_022010291.1	1.4305	1.3414	1.06642
XP_021993220.1	XP_035842399.1	1.9628	1.8404	1.06651
XP_021973011.1	XP_022013558.1	0.936	0.8776	1.06655
XP_021993621.2	XP_022013239.1	0.6744	0.6322	1.06675
XP_021988953.1	XP_022022487.2	1.5852	1.4837	1.06841
XP_021993052.1	XP_035842399.1	1.4586	1.3652	1.06841
XP_021992525.1	XP_021993283.1	1.5494	1.4501	1.06848
XP_022013183.1	XP_022021912.1	1.4066	1.316	1.06884
XP_021999258.1	XP_022013183.1	1.2541	1.1733	1.06887
XP_022010616.1	XP_022013239.1	1.6163	1.5121	1.06891
XP_021989736.1	XP_035838717.1	0.7941	0.7425	1.06949
XP_021993551.1	XP_022010101.1	1.6094	1.5046	1.06965
XP_022002418.1	XP_022013239.1	0.9922	0.9272	1.0701
XP_022026963.1	XP_022035384.1	1.586	1.482	1.07018
XP_021988953.1	XP_035833070.1	1.5081	1.4087	1.07056
XP_022021912.1	XP_022035551.1	1.5089	1.4089	1.07098
XP_022021912.1	XP_022035552.1	1.5089	1.4089	1.07098
XP_022001254.1	XP_022039074.2	1.4121	1.3174	1.07188
XP_021981388.1	XP_021989733.1	0.4265	0.3976	1.07269
XP_021993222.1	XP_022009213.1	1.6186	1.5081	1.07327
XP_021989737.1	XP_021992525.1	1.1104	1.0343	1.07358
XP_021973316.1	XP_021978842.1	1.5835	1.4743	1.07407
XP_022009213.1	XP_035833070.1	1.4743	1.3717	1.0748
XP_022010101.1	XP_022010919.1	1.4679	1.3652	1.07523
XP_021987457.1	XP_035842399.1	1.8656	1.7347	1.07546
XP_021993221.1	XP_022006668.1	1.5814	1.4703	1.07556
XP_021993220.1	XP_022021913.1	1.7114	1.5904	1.07608
XP_021989733.1	XP_021992525.1	0.9994	0.9281	1.07682
XP_021999258.1	XP_022009213.1	1.3677	1.2701	1.07684
XP_021973855.1	XP_022033162.1	1.5971	1.4826	1.07723
XP_022006668.1	XP_022033162.1	1.4092	1.3074	1.07786
XP_021989736.1	XP_021993222.1	1.5109	1.4013	1.07821
XP_021993220.1	XP_022021914.1	1.6417	1.5218	1.07879
XP_021989734.1	XP_022009213.1	0.8234	0.7632	1.07888
XP_021973011.1	XP_022029738.1	1.1078	1.0259	1.07983
XP_021984766.1	XP_022006927.1	1.4077	1.3034	1.08002
XP_021993052.1	XP_022006327.1	1.3789	1.2766	1.08013
XP_022013558.1	XP_022021912.1	1.2262	1.1348	1.08054
XP_021984766.1	XP_021993221.1	2.0678	1.911	1.08205
XP_021988953.1	XP_022018622.1	1.31	1.2105	1.0822
XP_021993551.1	XP_022010295.2	1.415	1.3064	1.08313
XP_022013239.1	XP_035838717.1	1.4089	1.3007	1.08319
XP_021993052.1	XP_022013239.1	1.3323	1.2291	1.08396

XP_022000224.2	XP_022006926.1	1.1967	1.1038	1.08416
XP_021993220.1	XP_021993620.1	0.7682	0.7085	1.08426
XP_021987458.1	XP_021993220.1	1.2966	1.1955	1.08457
XP_021973316.1	XP_021993052.1	1.2666	1.1678	1.0846
XP_021993220.1	XP_022005608.1	1.1571	1.0668	1.08465
XP_021984981.2	XP_021989734.1	0.8939	0.824	1.08483
XP_022010919.1	XP_022035384.1	1.4106	1.2991	1.08583
XP_021969020.1	XP_021999258.1	1.6213	1.4925	1.0863
XP_022006327.1	XP_022013558.1	1.1875	1.0929	1.08656
XP_021978842.1	XP_022000196.1	1.4361	1.3209	1.08721
XP_022001254.1	XP_022008276.2	1.4522	1.3348	1.08795
XP_022005608.1	XP_022013239.1	1.127	1.0346	1.08931
XP_021993283.1	XP_022000224.2	1.8042	1.6561	1.08943
XP_022010291.1	XP_022010616.1	0.9658	0.8865	1.08945
XP_021989734.1	XP_035838717.1	0.8274	0.7587	1.09055
XP_021985239.1	XP_021993221.1	1.4294	1.3104	1.09081
XP_021973011.1	XP_021984766.1	1.3988	1.2815	1.09153
XP_021988953.1	XP_022010295.2	1.1359	1.0406	1.09158
XP_022009213.1	XP_022013183.1	1.0972	1.0048	1.09196
XP_021993551.1	XP_022010296.1	1.3145	1.2033	1.09241
XP_021973011.1	XP_022002421.1	1.1465	1.0485	1.09347
XP_021984981.2	XP_022026964.1	1.3345	1.2198	1.09403
XP_022026964.1	XP_022035384.1	1.4521	1.3267	1.09452
XP_021995117.1	XP_022000196.1	1.3629	1.2452	1.09452
XP_022005608.1	XP_022010296.1	1.1006	1.0053	1.0948
XP_022010101.1	XP_022021912.1	1.5705	1.434	1.09519
XP_021995116.1	XP_022013183.1	1.4102	1.2876	1.09522
XP_021992525.1	XP_022006668.1	1.2392	1.1313	1.09538
XP_021993283.1	XP_021993622.1	2.4599	2.2449	1.09577
XP_022021912.1	XP_022034230.1	1.2228	1.1158	1.0959
XP_021993551.1	XP_022026964.1	1.3648	1.244	1.09711
XP_021993622.1	XP_022035552.1	1.741	1.5867	1.09725
XP_021989737.1	XP_022035384.1	1.3634	1.2416	1.0981
XP_021969020.1	XP_022035551.1	2.0299	1.848	1.09843
XP_021969020.1	XP_022035552.1	2.0299	1.848	1.09843
XP_021985239.1	XP_022039074.2	1.0643	0.9684	1.09903
XP_021993622.1	XP_022035551.1	1.6844	1.5312	1.10005
XP_022010294.1	XP_022013560.1	0.145	0.1318	1.10015
XP_022022835.1	XP_035838717.1	1.1995	1.0899	1.10056
XP_021989733.1	XP_021993222.1	1.6321	1.4829	1.10061
XP_021986500.1	XP_022000224.2	1.5998	1.4524	1.10149
XP_022005608.1	XP_022035384.1	1.2845	1.1655	1.1021
XP_021978842.1	XP_022035551.1	1.2984	1.1772	1.10296
XP_021978842.1	XP_022035552.1	1.2984	1.1772	1.10296
XP_021993622.1	XP_022010292.1	1.3557	1.2289	1.10318
XP_021988229.1	XP_021993221.1	1.6683	1.5122	1.10323
XP_022010295.2	XP_022013182.1	1.4766	1.338	1.10359
XP_021993220.1	XP_022035384.1	1.6515	1.4956	1.10424
XP_022006927.1	XP_022013183.1	1.4638	1.3249	1.10484
XP_021993222.1	XP_022013239.1	1.4353	1.2981	1.10569
XP_021981395.1	XP_021989734.1	0.4348	0.3932	1.1058
XP_022006327.1	XP_022006668.1	1.5562	1.4066	1.10636
XP_021978807.1	XP_022002421.1	1.1009	0.995	1.10643
XP_021976022.1	XP_022000224.2	1.2208	1.1032	1.1066
XP_021986500.1	XP_022037775.1	0.7457	0.6735	1.1072
XP_021973855.1	XP_021995117.1	1.7851	1.6119	1.10745
XP_021972810.1	XP_022035384.1	1.5368	1.3876	1.10752
XP_021999258.1	XP_022013182.1	1.2179	1.0995	1.10769
XP_021976022.1	XP_021981626.1	1.7249	1.5548	1.1094
XP_022005608.1	XP_022029740.1	0.9849	0.8872	1.11012
XP_021981407.1	XP_021989736.1	0.4254	0.3831	1.11042
XP_021981407.1	XP_021989734.1	0.383	0.3449	1.11047
XP_022013239.1	XP_022039085.1	1.4248	1.283	1.11052
XP_021993551.1	XP_022018622.1	1.5929	1.4315	1.11275
XP_022001254.1	XP_022013239.1	1.3886	1.2478	1.11284
XP_021992525.1	XP_035838717.1	0.9852	0.8844	1.11398
XP_021973316.1	XP_022015084.1	1.8055	1.6193	1.11499
XP_021976022.1	XP_022002421.1	1.3877	1.2444	1.11516
XP_021973854.1	XP_022029740.1	1.1691	1.0481	1.11545
XP_021973855.1	XP_022010101.1	1.385	1.2404	1.11658
XP_021989737.1	XP_022037775.1	1.327	1.1883	1.11672
XP_021985239.1	XP_022001254.1	1.5347	1.3742	1.1168
XP_021973854.1	XP_021993621.2	1.7708	1.585	1.11722
XP_021993052.1	XP_022035551.1	1.4315	1.2808	1.11766
XP_021993052.1	XP_022035552.1	1.4315	1.2808	1.11766
XP_021981626.1	XP_022022487.2	1.2767	1.1418	1.11815
XP_021993220.1	XP_022018622.1	1.2449	1.112	1.11951
XP_021981626.1	XP_022000196.1	1.7114	1.5286	1.11959
XP_022006927.1	XP_035838717.1	1.4091	1.258	1.12011
XP_021973855.1	XP_022029622.1	1.7583	1.5689	1.12072
XP_021993551.1	XP_021995117.1	1.2673	1.1306	1.12091
XP_021989734.1	XP_022013182.1	1.0057	0.8972	1.12093
XP_021993221.1	XP_022026964.1	1.1128	0.9921	1.12166
XP_021984981.2	XP_021999258.1	1.2191	1.0865	1.12204
XP_021993283.1	XP_022014870.1	1.5479	1.3789	1.12256
XP_022009213.1	XP_022013182.1	1.0883	0.9694	1.12265
XP_022029738.1	XP_022029740.1	0.5365	0.4778	1.12285
XP_021973011.1	XP_021973316.1	1.4919	1.3279	1.1235

XP_021993551.1	XP_022010294.1	1.3457	1.1962	1.12498
XP_022010294.1	XP_035833070.1	1.4288	1.2697	1.12531
XP_021978807.1	XP_022006926.1	1.0278	0.9129	1.12586
XP_021993222.1	XP_022010616.1	1.3821	1.2271	1.12631
XP_021993052.1	XP_022013182.1	1.4494	1.2863	1.1268
XP_021993221.1	XP_022022835.1	1.4078	1.2486	1.1275
XP_022008275.1	XP_035833070.1	1.4153	1.2552	1.12755
XP_022000224.2	XP_022014870.1	1.1334	1.0051	1.12765
XP_021993283.1	XP_022035551.1	1.9149	1.6981	1.12767
XP_021993283.1	XP_022035552.1	1.9149	1.6981	1.12767
XP_021993622.1	XP_035835830.1	1.2444	1.1032	1.12799
XP_022022835.1	XP_022037775.1	1.4061	1.2458	1.12867
XP_021978842.1	XP_035833070.1	1.6675	1.4773	1.12875
XP_022005608.1	XP_035833070.1	1.478	1.3089	1.12919
XP_021987458.1	XP_035842399.1	1.8914	1.675	1.12919
XP_021973855.1	XP_021993222.1	2.103	1.8618	1.12955
XP_021973316.1	XP_022010919.1	1.7954	1.5885	1.13025
XP_021988953.1	XP_022033162.1	1.3831	1.2227	1.13119
XP_021993052.1	XP_022000224.2	1.3124	1.16	1.13138
XP_021992525.1	XP_022029622.1	1.3842	1.2227	1.13208
XP_021989733.1	XP_022013182.1	0.9993	0.8825	1.13235
XP_021989736.1	XP_021999258.1	1.5049	1.3289	1.13244
XP_021969020.1	XP_021995116.1	1.9375	1.7109	1.13244
XP_021993620.1	XP_022018622.1	1.0754	0.9485	1.13379
XP_021988953.1	XP_021993621.2	1.4101	1.2437	1.13379
XP_022005608.1	XP_035842399.1	1.7545	1.5473	1.13391
XP_021993283.1	XP_022039085.1	1.6751	1.4767	1.13435
XP_021993220.1	XP_022013239.1	0.9611	0.8472	1.13444
XP_021993283.1	XP_021995117.1	1.4003	1.2316	1.13698
XP_021993221.1	XP_022010101.1	1.7671	1.5539	1.1372
XP_022009213.1	XP_022022835.1	1.2601	1.1075	1.13779
XP_021993283.1	XP_021993621.2	2.8498	2.5028	1.13864
XP_021989734.1	XP_021993283.1	1.3466	1.1822	1.13906
XP_021989734.1	XP_022013183.1	0.9929	0.8716	1.13917
XP_021993220.1	XP_022010919.1	1.5593	1.3688	1.13917
XP_021993220.1	XP_022006668.1	1.7462	1.5325	1.13945
XP_022005608.1	XP_022013560.1	1.0968	0.9612	1.14107
XP_021985239.1	XP_022010919.1	1.1325	0.9921	1.14152
XP_022034230.1	XP_022035384.1	1.7732	1.5526	1.14208
XP_021981407.1	XP_022039074.2	1.2919	1.1309	1.14236
XP_022000224.2	XP_022026964.1	1.8498	1.6179	1.14333
XP_022000224.2	XP_022010616.1	1.2914	1.1281	1.14476
XP_021973855.1	XP_022002417.1	1.7718	1.5474	1.14502
XP_022006926.1	XP_022009213.1	1.6273	1.4204	1.14566
XP_021989735.1	XP_022037775.1	1.3718	1.1967	1.14632
XP_021969020.1	XP_021973854.1	1.9267	1.6781	1.14814
XP_021981395.1	XP_021993221.1	1.4349	1.2495	1.14838
XP_022000196.1	XP_022029739.1	1.1212	0.9751	1.14983
XP_021989735.1	XP_022013183.1	1.0624	0.9237	1.15016
XP_021989733.1	XP_022013183.1	0.9863	0.8574	1.15034
XP_022021911.1	XP_022021912.1	0.0872	0.0756	1.15344
XP_022021912.1	XP_022021913.1	0.0872	0.0756	1.15344
XP_022021912.1	XP_022021914.1	0.0872	0.0756	1.15344
XP_021973855.1	XP_021981626.1	2.2499	1.9461	1.15611
XP_022006668.1	XP_022026964.1	1.2836	1.1093	1.15713
XP_021981388.1	XP_022013182.1	1.0354	0.8944	1.15765
XP_022006927.1	XP_022009213.1	1.4104	1.2181	1.15787
XP_022000196.1	XP_022022487.2	1.4438	1.2469	1.15791
XP_021993052.1	XP_022013183.1	1.4588	1.2595	1.15824
XP_021993283.1	XP_022010295.2	1.3913	1.2002	1.15922
XP_021969020.1	XP_022029739.1	1.4623	1.2587	1.16175
XP_021984766.1	XP_022013182.1	0.9844	0.8463	1.16318
XP_022035551.1	XP_022037775.1	1.5147	1.2979	1.16704
XP_022035552.1	XP_022037775.1	1.5147	1.2979	1.16704
XP_021993283.1	XP_022002418.1	1.2857	1.1012	1.16754
XP_021993621.2	XP_022006327.1	1.4811	1.2685	1.1676
XP_021976022.1	XP_022026964.1	1.4169	1.212	1.16906
XP_021973855.1	XP_022002419.1	1.8139	1.5514	1.1692
XP_021995116.1	XP_022026964.1	1.2467	1.0652	1.17039
XP_022006327.1	XP_022013239.1	1.7437	1.4894	1.17074
XP_021993052.1	XP_022010919.1	1.372	1.1718	1.17085
XP_022026963.1	XP_022037775.1	1.7088	1.4594	1.17089
XP_021973316.1	XP_022021912.1	1.5051	1.2852	1.1711
XP_022005608.1	XP_022006668.1	1.7081	1.4579	1.17162
XP_021989736.1	XP_021992525.1	1.0488	0.8928	1.17473
XP_022006668.1	XP_022010919.1	1.2019	1.023	1.17488
XP_021976022.1	XP_022013183.1	1.3495	1.1479	1.17563
XP_021993283.1	XP_022037775.1	1.5172	1.2893	1.17676
XP_021988953.1	XP_022039074.2	1.2178	1.0344	1.1773
XP_021985239.1	XP_022010616.1	1.0923	0.9274	1.17781
XP_022000224.2	XP_022004979.1	1.555	1.3184	1.17946
XP_021978807.1	XP_022001254.1	1.4051	1.1913	1.17947
XP_021973854.1	XP_021993283.1	2.208	1.8718	1.17961
XP_021973316.1	XP_022004980.1	1.7108	1.4501	1.17978
XP_022005608.1	XP_022010292.1	1.1647	0.9868	1.18028
XP_021968895.1	XP_035842399.1	1.6724	1.4153	1.18166
XP_021993621.2	XP_022026963.1	1.0185	0.8615	1.18224
XP_021972810.1	XP_021978842.1	1.5682	1.3256	1.18301

XP_022013239.1	XP_022026964.1	1.1313	0.9546	1.1851
XP_021993221.1	XP_022010616.1	1.3956	1.1772	1.18552
XP_022006668.1	XP_022009213.1	1.5311	1.2901	1.18681
XP_021981395.1	XP_022013182.1	1.1095	0.9342	1.18765
XP_021984766.1	XP_021993052.1	1.3487	1.1337	1.18964
XP_021978807.1	XP_022018622.1	1.3297	1.1175	1.18989
XP_021993220.1	XP_021993283.1	1.9854	1.6678	1.19043
XP_021993621.2	XP_022018622.1	1.1666	0.9799	1.19053
XP_021993221.1	XP_022010919.1	1.3549	1.1377	1.19091
XP_022006327.1	XP_035833070.1	1.9551	1.6416	1.19097
XP_021976022.1	XP_021992525.1	1.3451	1.1292	1.1912
XP_021978807.1	XP_021993052.1	0.9817	0.824	1.19138
XP_021995116.1	XP_022000196.1	1.3091	1.0984	1.19182
XP_022010919.1	XP_022018622.1	1.2152	1.0186	1.19301
XP_021993551.1	XP_021993621.2	1.6644	1.3951	1.19303
XP_021993220.1	XP_021995116.1	1.1989	1.0049	1.19305
XP_021984766.1	XP_022013183.1	1.0526	0.8801	1.196
XP_021993222.1	XP_022018622.1	1.549	1.2941	1.19697
XP_021993222.1	XP_021999258.1	1.9237	1.6057	1.19804
XP_021973855.1	XP_021993620.1	1.5705	1.3096	1.19922
XP_021989737.1	XP_021993220.1	1.6416	1.3684	1.19965
XP_021968895.1	XP_021973855.1	1.4204	1.1831	1.20057
XP_021988953.1	XP_021993222.1	1.7397	1.4474	1.20195
XP_021993283.1	XP_022004980.1	1.6718	1.3901	1.20265
XP_022035384.1	XP_035838717.1	1.3164	1.0945	1.20274
XP_021993283.1	XP_022002416.1	1.2624	1.0495	1.20286
XP_021986500.1	XP_022013182.1	1.2506	1.0378	1.20505
XP_021973855.1	XP_022010294.1	1.5112	1.254	1.2051
XP_022013182.1	XP_022013560.1	1.428	1.1834	1.20669
XP_022001254.1	XP_022037775.1	1.188	0.9834	1.20805
XP_022005608.1	XP_022022487.2	1.3333	1.1029	1.2089
XP_021984981.2	XP_022037775.1	1.3175	1.0894	1.20938
XP_021993221.1	XP_022001254.1	1.5761	1.3029	1.20969
XP_021976022.1	XP_022022487.2	1.5343	1.2681	1.20992
XP_021999258.1	XP_022006927.1	1.3569	1.1214	1.21001
XP_021993221.1	XP_022005608.1	1.523	1.2585	1.21017
XP_021993283.1	XP_022026963.1	1.4049	1.1602	1.21091
XP_021988953.1	XP_022010101.1	1.5755	1.301	1.21099
XP_021972810.1	XP_021993052.1	1.2339	1.0167	1.21363
XP_021992525.1	XP_022001254.1	1.5151	1.2474	1.21461
XP_021989735.1	XP_021993622.1	2.005	1.6479	1.2167
XP_035833070.1	XP_035838717.1	1.6316	1.3408	1.21689
XP_021989737.1	XP_022013182.1	1.0861	0.892	1.2176
XP_021993221.1	XP_022010291.1	1.4038	1.1527	1.21784
XP_022002415.1	XP_022002418.1	0.0531	0.0435	1.22069
XP_021973854.1	XP_022005608.1	1.8616	1.5244	1.2212
XP_021969020.1	XP_035842399.1	1.9459	1.5933	1.2213
XP_021988953.1	XP_022006926.1	1.3438	1.0998	1.22186
XP_021984981.2	XP_022035384.1	1.5342	1.2555	1.22198
XP_021976022.1	XP_022006927.1	1.424	1.1652	1.22211
XP_022006927.1	XP_022022487.2	1.324	1.0831	1.22242
XP_021989736.1	XP_021993622.1	1.8155	1.4851	1.22248
XP_021989736.1	XP_022013182.1	1.0995	0.8986	1.22357
XP_022002416.1	XP_022002418.1	0.0454	0.0371	1.22372
XP_021973855.1	XP_022010291.1	1.4022	1.144	1.2257
XP_021973855.1	XP_022013558.1	1.4022	1.1438	1.22591
XP_021973316.1	XP_022029739.1	1.2854	1.0481	1.22641
XP_021981388.1	XP_022013183.1	1.0222	0.8324	1.22802
XP_021981388.1	XP_021993221.1	1.5253	1.2419	1.2282
XP_021976022.1	XP_022010919.1	1.6464	1.3398	1.22884
XP_021972810.1	XP_021993283.1	1.6579	1.3485	1.22944
XP_022029738.1	XP_022029739.1	0.2131	0.1733	1.22966
XP_021993283.1	XP_022002421.1	1.3877	1.1273	1.23099
XP_021989737.1	XP_021993283.1	1.4033	1.1398	1.23118
XP_021989737.1	XP_021993622.1	1.7438	1.4153	1.23211
XP_021995116.1	XP_035842399.1	1.6612	1.3474	1.23289
XP_021978807.1	XP_022006927.1	1.0712	0.8688	1.23297
XP_021993620.1	XP_022026964.1	1.1629	0.9418	1.23476
XP_021976022.1	XP_022013182.1	1.4141	1.1444	1.23567
XP_022013239.1	XP_022021911.1	1.6629	1.3438	1.23746
XP_022006927.1	XP_022035384.1	1.5167	1.2252	1.23792
XP_021993220.1	XP_022026964.1	1.2047	0.9731	1.238
XP_021973855.1	XP_021987458.1	1.758	1.4196	1.23838
XP_021993052.1	XP_022014870.1	1.2676	1.0234	1.23862
XP_022005608.1	XP_022010291.1	1.1856	0.9564	1.23965
XP_021973855.1	XP_021987457.1	1.7395	1.4024	1.24037
XP_021973855.1	XP_022035384.1	1.798	1.4495	1.24043
XP_021981626.1	XP_021984766.1	1.3537	1.0911	1.24067
XP_022006927.1	XP_022010616.1	0.8947	0.7202	1.24229
XP_021999258.1	XP_022039085.1	1.6893	1.3596	1.2425
XP_021987457.1	XP_022022835.1	1.2295	0.9895	1.24255
XP_021987458.1	XP_022022835.1	1.2086	0.9679	1.24868
XP_021973855.1	XP_022029739.1	1.9991	1.6005	1.24905
XP_021993220.1	XP_021993621.2	0.6004	0.4799	1.25109
XP_021992525.1	XP_021993221.1	1.5147	1.2084	1.25348
XP_021993221.1	XP_022004980.1	1.4187	1.1318	1.25349
XP_022029622.1	XP_035842399.1	1.9483	1.5506	1.25648
XP_022006927.1	XP_022013182.1	1.5299	1.2157	1.25845

XP_022013239.1	XP_022021914.1	1.6552	1.3149	1.2588
XP_021973855.1	XP_021985239.1	1.8572	1.4744	1.25963
XP_021988953.1	XP_021993551.1	1.7028	1.3515	1.25993
XP_022010291.1	XP_022010919.1	1.1273	0.8943	1.26054
XP_021976022.1	XP_022039074.2	1.2616	1.0007	1.26072
XP_022001254.1	XP_022006927.1	1.2586	0.9967	1.26277
XP_022000224.2	XP_022006927.1	1.3314	1.0512	1.26655
XP_021986500.1	XP_021993221.1	1.3619	1.0744	1.26759
XP_022004455.1	XP_022035384.1	1.4755	1.1639	1.26772
XP_021981395.1	XP_022013183.1	1.0929	0.8619	1.26801
XP_022002421.1	XP_022004455.1	0.2474	0.1949	1.26937
XP_021976022.1	XP_022037775.1	1.2387	0.9752	1.2702
XP_021973011.1	XP_021976022.1	1.4915	1.1736	1.27088
XP_022013239.1	XP_022021913.1	1.7106	1.3449	1.27192
XP_022013239.1	XP_022021912.1	1.7847	1.4031	1.27197
XP_022029622.1	XP_022034230.1	1.5157	1.1913	1.27231
XP_021993283.1	XP_022026964.1	1.3904	1.0928	1.27233
XP_022009213.1	XP_022035384.1	1.3028	1.0233	1.27314
XP_021985239.1	XP_021993283.1	1.8598	1.4582	1.27541
XP_021986500.1	XP_022013183.1	1.3276	1.0407	1.27568
XP_021968895.1	XP_021995117.1	1.1981	0.9379	1.27743
XP_021995116.1	XP_022013239.1	1.3255	1.037	1.27821
XP_021973011.1	XP_022010919.1	1.353	1.0553	1.2821
XP_021969020.1	XP_022026963.1	1.6841	1.3135	1.28215
XP_022001254.1	XP_022013183.1	1.421	1.1082	1.28226
XP_021976022.1	XP_022021912.1	1.9888	1.5501	1.28301
XP_021978807.1	XP_022029739.1	1.1683	0.9104	1.28328
XP_021973855.1	XP_022001254.1	2.1952	1.7098	1.28389
XP_021986500.1	XP_022035551.1	1.3673	1.0649	1.28397
XP_021986500.1	XP_022035552.1	1.3673	1.0649	1.28397
XP_021973855.1	XP_022002420.1	1.4435	1.1241	1.28414
XP_021993621.2	XP_022005608.1	1.3642	1.0616	1.28504
XP_021989734.1	XP_021993221.1	1.4905	1.1587	1.28636
XP_022005608.1	XP_022010294.1	1.1599	0.9009	1.28749
XP_022000196.1	XP_022006926.1	1.5705	1.2187	1.28867
XP_021989736.1	XP_022037775.1	1.4283	1.1076	1.28954
XP_021993221.1	XP_035833070.1	1.5323	1.1871	1.29079
XP_021993551.1	XP_021993622.1	1.8734	1.4482	1.29361
XP_021993551.1	XP_022010919.1	1.7366	1.3413	1.29471
XP_021987457.1	XP_021993221.1	1.5681	1.2096	1.29638
XP_021993221.1	XP_022026963.1	1.522	1.1725	1.29808
XP_021973855.1	XP_022002418.1	1.5664	1.205	1.29992
XP_021981388.1	XP_021993622.1	1.5484	1.1902	1.30096
XP_021989736.1	XP_021993283.1	1.2967	0.9952	1.30295
XP_021989737.1	XP_022013183.1	1.0847	0.8316	1.30435
XP_021973316.1	XP_022026964.1	1.444	1.1068	1.30466
XP_021988953.1	XP_021993622.1	1.7942	1.3748	1.30506
XP_021986500.1	XP_021993283.1	1.5583	1.1931	1.30609
XP_021973855.1	XP_022006327.1	0.9877	0.7553	1.30769
XP_021989733.1	XP_021993221.1	1.4427	1.1029	1.3081
XP_021993221.1	XP_021993283.1	1.9006	1.4527	1.30832
XP_022005608.1	XP_022013558.1	1.1618	0.8865	1.31055
XP_021989736.1	XP_022013183.1	1.098	0.8374	1.3112
XP_021973855.1	XP_021993551.1	2.2016	1.6776	1.31235
XP_021973316.1	XP_021976022.1	1.6591	1.2638	1.31279
XP_021989737.1	XP_021993221.1	1.5144	1.147	1.32031
XP_022001254.1	XP_022013182.1	1.3916	1.0538	1.32055
XP_021989733.1	XP_021993622.1	1.7392	1.3169	1.32068
XP_021992525.1	XP_022009213.1	0.9995	0.7567	1.32087
XP_021993283.1	XP_022006927.1	1.6148	1.2219	1.32155
XP_021993221.1	XP_022006327.1	1.8203	1.3772	1.32174
XP_021993220.1	XP_022037775.1	1.8916	1.4307	1.32215
XP_021973855.1	XP_022013560.1	1.3937	1.0538	1.32255
XP_021984981.2	XP_021993220.1	1.5772	1.1925	1.3226
XP_021989736.1	XP_021993221.1	1.5233	1.1485	1.32634
XP_021989733.1	XP_021993283.1	1.3807	1.0397	1.32798
XP_021993220.1	XP_022009213.1	1.437	1.0818	1.32834
XP_022005608.1	XP_022010919.1	1.2896	0.9688	1.33113
XP_021993283.1	XP_022004455.1	1.5456	1.1593	1.33322
XP_021999258.1	XP_022035384.1	1.5264	1.1432	1.3352
XP_021981407.1	XP_021993221.1	1.3999	1.0477	1.33616
XP_021976022.1	XP_021993221.1	1.5036	1.1252	1.3363
XP_021992525.1	XP_021993220.1	1.691	1.2652	1.33655
XP_022006327.1	XP_022010291.1	1.4371	1.0731	1.3392
XP_021981395.1	XP_022022487.2	1.3893	1.0359	1.34115
XP_021993283.1	XP_022029738.1	1.8226	1.3587	1.34143
XP_022021911.1	XP_022026963.1	1.6513	1.2297	1.34285
XP_022021913.1	XP_022026963.1	1.6513	1.2297	1.34285
XP_022021914.1	XP_022026963.1	1.6513	1.2297	1.34285
XP_021993622.1	XP_022039085.1	1.9598	1.4586	1.34362
XP_021984766.1	XP_022005608.1	1.0606	0.7891	1.34406
XP_021993220.1	XP_035838717.1	1.3691	1.0173	1.34582
XP_021993283.1	XP_022002417.1	1.3637	1.0107	1.34926
XP_021973855.1	XP_035835830.1	1.3715	1.0163	1.3495
XP_021973316.1	XP_022005608.1	1.4567	1.073	1.3576
XP_022004979.1	XP_022014870.1	1.5648	1.1522	1.3581
XP_021973855.1	XP_022005608.1	1.9806	1.458	1.35844
XP_022029622.1	XP_022035551.1	1.5323	1.1274	1.35914

XP_022029622.1	XP_022035552.1	1.5323	1.1274	1.35914
XP_021981395.1	XP_021993622.1	1.6846	1.2383	1.36041
XP_021993622.1	XP_022006927.1	1.7554	1.2866	1.36437
XP_021993622.1	XP_022009213.1	1.6714	1.2087	1.38281
XP_022004979.1	XP_022022487.2	1.4607	1.0562	1.38298
XP_021973854.1	XP_035833070.1	2.3476	1.6907	1.38854
XP_021993221.1	XP_022013183.1	1.67	1.1996	1.39213
XP_022022487.2	XP_022034230.1	1.2755	0.9156	1.39308
XP_021976022.1	XP_021993220.1	1.6935	1.215	1.39383
XP_021972810.1	XP_022005608.1	1.5069	1.0798	1.39554
XP_021986500.1	XP_021993222.1	1.47	1.053	1.39601
XP_022010919.1	XP_035833070.1	1.9484	1.3946	1.3971
XP_021973316.1	XP_022013239.1	1.52	1.0868	1.3986
XP_021973855.1	XP_022034230.1	1.8737	1.3396	1.3987
XP_021993283.1	XP_022029740.1	1.4269	1.0198	1.3992
XP_021989734.1	XP_021993622.1	1.8812	1.3427	1.40106
XP_021973855.1	XP_022002415.1	1.593	1.1363	1.40192
XP_022006927.1	XP_022022835.1	1.5205	1.0826	1.40449
XP_021993551.1	XP_022005608.1	1.7734	1.2593	1.40824
XP_021989734.1	XP_022037775.1	1.3406	0.9516	1.40879
XP_021969020.1	XP_021993052.1	1.1488	0.8146	1.41026
XP_021973316.1	XP_021993283.1	1.6583	1.173	1.41373
XP_021992525.1	XP_022006927.1	1.4275	1.0094	1.41421
XP_021993551.1	XP_022026963.1	1.743	1.2324	1.41431
XP_021973855.1	XP_022002416.1	1.6242	1.1481	1.41469
XP_021973855.1	XP_022039085.1	1.7578	1.2421	1.41518
XP_021993622.1	XP_035838717.1	1.6289	1.1447	1.42299
XP_021988229.1	XP_021993052.1	1.5756	1.1009	1.43119
XP_021973011.1	XP_021973855.1	1.674	1.1694	1.4315
XP_021976022.1	XP_021999258.1	0.8889	0.6191	1.43579
XP_021978842.1	XP_022029738.1	1.0551	0.7316	1.44218
XP_021984766.1	XP_021993622.1	2.1973	1.5213	1.44436
XP_021987458.1	XP_021993221.1	1.6252	1.1252	1.44437
XP_021973855.1	XP_022010295.2	1.5091	1.0441	1.44536
XP_021984981.2	XP_021993221.1	1.4811	1.0219	1.44936
XP_021989735.1	XP_021993221.1	1.6003	1.1031	1.45073
XP_021976022.1	XP_021993283.1	2.017	1.3818	1.45969
XP_021993221.1	XP_022013182.1	1.6292	1.1155	1.46051
XP_021976022.1	XP_022005608.1	1.3455	0.9199	1.46266
XP_021973854.1	XP_022010291.1	1.4696	1.0046	1.46287
XP_021985239.1	XP_021993052.1	1.4047	0.9585	1.46552
XP_021976022.1	XP_022014870.1	1.5523	1.0591	1.46568
XP_021972810.1	XP_021973855.1	1.5433	1.0443	1.47783
XP_022005608.1	XP_022037775.1	1.2628	0.8544	1.478
XP_021969020.1	XP_022010919.1	1.5913	1.0713	1.48539
XP_022013239.1	XP_022026963.1	1.3268	0.8874	1.49515
XP_021978807.1	XP_022022487.2	1.6708	1.1121	1.50238
XP_021993283.1	XP_022005608.1	1.4666	0.9644	1.52074
XP_021973855.1	XP_021988229.1	2.1745	1.4187	1.53274
XP_021988953.1	XP_021993052.1	1.2323	0.8019	1.53673
XP_022010616.1	XP_022029740.1	1.013	0.6554	1.54562
XP_022008276.2	XP_022035552.1	1.8488	1.1932	1.54945
XP_021993221.1	XP_035842399.1	2.3631	1.5196	1.55508
XP_022008276.2	XP_022035551.1	1.8217	1.1667	1.56141
XP_022006668.1	XP_022026963.1	1.6363	1.0465	1.56359
XP_021989735.1	XP_021993283.1	1.4866	0.9465	1.57063
XP_021993621.2	XP_022026964.1	0.9838	0.6251	1.57383
XP_021973316.1	XP_021973855.1	2.4596	1.5514	1.58541
XP_021993622.1	XP_022018622.1	1.8356	1.142	1.60736
XP_022013239.1	XP_022018622.1	1.5694	0.9763	1.6075
XP_021993220.1	XP_022022835.1	1.7122	1.0615	1.613
XP_021993551.1	XP_022006668.1	1.7943	1.1086	1.61853
XP_021976022.1	XP_022021911.1	1.9721	1.2183	1.61873
XP_021993221.1	XP_035838717.1	1.7387	1.0504	1.65527
XP_021973316.1	XP_022029740.1	1.4076	0.8459	1.66403
XP_021976022.1	XP_022021914.1	1.9966	1.1809	1.69074
XP_021993221.1	XP_022009213.1	1.659	0.973	1.70504
XP_021993283.1	XP_022006327.1	2.4436	1.4272	1.71216
XP_021976022.1	XP_022035384.1	1.7595	1.0205	1.72415
XP_021986500.1	XP_021999258.1	1.7316	1.0037	1.72522
XP_022001254.1	XP_022006668.1	1.6203	0.9368	1.72961
XP_021976022.1	XP_022021913.1	2.06	1.1547	1.78401
XP_022006668.1	XP_022035384.1	1.6746	0.9322	1.7964
XP_021993283.1	XP_022001254.1	1.7116	0.9482	1.8051
XP_021993222.1	XP_022005608.1	1.4739	0.8077	1.82481
XP_021969020.1	XP_022005608.1	1.732	0.9313	1.85977
XP_021976022.1	XP_021993222.1	2.0304	1.0717	1.89456
XP_022002415.1	XP_022002417.1	0.0314	0.0161	1.95031
XP_021973855.1	XP_035833070.1	1.7125	0.8733	1.96095
XP_021993622.1	XP_022005608.1	1.9556	0.9945	1.96642
XP_021999258.1	XP_022005608.1	1.1965	0.5752	2.08015

Supplementary Table S5: List of differentially expressed lipid transfer proteins in sunflower roots exposed to biotic/abiotic stress

NCBI protein id	NCBI mRNA id	log Fold Change	FDR	Group	Treatment
XP_021988229.1	XM_022132537.2	2.834715039	0.00387003	Group 1	abscisic acid (ABA)
XP_022002419.1	XM_022146727.2	8.021563417	0.012413052	Group 1	
XP_022002421.1	XM_022146729.2	8.186317769	0.006042532	Group 1	
XP_022039085.1	XM_022183393.2	2.511753711	0.042272083	Group 1	
XP_022010291.1	XM_022154599.2	6.266556948	0.010920857	Group 1	
XP_021978842.1	XM_022123150.2	4.240420962	0.002877963	Group 3	
XP_021976022.1	XM_022120330.2	8.466916229	0.025079117	Group 4	
XP_021981388.1	XM_022125696.2	4.14310633	0.006239578	Group 4	
XP_021981407.1	XM_022125715.2	4.513320439	0.003816971	Group 4	
XP_021981626.1	XM_022125934.2	3.712406533	0.017443818	Group 4	
XP_021984981.2	XM_022129289.2	2.46919501	0.014244771	Group 4	
XP_021989733.1	XM_022134041.2	4.682197855	0.001881819	Group 4	
XP_021989734.1	XM_022134042.2	4.240280619	0.015103787	Group 4	
XP_021989735.1	XM_022134043.2	4.677704929	0.001447002	Group 4	
XP_021989736.1	XM_022134044.2	4.424335739	0.000479134	Group 4	
XP_021989737.1	XM_022134045.2	4.870993345	0.000345912	Group 4	
XP_021992525.1	XM_022136833.2	5.119870041	0.00249623	Group 4	
XP_021999258.1	XM_022143566.2	4.951915155	0.001223199	Group 4	
XP_022001254.1	XM_022145562.2	3.631235954	0.010139831	Group 4	
XP_022009213.1	XM_022153521.2	5.208208295	0.001081865	Group 4	
XP_022013182.1	XM_022157490.2	4.961363904	0.041359508	Group 4	
XP_022029622.1	XM_022173930.2	11.29730415	0.000326543	Group 4	
XP_022035551.1	XM_022179859.2	2.841906932	0.034868881	Group 4	
XP_022029622.1	XM_022173930.2	4.784501987	0.034373502	Group 4	ethylene (ACC)
XP_021978807.1	XM_022123115.2	1.784522849	0.003879871	Group 1	auxin (IAA)
XP_021973011.1	XM_022117319.2	2.40737312	0.004250813	Group 3	
XP_021973854.1	XM_022118162.2	1.64039657	0.032908746	Group 3	
XP_022000196.1	XM_022144504.2	1.962624556	0.008497727	Group 3	
XP_022006327.1	XM_022150635.2	1.95107845	0.021490264	Group 3	
XP_022037775.1	XM_022182083.2	2.440186916	0.027866949	Group 3	
XP_021981388.1	XM_022125696.2	-7.869640004	0.01857345	Group 4	
XP_021984981.2	XM_022129289.2	1.688692553	0.041339442	Group 4	
XP_021989734.1	XM_022134042.2	-6.6365625	0.04719478	Group 4	
XP_021989736.1	XM_022134044.2	-2.283604219	0.026583534	Group 4	
XP_021993551.1	XM_022137859.2	2.105354452	0.006676769	Group 4	
XP_021988229.1	XM_022132537.2	2.430023286	0.014848974	Group 1	methyl jasmonate (MeJA)
XP_022039074.2	XM_022183382.2	-2.030430003	0.018062055	Group 1	
XP_021984981.2	XM_022129289.2	3.06292858	0.024043793	Group 4	Saline environment (NaCl)
XP_021989737.1	XM_022134045.2	2.815975745	0.017989441	Group 4	
XP_022009213.1	XM_022153521.2	3.030159904	0.040323592	Group 4	
XP_021989735.1	XM_022134043.2	3.811445451	0.012418785	Group 4	salicylic acid (SA)
XP_021989736.1	XM_022134044.2	3.285999362	0.006314513	Group 4	
XP_021989737.1	XM_022134045.2	3.690486494	0.003701662	Group 4	
XP_022009213.1	XM_022153521.2	3.305522051	0.023768798	Group 4	
XP_022029622.1	XM_022173930.2	5.130494744	0.042188326	Group 4	kinetin (KIN)
